# Repurposing rapid diagnostic tests to detect falsified vaccines in supply chains

**DOI:** 10.1016/j.vaccine.2024.01.019

**Published:** 2024-02-14

**Authors:** Tehmina Bharucha, Bevin Gangadharan, Rebecca Clarke, Laura Gomez Fernandez, Benediktus Yohan Arman, John Walsby-Tickle, Michael Deats, Sara Mosca, Qianqi Lin, Robert Stokes, Susanna Dunachie, Hamid A. Merchant, Audrey Dubot-Pérès, Céline Caillet, James McCullagh, Pavel Matousek, Nicole Zitzmann, Paul N. Newton

**Affiliations:** a Department of Biochemistry, https://ror.org/052gg0110 University of Oxford, Oxford OX1 3QU, UK; b Kavli Institute for Nanoscience Discovery, https://ror.org/052gg0110 University of Oxford, Oxford OX1 3QU, UK; c https://ror.org/045te9e08 Lao-Oxford-Mahosot Hospital-Wellcome Trust Research Unit (LOMWRU), Microbiology Laboratory, Mahosot Hospital, Vientiane, Lao Democratic People’s Republic; d Department of Chemistry, https://ror.org/052gg0110 University of Oxford, Oxford OX1 3TA, UK; e Centre for Tropical Medicine & Global Health, Nuffield Department of Medicine, https://ror.org/052gg0110 University of Oxford, Oxford OX3 7LG, UK; f Central Laser Facility, https://ror.org/00gqx0331 Research Complex at Harwell, https://ror.org/057g20z61 STFC https://ror.org/03gq8fr08 Rutherford-Appleton Laboratory, https://ror.org/001aqnf71 UKRI, Harwell Campus, Didcot OX11 0QX, UK; g Agilent Technologies LDA UK, Becquerel Avenue, Didcot OX11 0RA, UK; h https://ror.org/03fs9z545 Mahidol-Oxford Tropical Medicine Research Unit, Faculty of Tropical Medicine, https://ror.org/01znkr924 Mahidol University, Bangkok 10400, Thailand; i Department of Microbiology and Infectious Diseases, https://ror.org/03h2bh287 Oxford University Hospitals NHS Foundation Trust, Oxford OX3 9DU, UK; j Department of Bioscience, School of Health, Sport and Bioscience, https://ror.org/057jrqr44 University of East London, Water Lane London E15 4LZ, UK; k Department of Pharmacy, School of Applied Sciences, https://ror.org/05t1h8f27 University of Huddersfield, Queensgate, Huddersfield HD1 3DH, UK; l Unité Des Virus Emergents (UVE: https://ror.org/035xkbk20 Aix Marseille Univ, IRD190, https://ror.org/02vjkv261 INSERM 1207), Marseille, France; m https://ror.org/04tp3cz81 Infectious Diseases Data Observatory, Nuffield Department of Medicine, https://ror.org/052gg0110 University of Oxford, Oxford OX3 7LG, UK

**Keywords:** Falsified vaccines, Substandard vaccines, Rapid diagnostic tests, Lateral flow tests, Diagnostics, Screening

## Abstract

Substandard (including degraded) and falsified (SF) vaccines are a relatively neglected issue with serious global implications for public health. This has been highlighted during the rapid and widespread rollout of COVID-19 vaccines. There has been increasing interest in devices to screen for SF non-vaccine medicines including tablets and capsules to empower inspectors and standardise surveillance. However, there has been very limited published research focussed on repurposing or developing new devices for screening for SF vaccines. To our knowledge, rapid diagnostic tests (RDTs) have not been used for this purpose but have important potential for detecting falsified vaccines. We performed a proof-in-principle study to investigate their diagnostic accuracy using a diverse range of RDT-vaccine/falsified vaccine surrogate pairs. In an initial assessment, we demonstrated the utility of four RDTs in detecting seven vaccines. Subsequently, the four RDTs were evaluated by three blinded assessors with seven vaccines and four falsified vaccines surrogates. The results provide preliminary data that RDTs could be used by multiple international organisations, national medicines regulators and vaccine manufacturers/distributors to screen for falsified vaccines in supply chains, aligned with the WHO global ‘Prevent, Detect and Respond’ strategy.

## 1 Introduction

The vital importance of vaccines as cost-effective interventions to prevent and mitigate the impact of numerous infectious diseases has been demonstrated for multiple human and veterinary pathogens, from polio, tetanus and bluetongue virus to COVID-19. It has been estimated that ~5 billion doses of vaccines were produced per year pre-pandemic [1], but at least ~12 billion COVID-19 vaccine doses have been administered globally in just two years from 2021 to 2022 [2]. Once developed, approved by regulatory bodies and mass produced, the major risks to ensure their optimal public health benefit revolve around ensuring access, mitigating vaccine hesitancy and safeguarding their storage and transport under suitable conditions with appropriate administration. Although there has been abundant recent discussion of these issues [3–6], one neglected aspect has been the risk of occurrence and impact of substandard and falsified (SF) vaccines.

Falsified medical products, including vaccines, are those that ‘deliberately and fraudulently misrepresent their identity, composition or source’. In contrast, substandard medical products are ‘authorised medical products that fail to meet either their quality standards or their specifications, or both’ [7]. These may result from gross negligence, unintended errors during the manufacturing process or degradation through inappropriate storage, transport within the supply chain. Both types present a major global health risk through impaired potency and effectiveness, risk of potentially severe adverse events, loss of income, increased spending on healthcare and lead to public mistrust in vaccines, all risking increased vaccine hesitancy globally [8–13].

Vaccines are increasingly important for global public health; inappropriate storage and criminality raise the risk of SF vaccines harming public health. In the ten years before COVID-19 there were many examples of falsified vaccines, including reports from China, Niger, Indonesia, the Philippines, and the World Health Organization (WHO) have issued multiple alerts (https://www.who.int/teams/regulation-prequalification/incidents-and-SF/full-list-of-who-medical-product-alerts) [8,10–13]. There continue to be great concerns that protection of communities and control of the COVID-19 pandemic is potentially impaired by SF vaccines [6].

Up to March 2022 there have been 184 reports of diverted and SF COVID-19 vaccines in the public domain from 48 countries, involving thousands of vaccine doses, and representing significant risks to public health and confidence in vaccines (https://www.tropmedres.ac/files/mpqr-reports/medical-product-quality-report_covid-19_issue15_january-march2022_v1-1.pdf). Great efforts have been and are made to reduce the risk of temperature-induced vaccine degradation in supply chains [14]. Substandard vaccines due to within factory errors have been rare but have also occurred (e.g. the alleged ruin of 400 million doses of COVID vaccine in the USA) [15]. To facilitate detection of falsification, some COVID-19 vaccines packaging includes cryptic security features and it has been argued that all should have unique 2D barcodes (aka global serialisation initiative) on primary and secondary packaging. However, the global infrastructure for this has not yet been fully developed with lack of implementation in most low- and middle-income countries [16].

There has been increasing interest in devices to screen for SF medicines, including tablets and capsules, to empower inspectors and standardise surveillance [17,18]. However, there has been very limited published research focussed on repurposing or developing new devices for screening for SF vaccines. In order to work towards reducing the risk of SF vaccines globally, we therefore have investigated the repurposing of diverse devices for detecting falsified vaccines, with the aim that these could be used by multiple international organisations, national medicines regulators, vaccine manufacturers/distributors, and even point of use pharmacies and hospitals, to screen for falsified vaccines in supply chains, aligned with the WHO global ‘Prevent, Detect and Respond’ strategy [7].

**Fig. 1:**
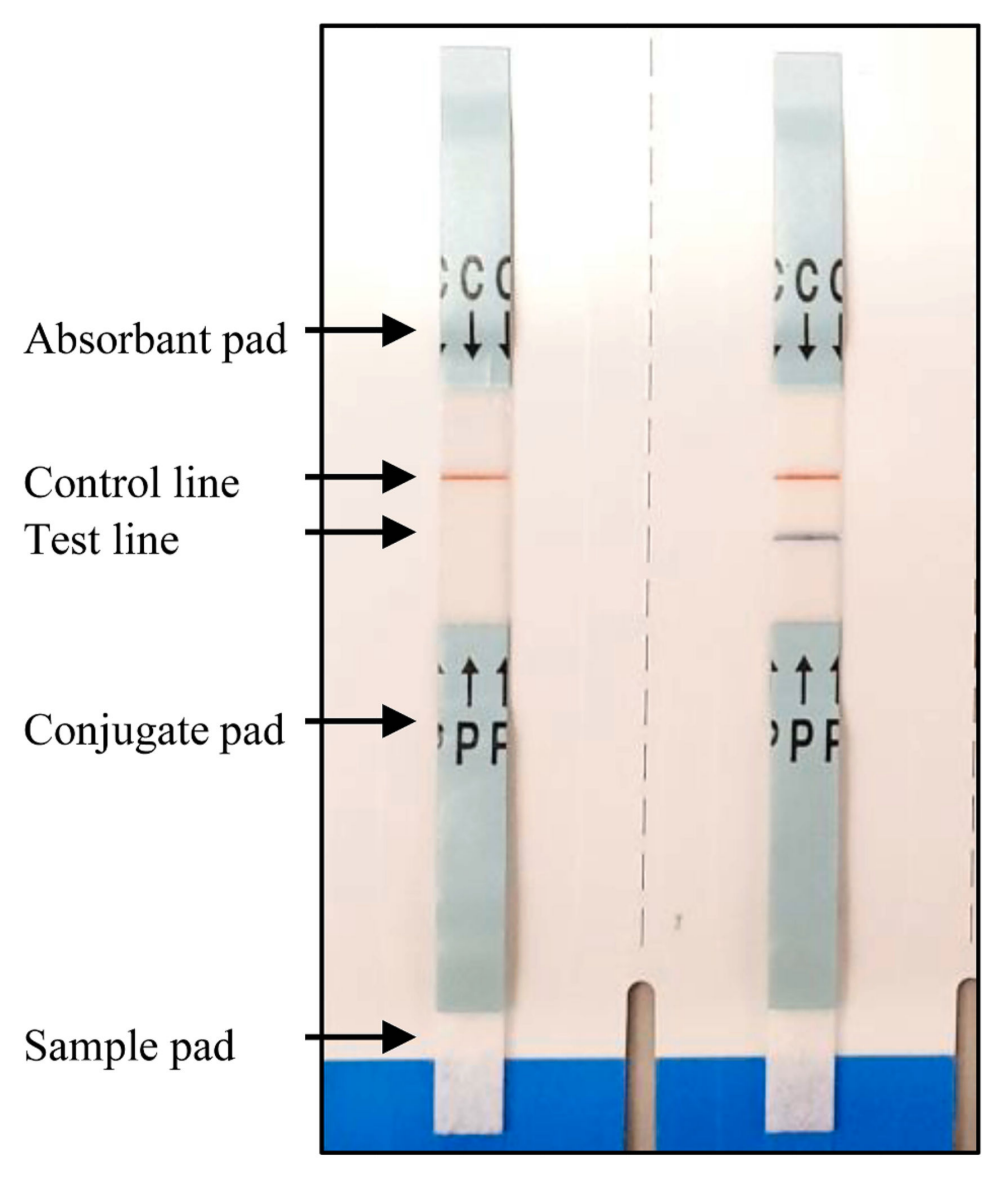
Determine™ HBsAg (Abbott P/N 7D2947) LFT used to test a falsified vaccine surrogate (left) and a Hepatitis B vaccine (right).

We recently evaluated the accuracy of spatially-offset Raman spectroscopy (SORS) to detect falsified vaccines [19]. SORS has the advantage of not requiring the vial or syringe to be opened but is relatively expensive and does not detect vaccine active ingredients directly. We have been exploring diverse technologies that could be used at different positions within supply chains. One novel approach we propose is to utilise rapid diagnostic tests (RDTs), that have been highlighted during the COVID-19 pandemic as having a vital role in clinical diagnostics [20]. The most widely used RDTs are lateral flow tests (LFTs). LFTs are single use and provide rapid results, typically within 15–30 min. They are simple to use and do not need any analytical equipment to interpret. They are inexpensive and have shown high accuracy for the diagnosis of many diseases [21,22]. It is notable that the simplicity of the method means that LFTs can easily be deployed and accessed in remote areas that do not have access to laboratory diagnostics [23]. Also, their wide use globally during the COVID-19 pandemic, means healthcare professionals and public have developed good competency in using these devices for self-testing. Fig. 1 illustrates the typical configuration of a standard LFT; a sample is added onto a sample pad and the analyte of interest (antigen or antibody from the sample), if present, flows through the conjugate pad containing conjugated antibodies against the analyte and binds to a capture antibody immobilised on the test line. If the target antigen is present it is labelled with gold-particle-conjugated antibody and as the sample moves along the device the target is subsequently bound to immobilised antibodies at the test line. A coloured line will be seen.

The first commercial LFT was a urine pregnancy test launched in 1988 [24]. Since then, LFTs have emerged as indispensable for the diagnosis of infectious diseases such as malaria and dengue, particularly in low-and-middle-income countries, and have been widely used during the COVID-19 pandemic, and hence many primary health care workers are familiar with their use. They have also been adapted for a range of other areas of application, both clinical, (for example in the diagnosis and monitoring of chronic diseases such as diabetes, and non-clinical, for example in the identification of bioterrorism agents). Aside from LFTs, a range of other formats for RDTs are available. These include loop mediated isothermal amplification (LAMP), recombinase polymerase amplification (RPA) assays and latex agglutination tests. Latex agglutination test, for example, was originally introduced to assist with the laboratory diagnosis of rheumatoid arthritis, and relies on the interaction of an antigen with antibodies coated on coloured latex beads leading to the agglutination ‘clumping’ of the complex antigen/antibody-beads. It is used for the detection of bacteria associated with meningitis in cerebrospinal fluid samples.

We hypothesised that a number of widely available RDTs manufactured for the diagnosis of infectious diseases could be repurposed for the identification of falsified vaccines. To our knowledge, LFTs have not been used for this purpose, and have important potential. It is much less likely that they will be able to detect substandard vaccines. We aimed to perform a proof-in-principle study to investigate this hypothesis using a diverse range of LFT-vaccine/falsified vaccine surrogate pairs.

## 2 Materials and methods

### 2.1 Vaccine samples

Licenced vaccines used were purchased through the Oxford University Hospital NHS, Foundations Trust pharmacy and stored according to manufacturers’ guidance (Table 1). The samples are listed below including their trade names with manufacturer, batch and expiry date in parentheses. These included two hepatitis B virus vaccines, HBVAXPRO (Merck Sharp & Dohme UK Ltd, U005351 06/23, U033739 08/23) and Engerix B (SmithKline Beecham Ltd AHBVC986AB 11/23, AHBV-C999AL 04/24 and AHBVD044AI 05/24); two *Streptococcus pneumoniae* vaccines, Prevenar 13 (Pfizer Ltd ED3324 06/23) and Pneumovax 23 (Merck Sharp & Dohme UK Ltd UO31935 09/23, UO21322 09/23 and T042608 01/23); two *Neisseria meningitidis* vaccines, Nimenrix (Pfizer Limited DD0524 08/23, DT7089 08/23, ET9885 01/24 and FW7921 10/24) and Menitorix (combined with *Haemophilus influenzae* Glax-oSmithKline UK A76CA413A 07/24); and one *Plasmodium falciparum* vaccine, MSP1 – an experiemental adenovirus-based vaccine developed for malaria (provided in kind by Professor S. Draper, Department of Biochemistry, University of Oxford) [25].

### 2.2 Falsified vaccine surrogates

We used falsified vaccine surrogate samples, based on reports of the contents falsified vaccines in the public domain. These included: 1) tap water (Department of Biochemistry, University of Oxford), 2) saline (0.9 % w/v sodium chloride in sterile water, NaCl; Injection BP Demo S. A Pharmaceutical Industry P/N 24598/0002; Lot 2102386), 3) glucose (5.0 % w/v; B/Braun P/N 03551/0059; batch 22041405) and 4) Amikacin (250 mg/mL MA Holder Tillomed Laboratories Limited P/N 11311/0604; batch ES200079B and FM9809AA) (see Table 2 [19]).

### 2.3 Rapid diagnostic tests (RDTs)

The RDTs selected were those that would be expected to detect vaccines: Determine™ HBsAg (Abbott P/N 7D2947) named hereafter ‘Hep B LFT’, BinaxNOW™ *Streptococcus pneumoniae* Antigen Card (Abbott 710100) ‘Strep. pneumo LFT’ and SD Bioline Rota/Adeno (Abbott 14FK20) ‘Rota/Adeno LFT’. As we were unable to identify a commercially available LFT for the detection of *Neisseria meningitidis*, we tested the widely used Pastorex Meningitis kit (Biorad P/N 61607) ‘Latex agglutination kit’ (Table 1).

### 2.4 Identification

RDTs were tested in accordance with the manufacturers’ recommendations for clinical diagnostic testing, with modifications where needed, for example to adapt an RDT to a vaccine that represented a different sample matrix (Table 1). All samples were tested using LFTs in triplicate except for Pastorex. Vaccines tested with the Pastorex latex agglutination kit, for which there was not sufficient volume available per vial, were only tested once (450 μL vaccine required per card). Different batches were tested based on availability. Images were taken by smartphone (Samsung Galaxy S9) in a standardised position with standardised LED lighting.

### Initial assessment

The numbers of RDTs and vaccine vials tested are set out in Table 2. Results were determined by three assessors independently, without conferring, from images, based on the observation of a control line and the presence or absence of a test line. An indeterminate result was included for kits that did not demonstrate a visible control line. For the agglutination kits, the observation of agglutination ‘clumps’, as described by the manufacturer were interpreted as a positive result.

### Blinded study

**Fig. 2:**
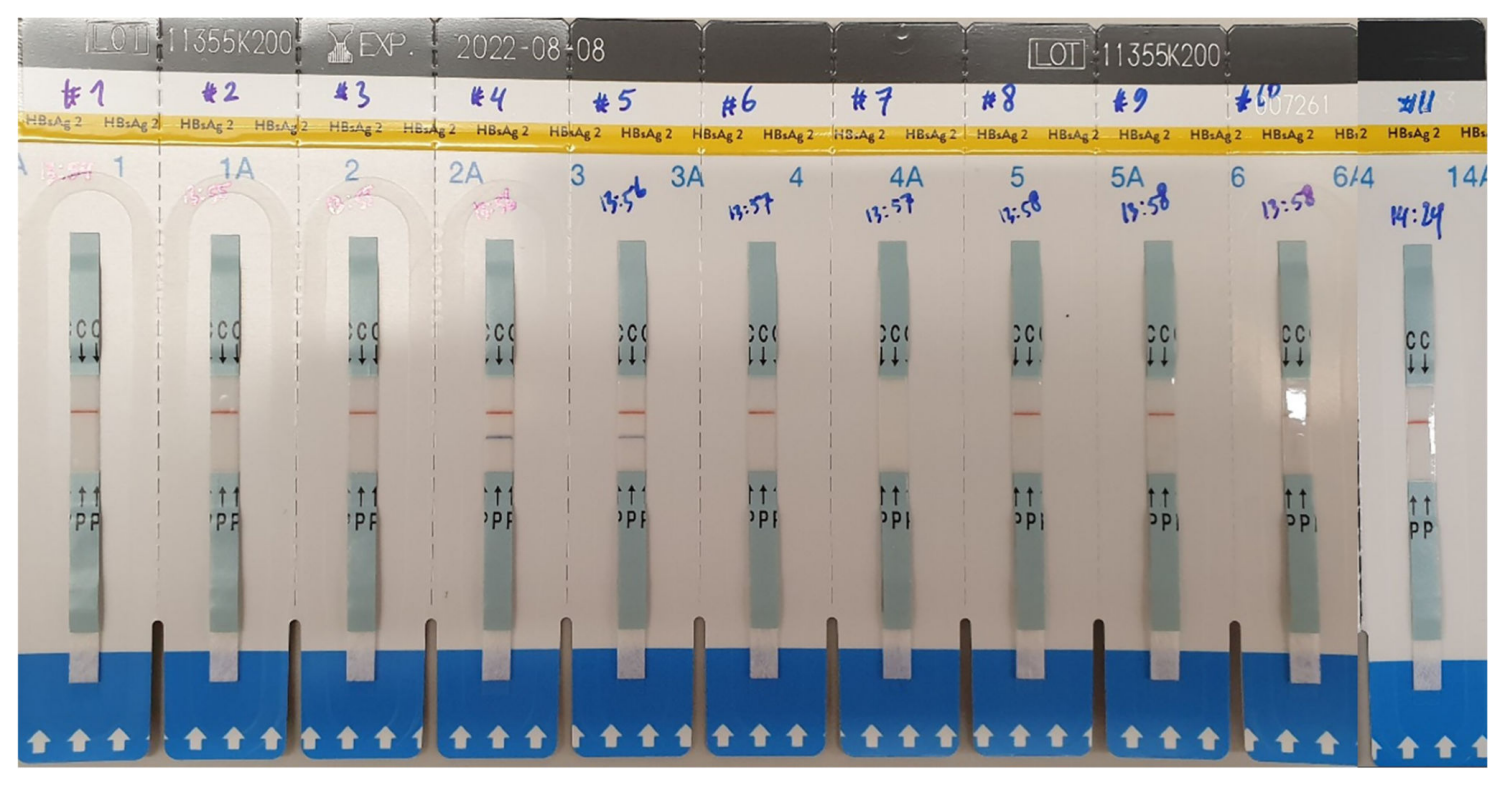
Results of blind testing four falsified vaccine surrogates and seven vaccines, including (#4 and 5) hepatitis B vaccines, with the Determine™ HBsAg RDT.

Eleven additional samples were used in a prospective evaluation by ‘blinded’ assessors, without conferring, of the RDTs, including seven different vaccines (Engerix B, HBVAXPRO, Prevenar-13, Pneumovax-23, Nimenrix, Menitorix and MSP-1) and four different falsified constituents (tap water, 0.9 % saline, 5 % glucose and amikacin), see Fig. 2 for Hep B LFTs. One assessor performed the testing, involving one vial for each type of vaccine testing each type of RDT, and three ‘blinded’ assessors independently read the results. The final result was the overall majority result of the three assessors. Samples that did not produce a control line nor a test line were deemed negative after repeating three times. The overall sample binary classification (‘pass’ or ‘fail’) was used to calculate the sensitivity and specificity for each RDT. Sensitivity was defined as the percentage of true positives (authentic vaccines positive with an RDT) over the total of true positives and false negatives, and specificity as the percentage of true negatives over the total of true negatives and false positives (false surrogate vaccines positive with an RDT).

## 3 Results

### Initial assessment

Initial assessment showed that the RDTs detected all vaccine samples with 100 % agreement between the three assessors, 100 % agreement between replicates of a vial and 100 % agreement between ten replicate vials. All results (photos of the RDTs as captured during testing) are included in supplementary data.

### Blinded study

Further evaluation involved testing each RDT with the seven vaccines listed in Table 1, and the four falsified vaccine surrogates, performed by three independent assessors. An example of the results is presented in Fig. 2. Data on the accuracy of the RDTs are presented in Table 3. Agreement between different assessors was 90.9 % for the Rota/Adeno LFT, 100 % for the *S. pneumoniae* LFTs, 100 % for the Hep B LFTs and 81.8 % for the Latex agglutination kit.

1. AdCh63 MSP1; 2. Prevenar-13; 3. 5 % Glucose; 4. HBVAXPRO; 5. Engerix B; 6. Tap water; 7. Amikacin; 8. Nimenrix; 9. 0.9 % Saline; 10. Pneumovax-23 and 11. Menitorix. Positive results are seen in this figure for samples 4 and 5, both hepatitis b vaccines.

## 4 Discussion

RDTs successfully enabled discrimination of genuine vaccines from falsified vaccine surrogates in 10/11 > 90 % of samples tested. It is not clear why a control line was not seen for amikacin or why there was a weak false positive for the Rota/Adeno LFT with 5 % Glucose; we speculate that this could be due to high sample viscosity for the former, and pH or sugar content for the latter as has been previously reported for soft drinks producing false positive COVID-19 LFTs [26]. Our data support the proposal that existing RDTs can be effectively repurposed for the detection of certain falsified vaccines and provides a novel potential use for RDTs. This is in line with the growing interest in ‘adding value’ to RDTs for new uses, such as the proposition for detection of the markers of resistance in *Salmonella* Typhi [27], detection and genomic surveillance of arboviruses [28,29] and COVID-19 [30]. Further research is, however, needed to investigate the potential of RDTs to detect sub-standard, especially temperature-altered, vaccines.

In addition to their potential diagnostic accuracy, RDTs have the advantages of fulfilling the ASSURED criteria of being affordable, sensitive, specific, user-friendly, rapid, equipment-free, and delivered [21]. This will facilitate their use in distal supply chain locations lacking more sophisticated analysis equipment and/or as part of a multi-technique supply chain monitoring system. For malaria, there is already significant experience in the implementation of multiple RDT systems and global quality control systems (https://www.who.int/teams/global-malaria-programme/case-management/diagnosis/rapid-diagnostic-tests/the-need-for-quality-assurance). There is also a potential link to phone camera readers in fulfilling the REASSURED criteria [21].

The disadvantages of RDT-based systems for detecting vaccine falsification are that they need vaccine vials/syringes to be opened, and are hence destructive, but would be less disadvantageous for multidose vials if used at the point of administration. The RDTs evaluated in this study cost between USD 5–20 per test, however this is expected to be cheaper when ordered in bulk. It is notable that RDTs for a target pathogen are not able to detect all vaccines for that pathogen. For example, widely used *Plasmodium falciparum* malaria diagnostic RDTs based on detecting the pLDH and HRP-2 antigens would not detect adenovirus vector *P. falciparum* malaria vaccines. The ability of LFTs to detect vaccines also depends on vaccine formulation, and the ability of the surface antigens to interact molecularly with conjugated antibodies immobilised in the LFT devices. Therefore, the strategy is not universal and should not be generalised or translated to all vaccine products beyond the evidence presented in this manuscript. Often, vaccines use specialised formulation technologies to encapsulate (protect) the vaccine antigens for improved stability and/or better efficacy upon administration, and therefore, some genuine vaccine products may not test positive if tested using their respective LFTs using methods described in this paper. If samples fail testing with such LFT screening devices, reference assays will be needed to check this conclusion, but reference laboratories are not available in many countries.

Another limitation of this method is its inability to detect substandard vaccines, when genuine vaccine products have reduced potency, experience cold-chain excursions in supply chain or storage, or have out-of-specification impurities or related substances. These are protected against through supply chain and manufacturing QC/QA management and vaccine vial monitors on some vaccines, but they remain at risk of entering supply chains without detection.

Additional steps to understand the utility of RDTs in identifying falsified vaccines would include near-to-real life implementation trials and cost-effectiveness analysis for different contexts. Furthermore, specific RDTs could be developed for vaccines that do not yet have an RDT for detection, as well as developing quantitative RDTs that would facilitate the identification of the limit of detection, and whether a vaccine may be diluted or degraded.

## 5 Conclusions

This study provides proof-in-principle that existing commercially available RDTs may be repurposed for the detection of falsified vaccines. The results presented here demonstrate high accuracy using four different RDTs for the detection of seven different vaccines. The success of these experiments lays the foundation for further works to expand and validate the approach for diverse vaccines and RDTs and under different conditions, ideally involving a global inter-lab comparison. The research presented, and the suggested follow-up studies, will provide a foundation for the development of low-cost and effective devices that can be applied in supply chains for the authentication of vaccines worldwide. It is cautioned that findings from this study should not be generalised to other vaccines and related RDTs before individually validating for each vaccine-RDT pairs, and the present method may not accurately reassure the potency or overall quality of the vaccines tested, despite accurately identifying falsified products.

## Supplementary Material

Supplementary data for this article can be found online at https://doi.org/10.1016/j.vaccine.2024.01.019.

Supplementary data

## Figures and Tables

**Table 1:** Details of vaccines and RDTs used in the experiments.

Vaccine			Rapid diagnostic test		Method*
Name of vaccine	Details of vaccine		Name	Format	
Engerix B 20 μg/mL (SmithKline Beecham Ltd)	Recombinant protein vaccine containing the surface antigen of hepatitis B virus		Determine™ HBsAg (Abbott P/N 7D2947), detects the presence of hepatitis B surface antigen	Lateral flow test	50 μL sample onto the sample pad of the RDT and reading after 30 min
HBVAXPRO (Merck Sharp & Dohme UK Ltd)					
Prevenar-13 (Pfizer Ltd)	Conjugate polysaccharide vaccine for 13 serotypes of *Streptococcus pneumoniae*		BinaxNOW™ Streptococcus pneumoniae Antigen Card (Abbott 710100), detects the presence of streptococcus pneumoniae antigen		** Diluting the sample 1/10 in reagent A (proprietary reagent provided in the kit), pipetting 50 μL on the sample pad at the back of the RDT and reading after 15 min
Pneumovax-23 (Merck Sharp & Dohme UK Ltd)	Polysaccharide vaccine for 23 serotypes of *Streptococcus pneumoniae*				
MSP1 (University of Oxford)	Adenovirus vector vaccine with the *Plasmodium falciparum* MSP1 antigen		SD Bioline Rota/Adeno (Abbott 14FK20), detects the pesence of rotavirus or adenovirus antigen		100 μL sample onto the sample pad of the RDT and reading after 20 min
Nimenrix (Pfizer Ltd)	Conjugate polysaccharide vaccine for 4 serogroups of *Neisseria meningitidis*		Pastorex Meningitis kit (Biorad P/N 61607), detects antigens to *Neisseria meningitidis* groups A, B/*E. coli* K1, C, Y/W135, *Haemophilus*	Latex agglutination card	50 uL sample onto each circle on the card, one drop reagent added corresponding to the circle and mixed with a plastic mixing stick supplied in kit. The card was shaken horizontally on a rotator before reading at 10 min
Menitorix (GlaxoSmithKline UK)	Conjugate polysaccharide vaccine for *Neisseria meningitidis* serogroup C and *Haemophilus influenzae* type B.		*influenzae* type b, *Streptococcus pneumoniae* and group B *Streptococcus*,		

* according to manufacturer guidance with sample used = corresponding vaccine as prepared for vaccination, or vaccine surrogate.

** the RDT is designed for a swab, so modification from the manufacturer guidance was to dilute the vaccine 1/10 in reagent A (proprietary reagent provided in the kit) before loading on the rapid test.

**Table 2:** RDTs and vaccine vials tested in the initial assessment.

RDT	Vaccine	No. of vials tested	No. of replicate RDTs per vial	No. of replicate RDTs per vaccine type	No. of replicate RDTs per RDT type
Hepatitis B LFT	HBVAXPRO	10	3	30	60
	Engerix B	10	3	30	
*Streptococcus pneumoniae*	Prevenar 13	10	3	30	60
LFT	Pneumovax 23	10	3	30	
Rotavirus/Adenovirus LFT	MSP1	10	3	30	30
Latex agglutination	Nimenrix	10	1	10	20
	Menitorix	10	1	10	

**Table 3:** Sensitivity and specificity of four RDTs for detecting vaccines. 7 vaccines and 4 falsified vaccine surrogates were tested, each read by three readers independently.

	True positives	False positives	True negatives	False negatives	Sensitivity (95 %CI)	Specificity (95 %CI)
Hep B LFT	2	0	9	0	100 % (15.8–100)	100 % (63.1–100)
*Streptococcus pneumoniae* LFT	2	0	9	0	100 % (15.8–100)	100 % (66.4–100)
Rotavirus/Adenovirus LFT	1	1*	9	0	100 % (2.5–100)	90.0 % (47.3–99.7)*
Latex agglutination	2	0	9	0	100 % (15.8–100)	100 % (59.1–100)

* A weak false positive for the Rota/Adeno LFT was seen with 5% Glucose.

## Data Availability

Data will be made available on request.
