# Supplementary figures and images for: Repurposing rapid diagnostic tests to detect falsified vaccines in supply chains
Source: Vaccine. Author manuscript; Available in PMC 2025 Aug 27. (PMC7618033; doi:10.1016/j.vaccine.2024.01.019)

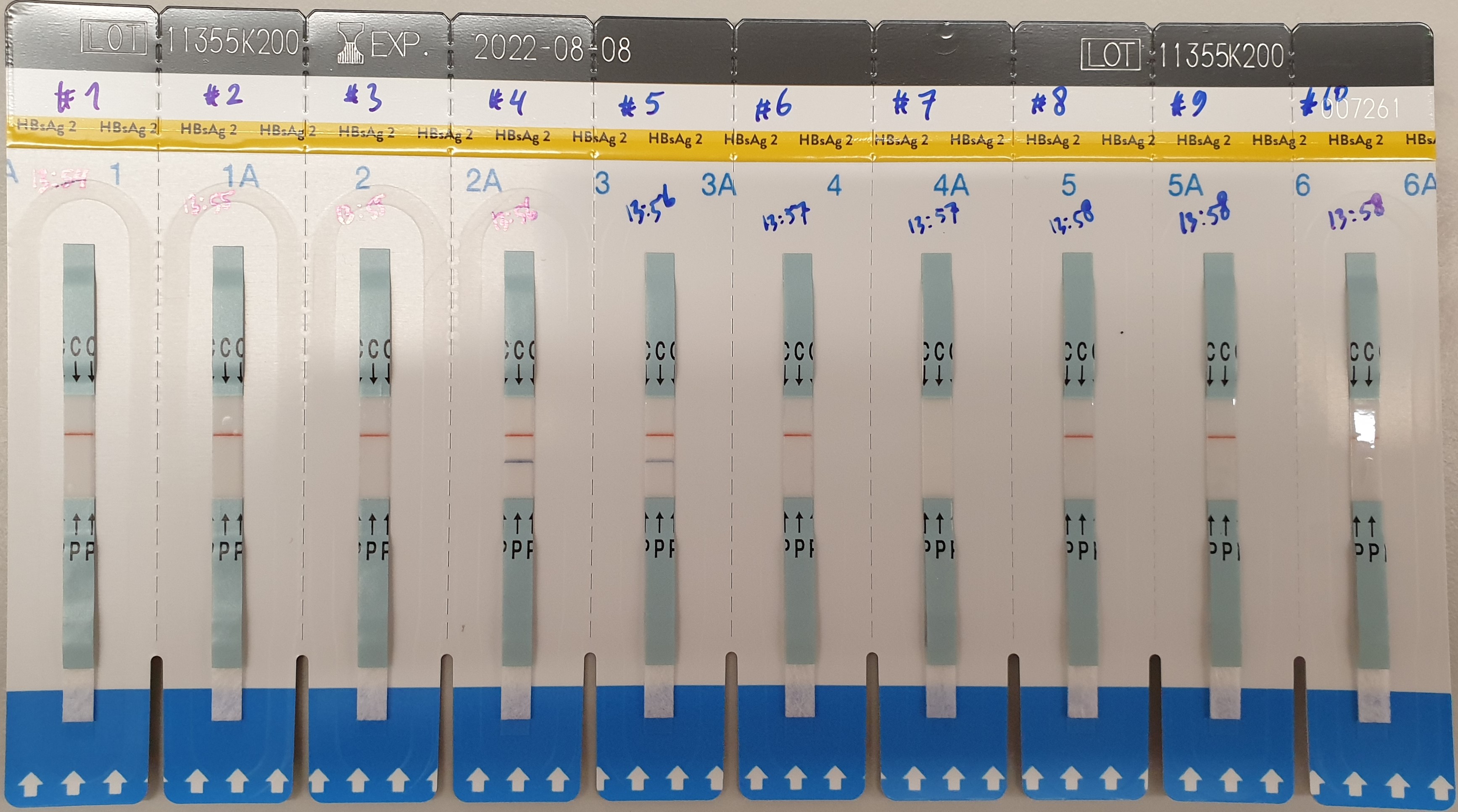

Supplement: Supplementary data [file EMS207833-supplement-Supplementary_data.zip › Blinded study/Hep B LFT_Samples 1-10.jpg]

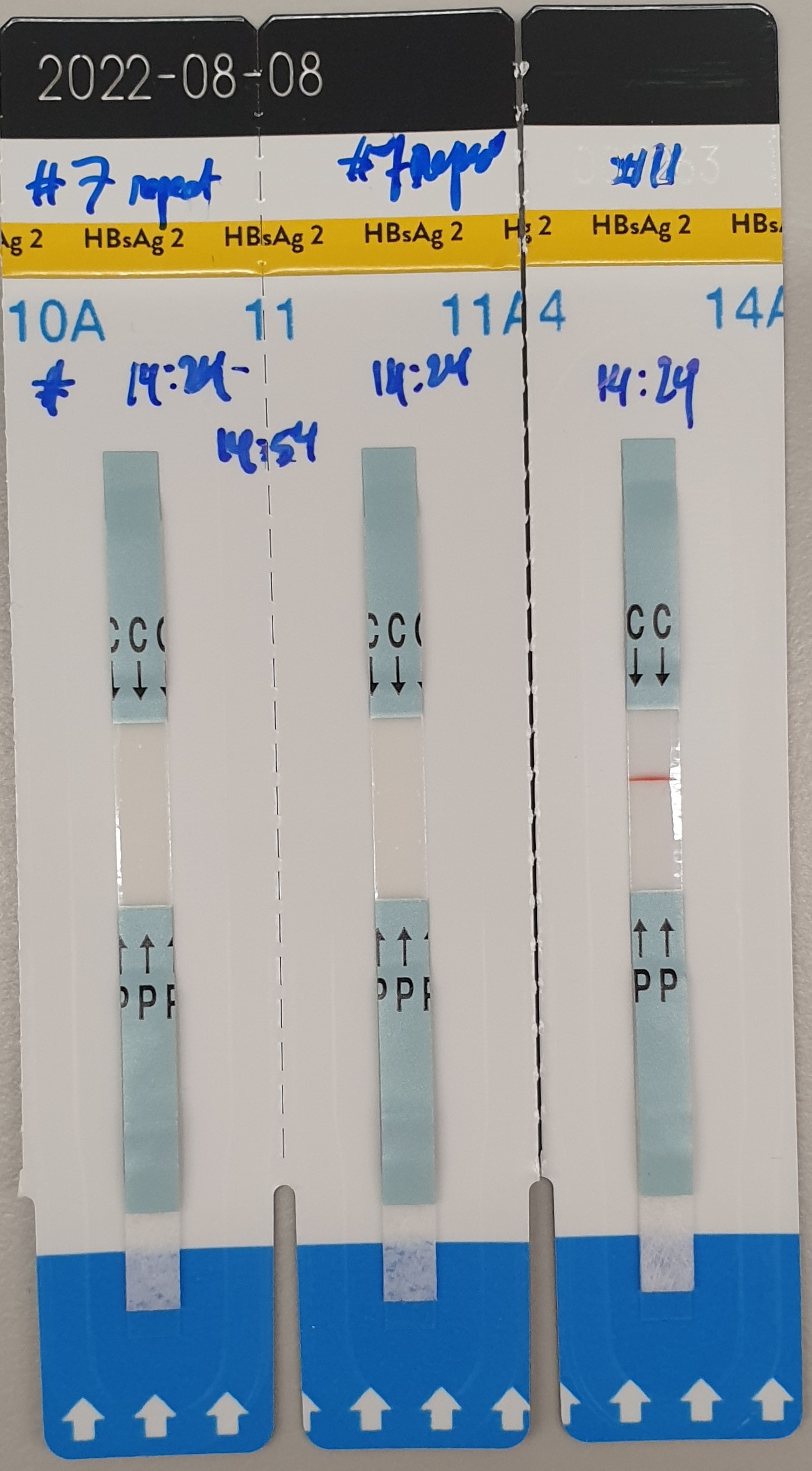

Supplement: Supplementary data [file EMS207833-supplement-Supplementary_data.zip › Blinded study/Hep B LFT_Samples 7R and 11.jpg]

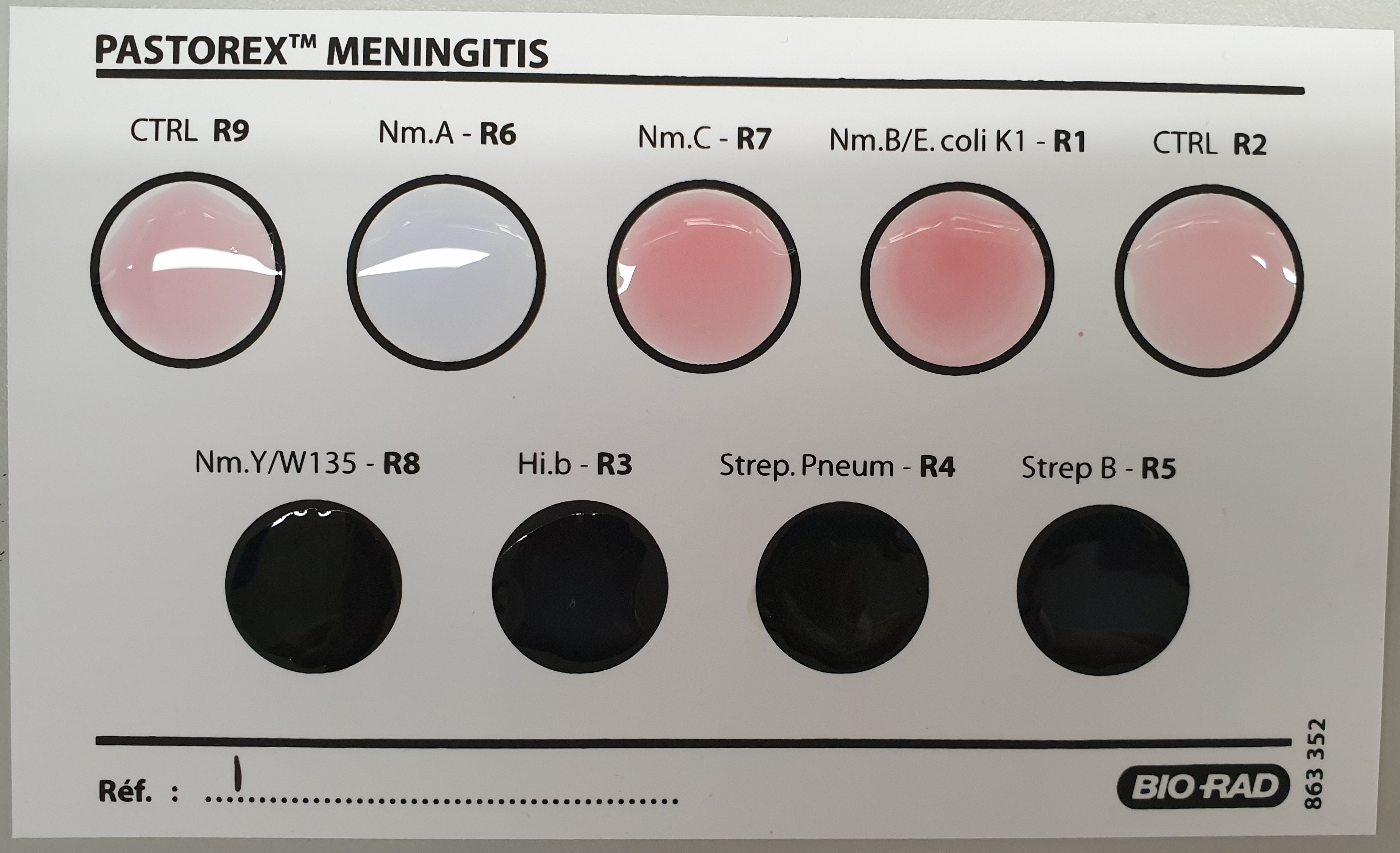

Supplement: Supplementary data [file EMS207833-supplement-Supplementary_data.zip › Blinded study/Latex agglutination kit_Sample 1.jpg]

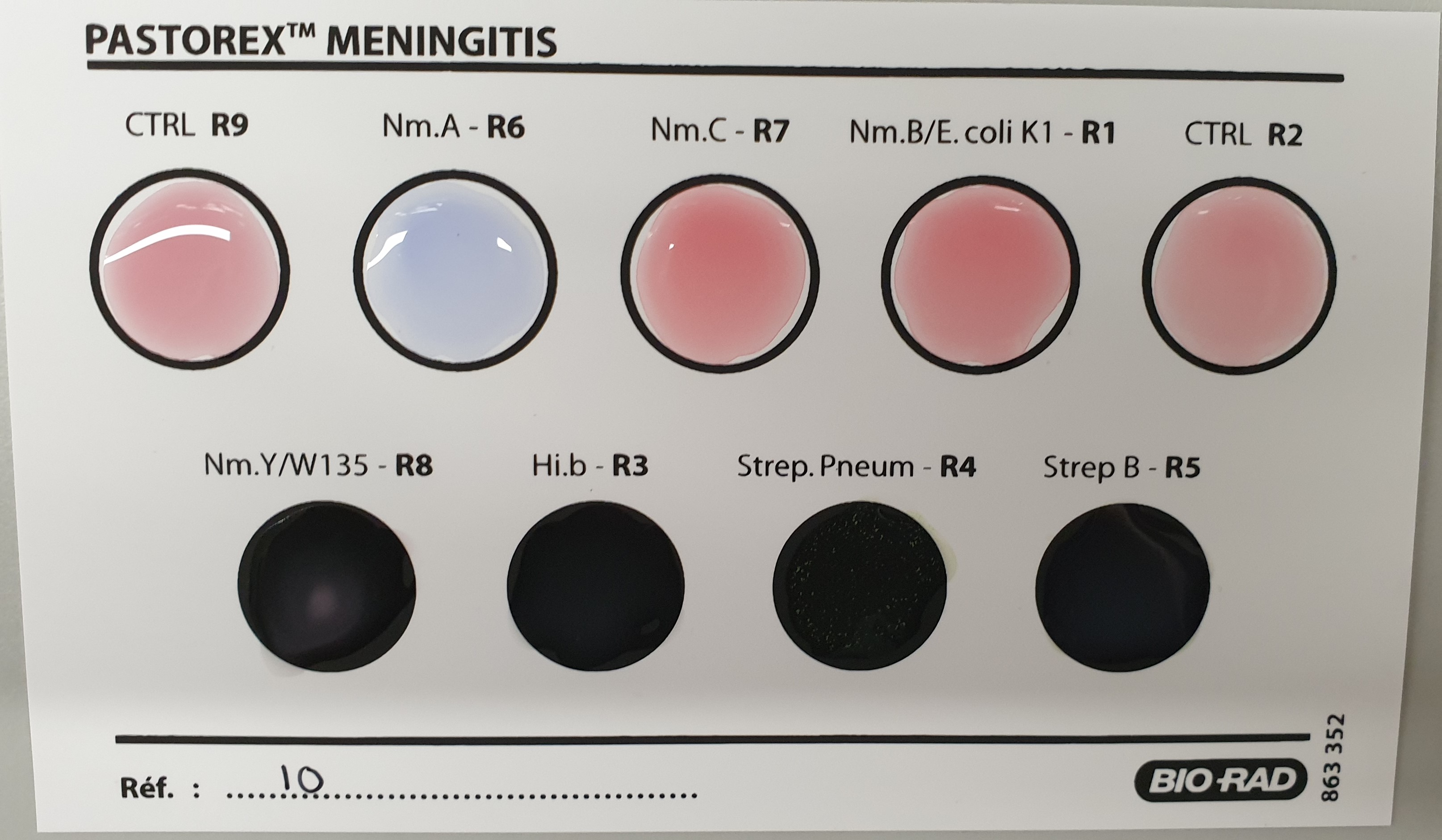

Supplement: Supplementary data [file EMS207833-supplement-Supplementary_data.zip › Blinded study/Latex agglutination kit_Sample 10.jpg]

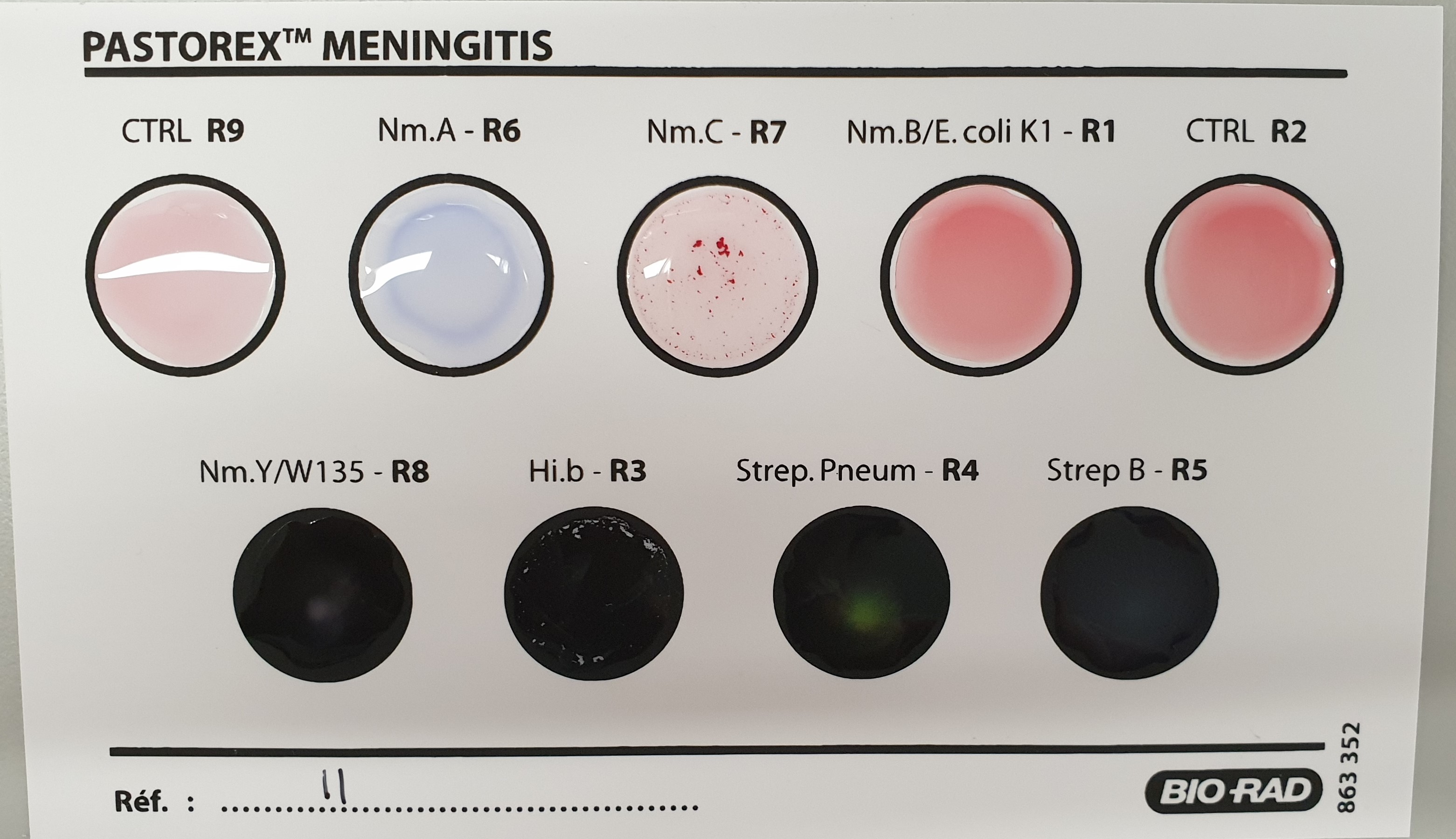

Supplement: Supplementary data [file EMS207833-supplement-Supplementary_data.zip › Blinded study/Latex agglutination kit_Sample 11.jpg]

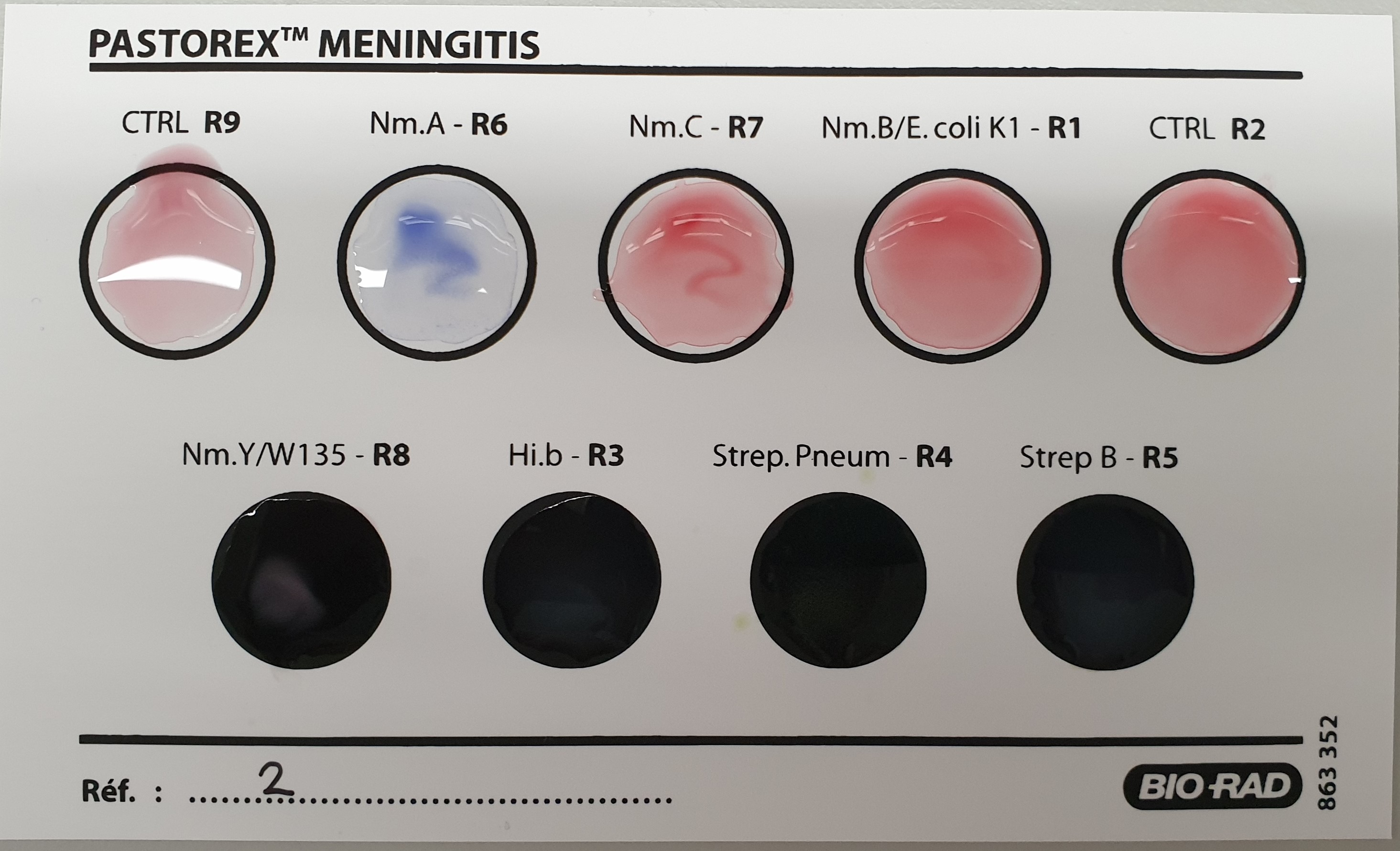

Supplement: Supplementary data [file EMS207833-supplement-Supplementary_data.zip › Blinded study/Latex agglutination kit_Sample 2.jpg]

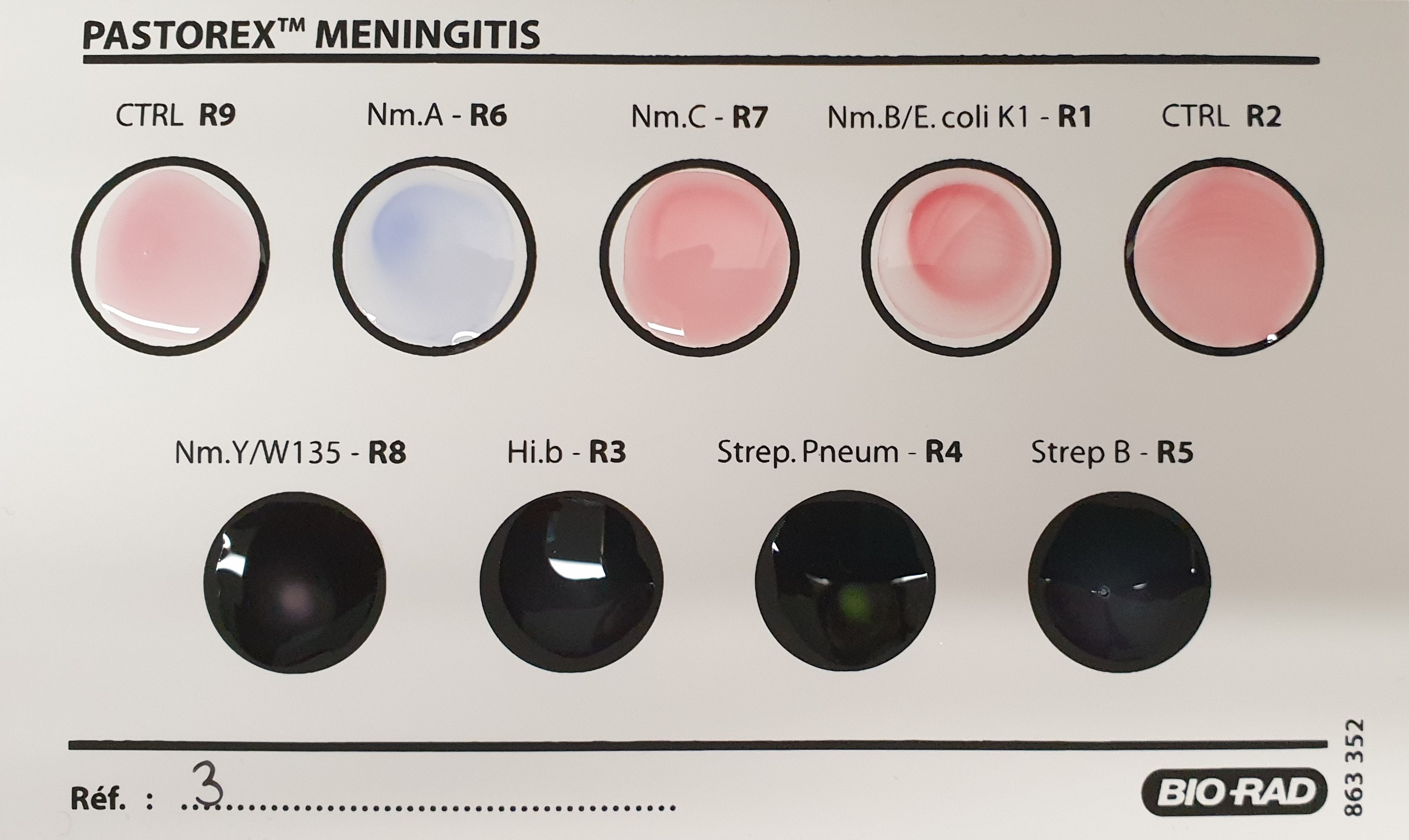

Supplement: Supplementary data [file EMS207833-supplement-Supplementary_data.zip › Blinded study/Latex agglutination kit_Sample 3.jpg]

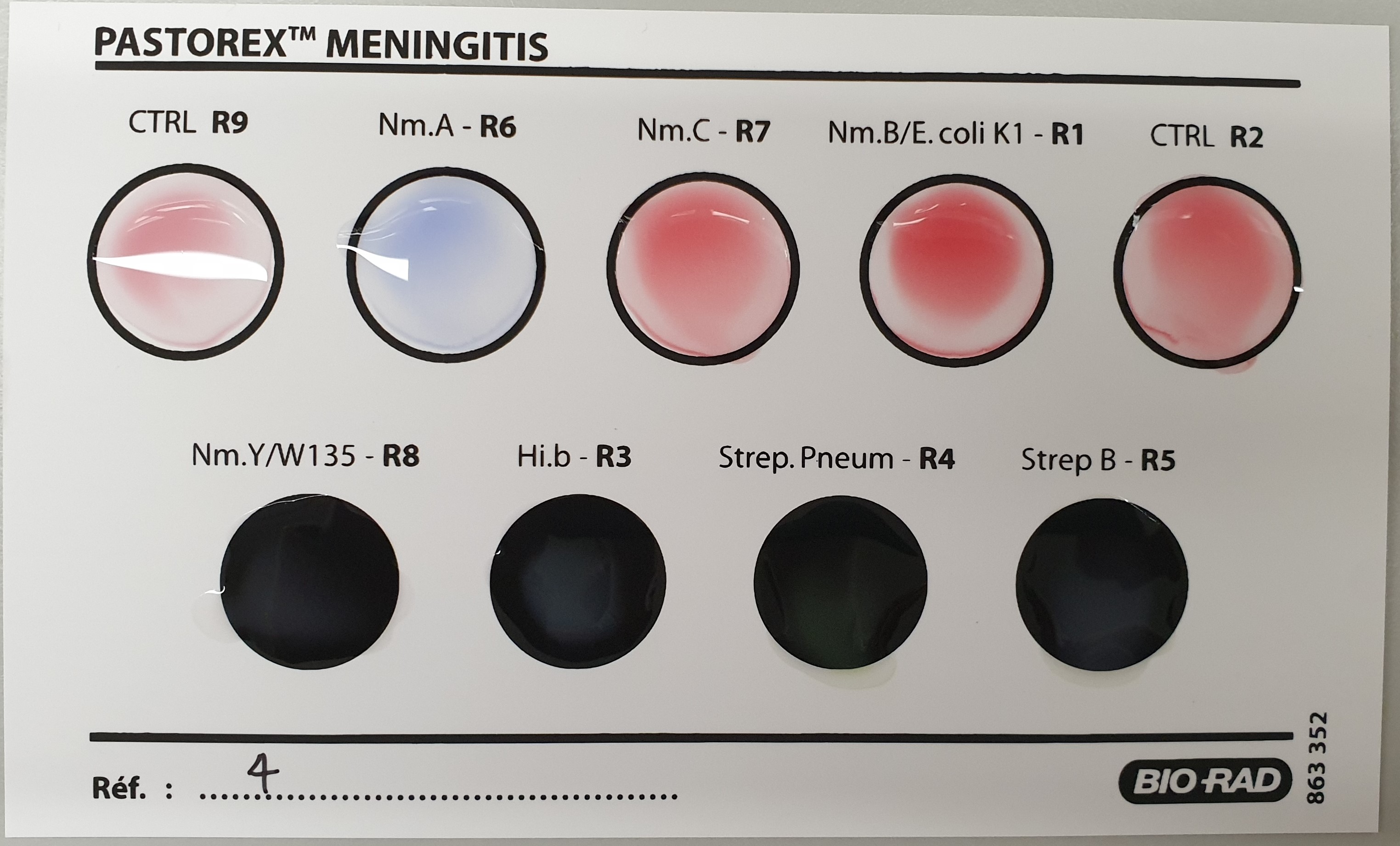

Supplement: Supplementary data [file EMS207833-supplement-Supplementary_data.zip › Blinded study/Latex agglutination kit_Sample 4.jpg]

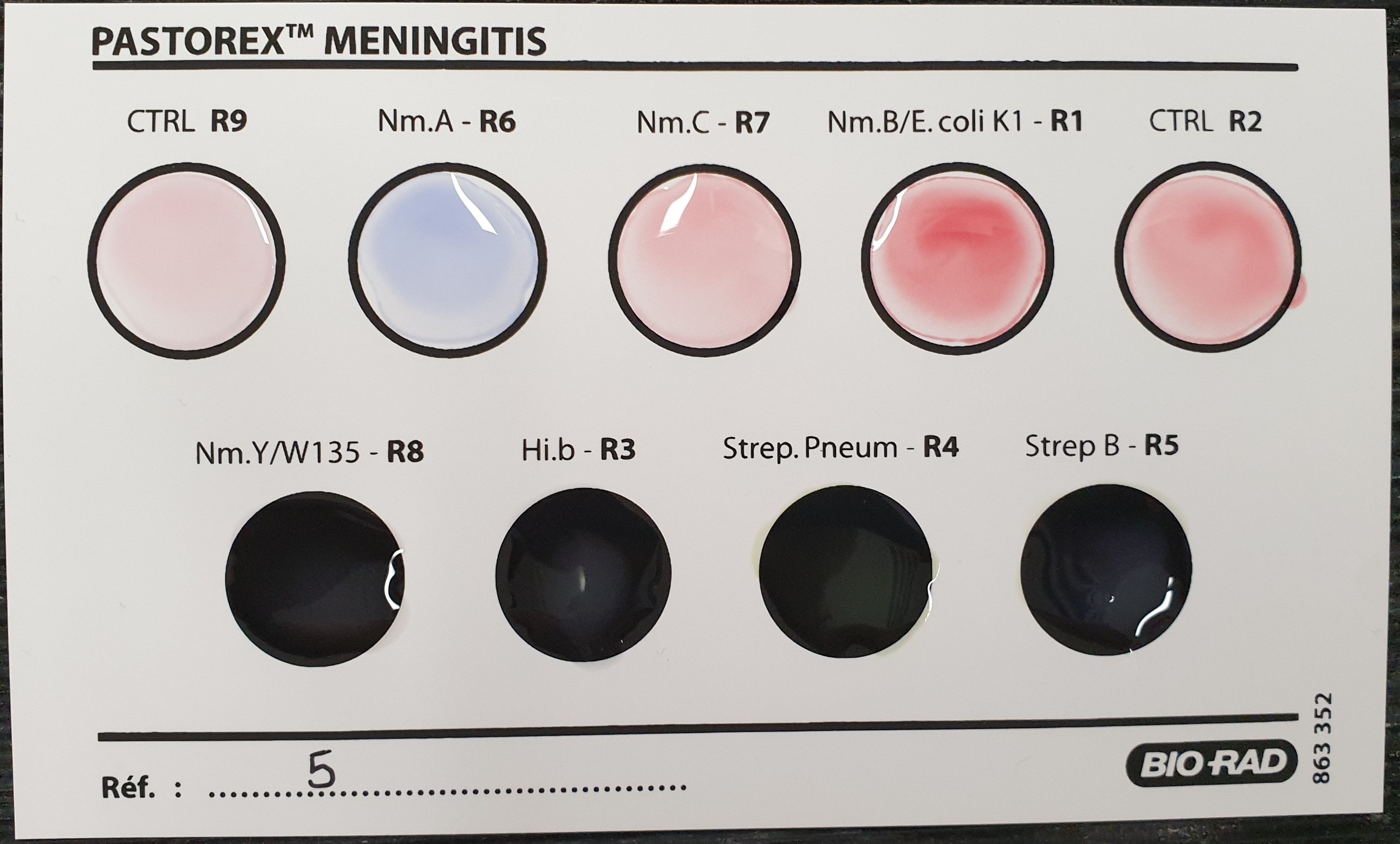

Supplement: Supplementary data [file EMS207833-supplement-Supplementary_data.zip › Blinded study/Latex agglutination kit_Sample 5.jpg]

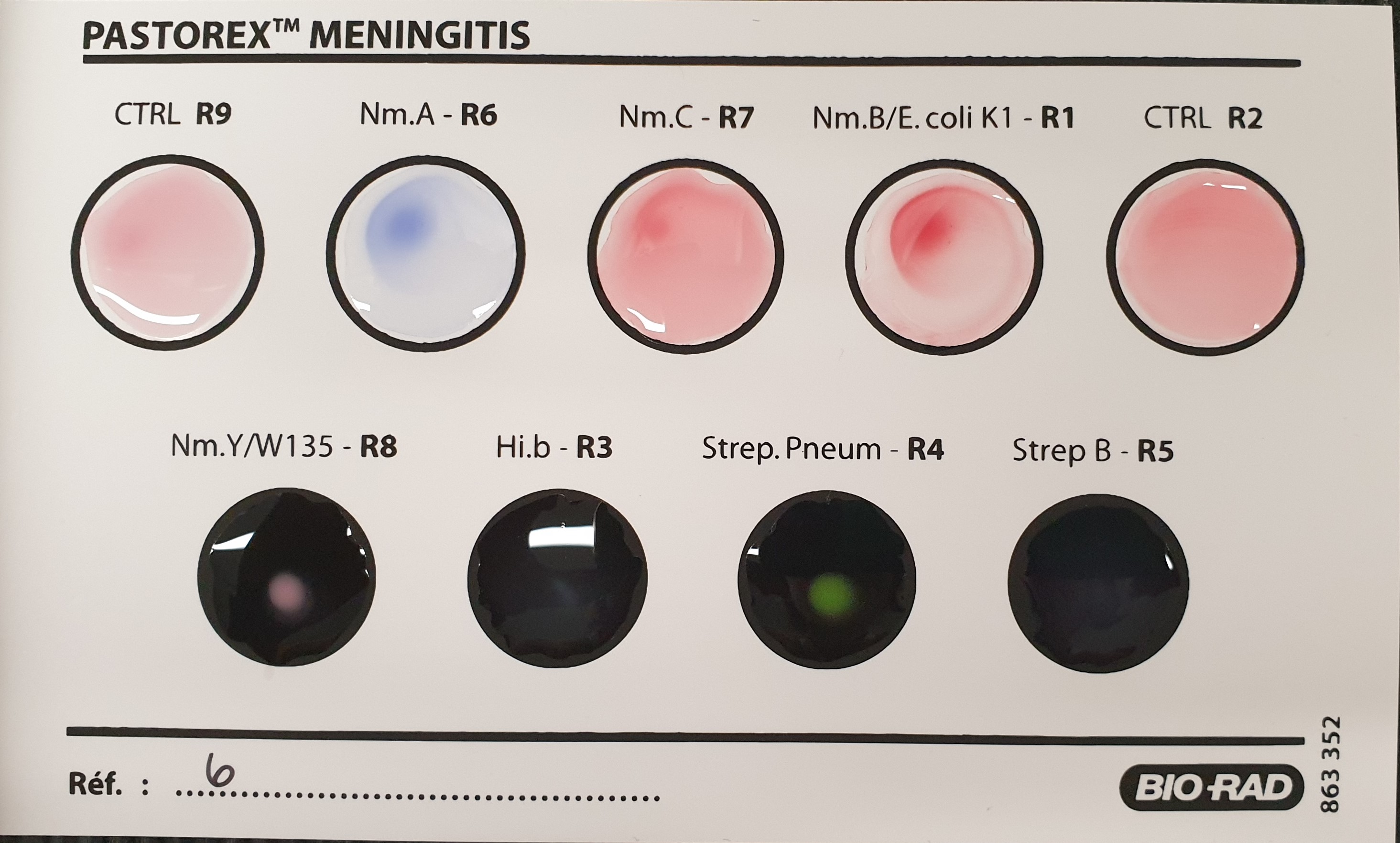

Supplement: Supplementary data [file EMS207833-supplement-Supplementary_data.zip › Blinded study/Latex agglutination kit_Sample 6.jpg]

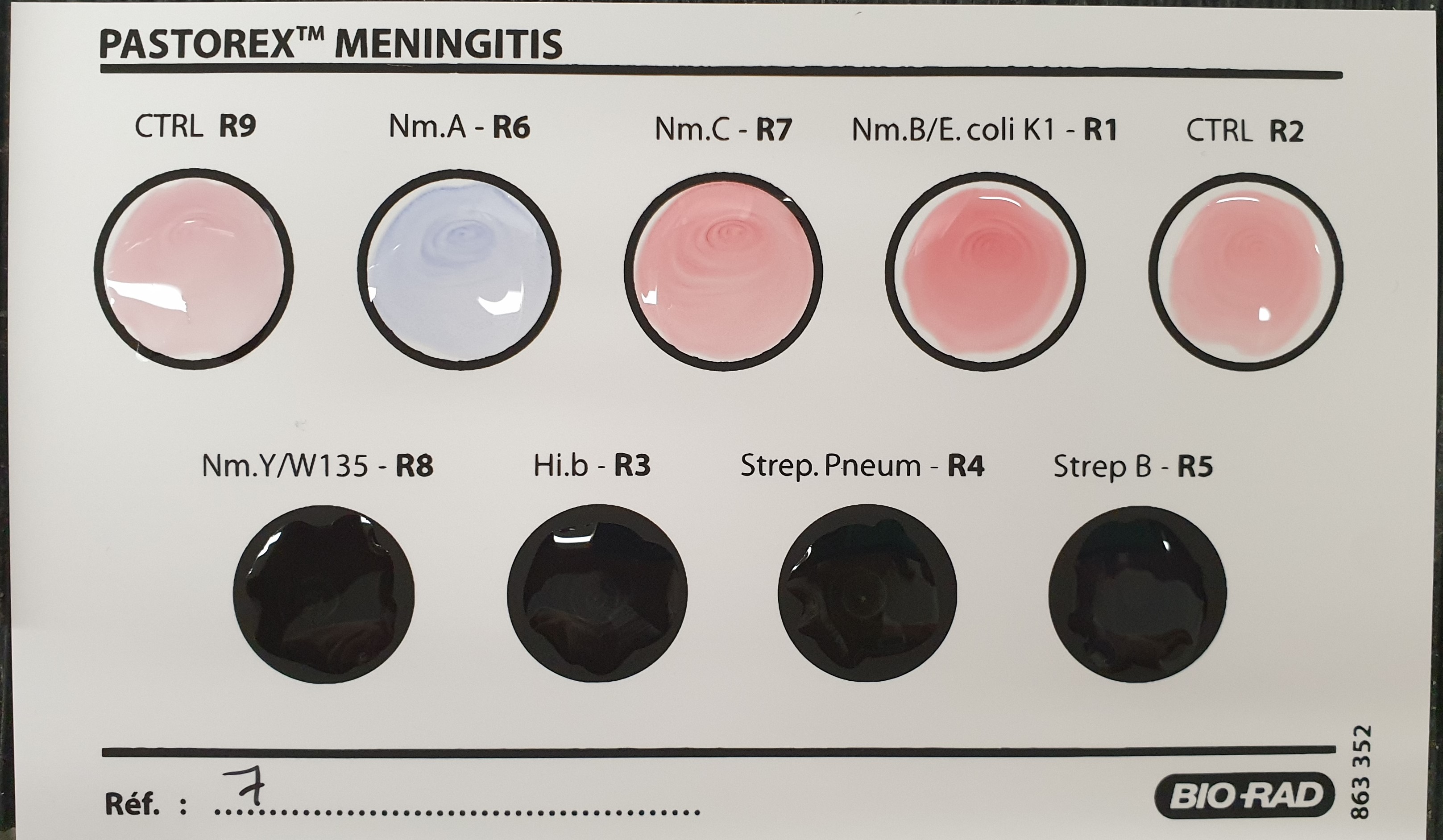

Supplement: Supplementary data [file EMS207833-supplement-Supplementary_data.zip › Blinded study/Latex agglutination kit_Sample 7.jpg]

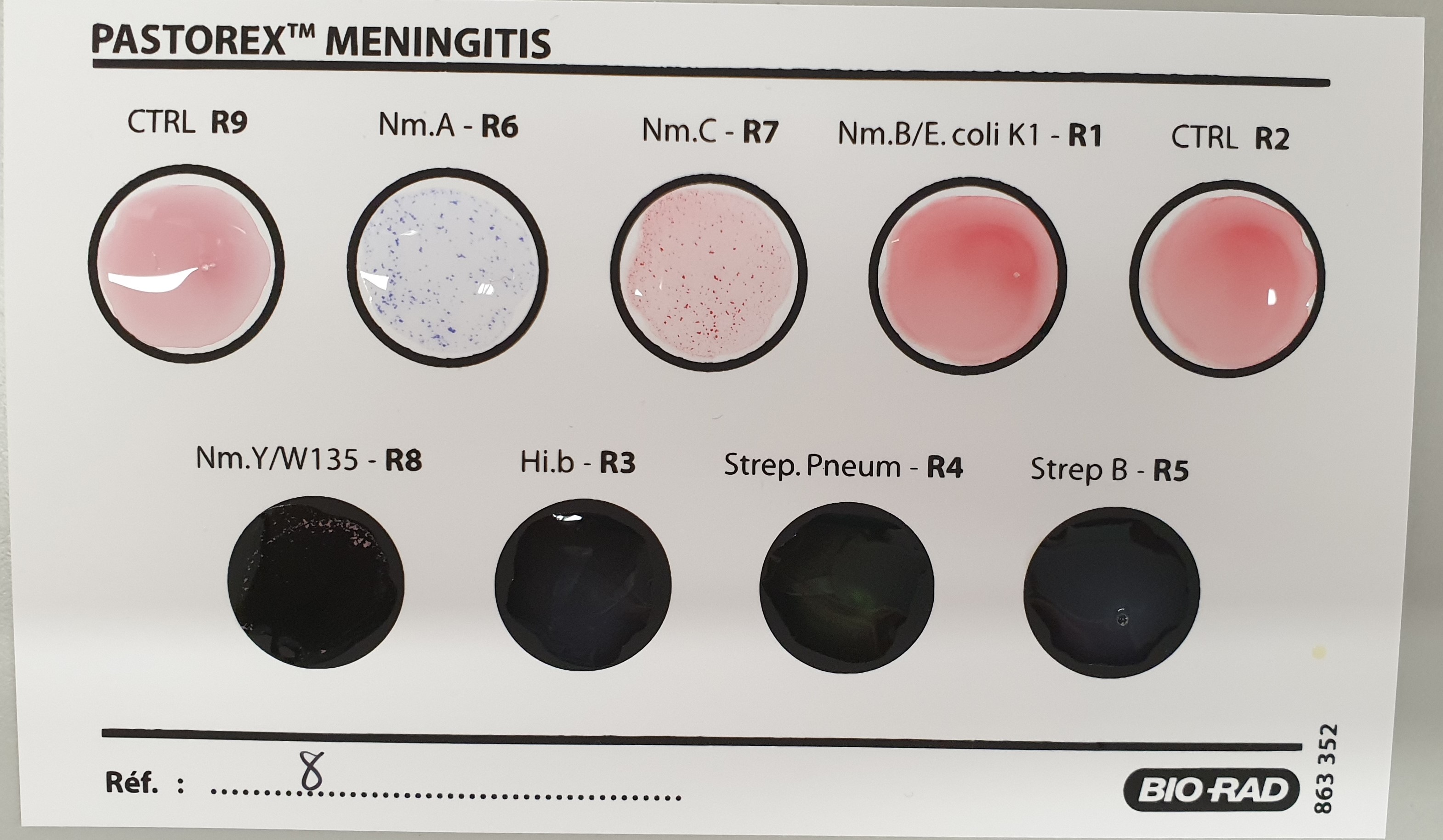

Supplement: Supplementary data [file EMS207833-supplement-Supplementary_data.zip › Blinded study/Latex agglutination kit_Sample 8.jpg]

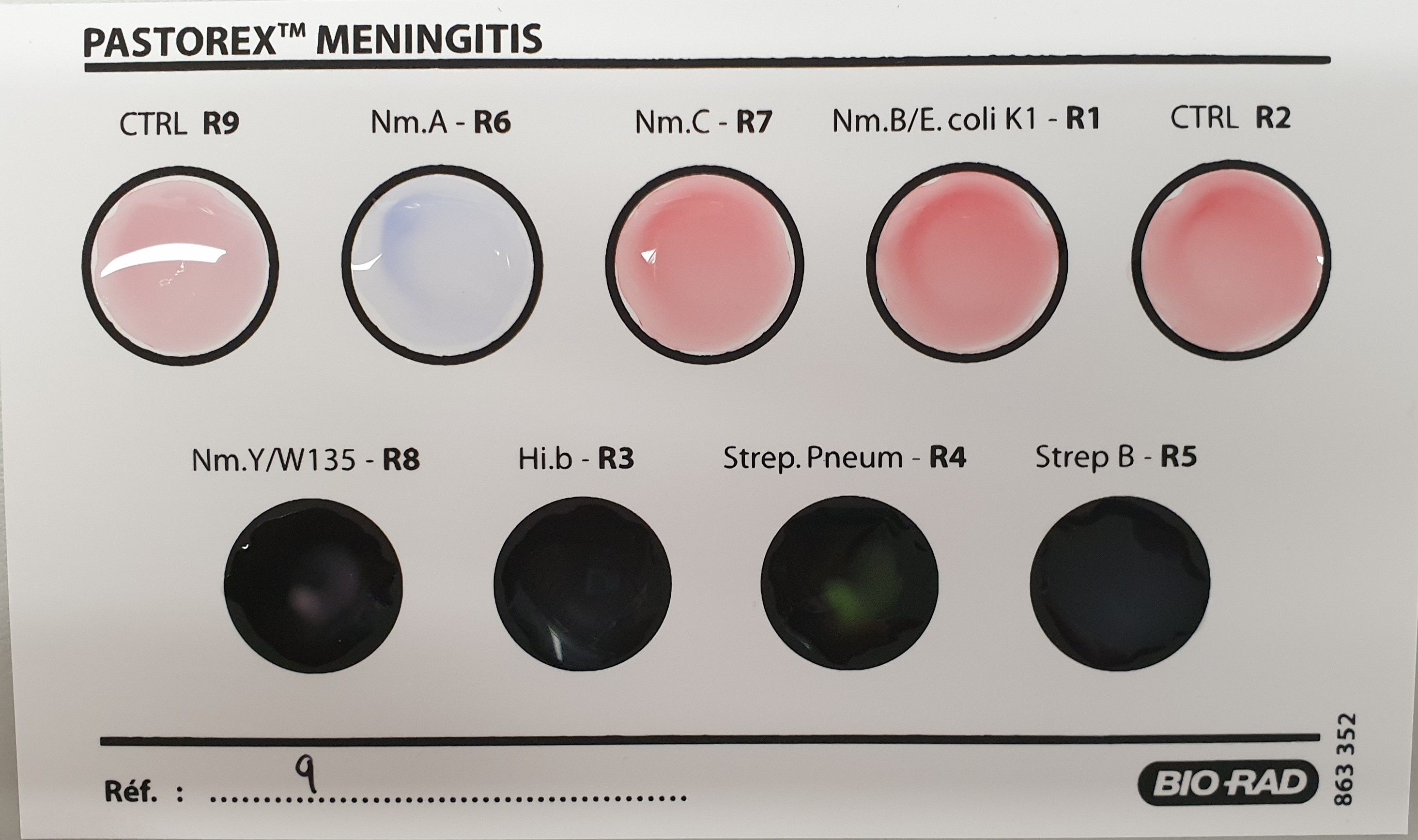

Supplement: Supplementary data [file EMS207833-supplement-Supplementary_data.zip › Blinded study/Latex agglutination kit_Sample 9.jpg]

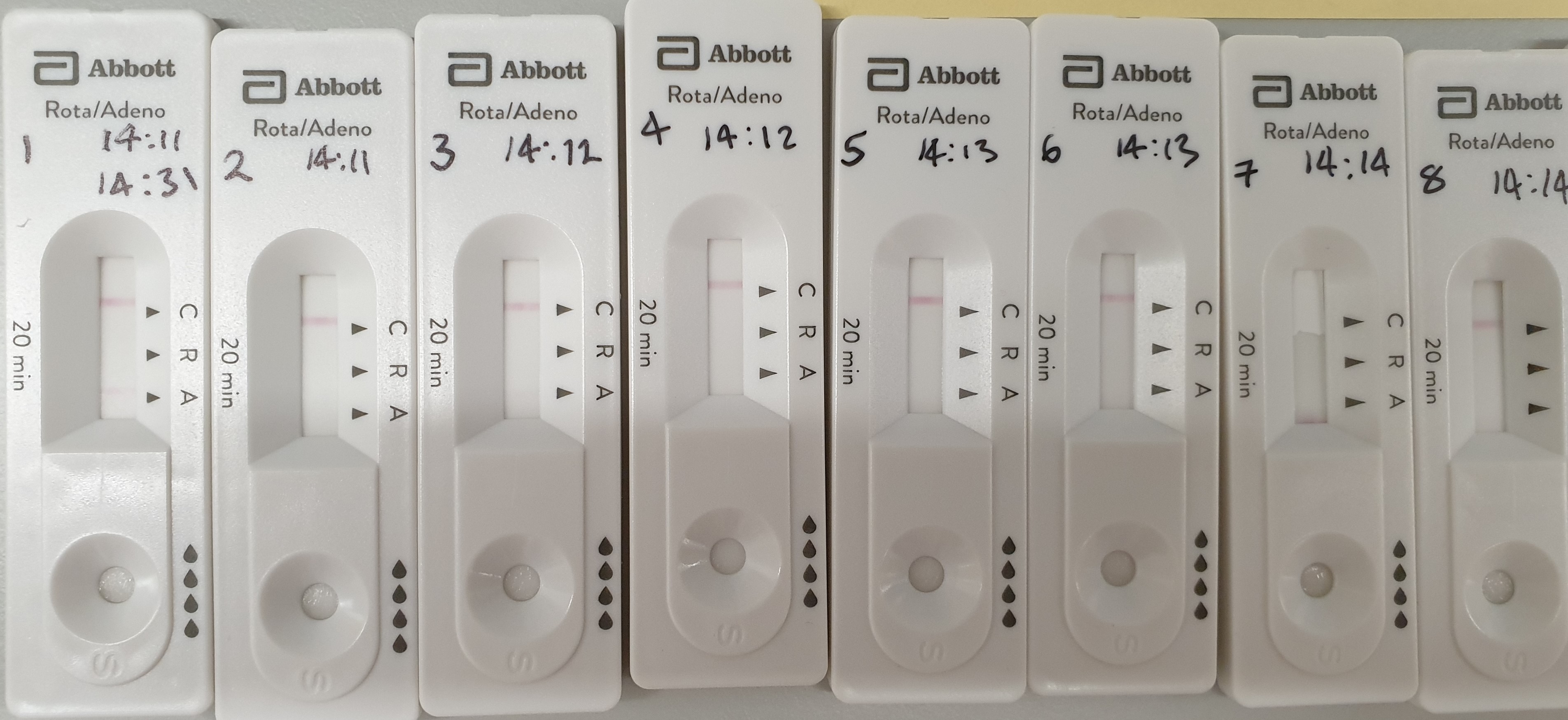

Supplement: Supplementary data [file EMS207833-supplement-Supplementary_data.zip › Blinded study/Rota-Adeno RDT_Samples 1-8.jpg]

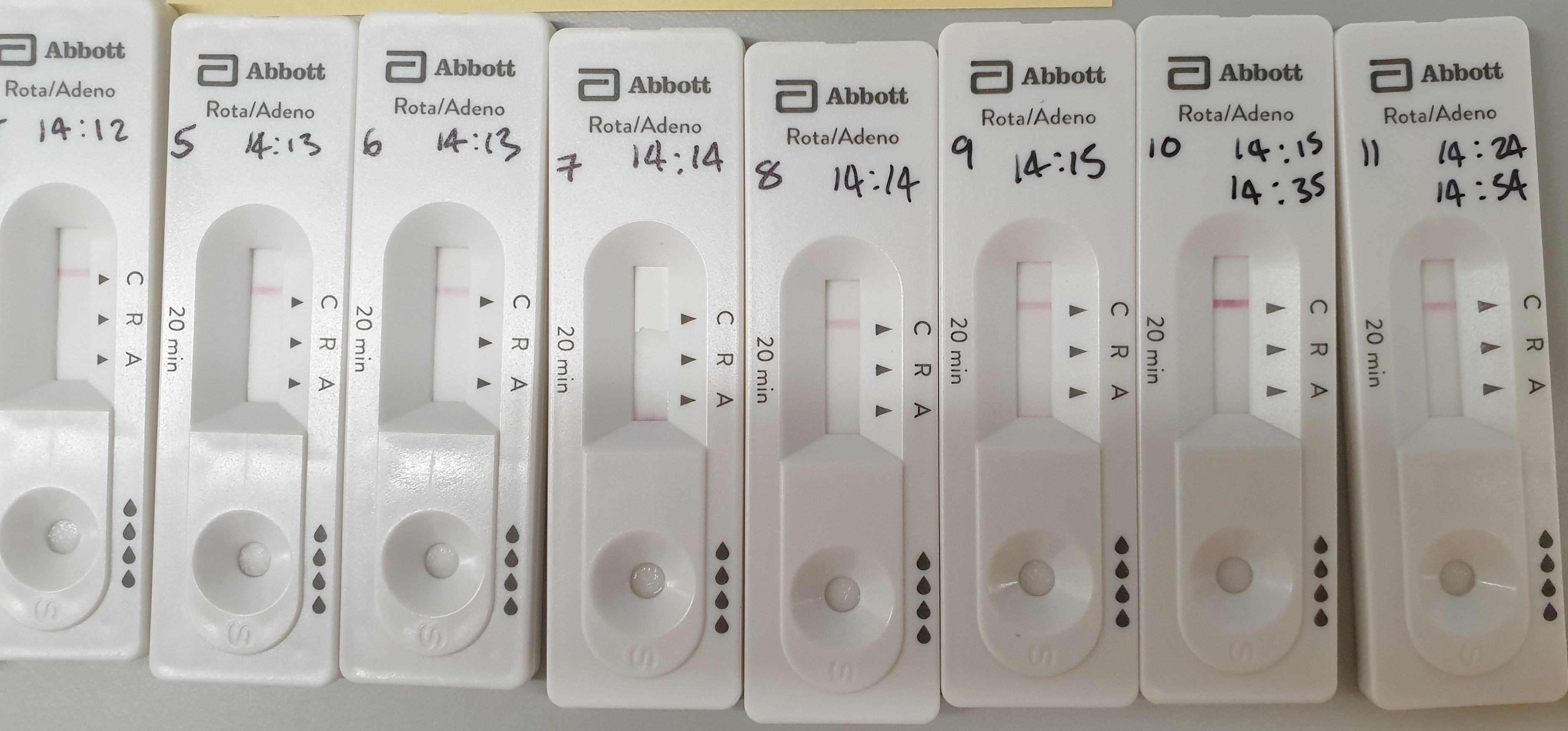

Supplement: Supplementary data [file EMS207833-supplement-Supplementary_data.zip › Blinded study/Rota-Adeno RDT_Samples 4-11.jpg]

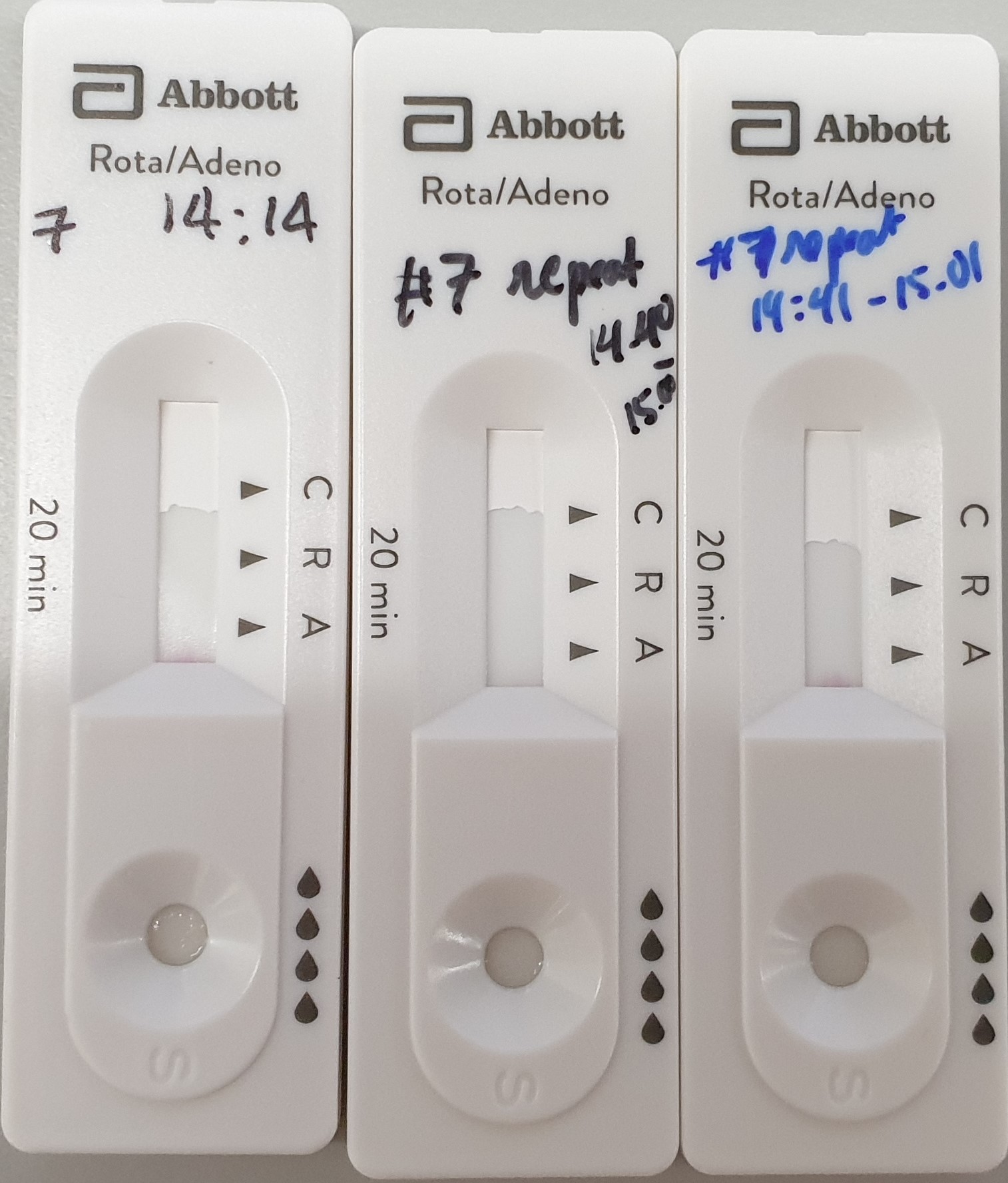

Supplement: Supplementary data [file EMS207833-supplement-Supplementary_data.zip › Blinded study/Rota-Adeno RDT_Samples 7 R1-3.jpg]

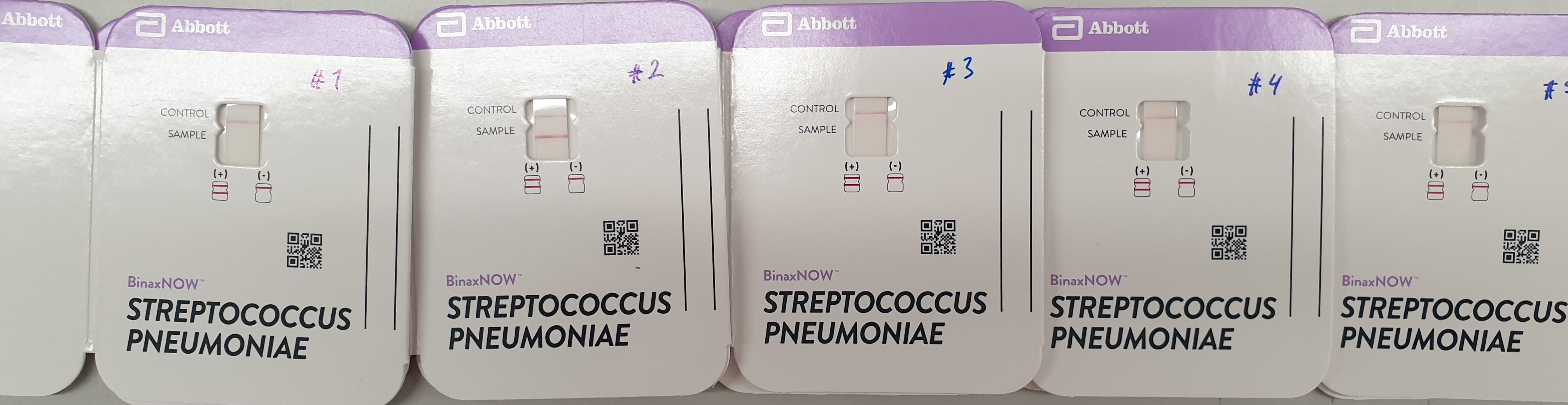

Supplement: Supplementary data [file EMS207833-supplement-Supplementary_data.zip › Blinded study/Strep pneumo LFT_Sample1-5.jpg]

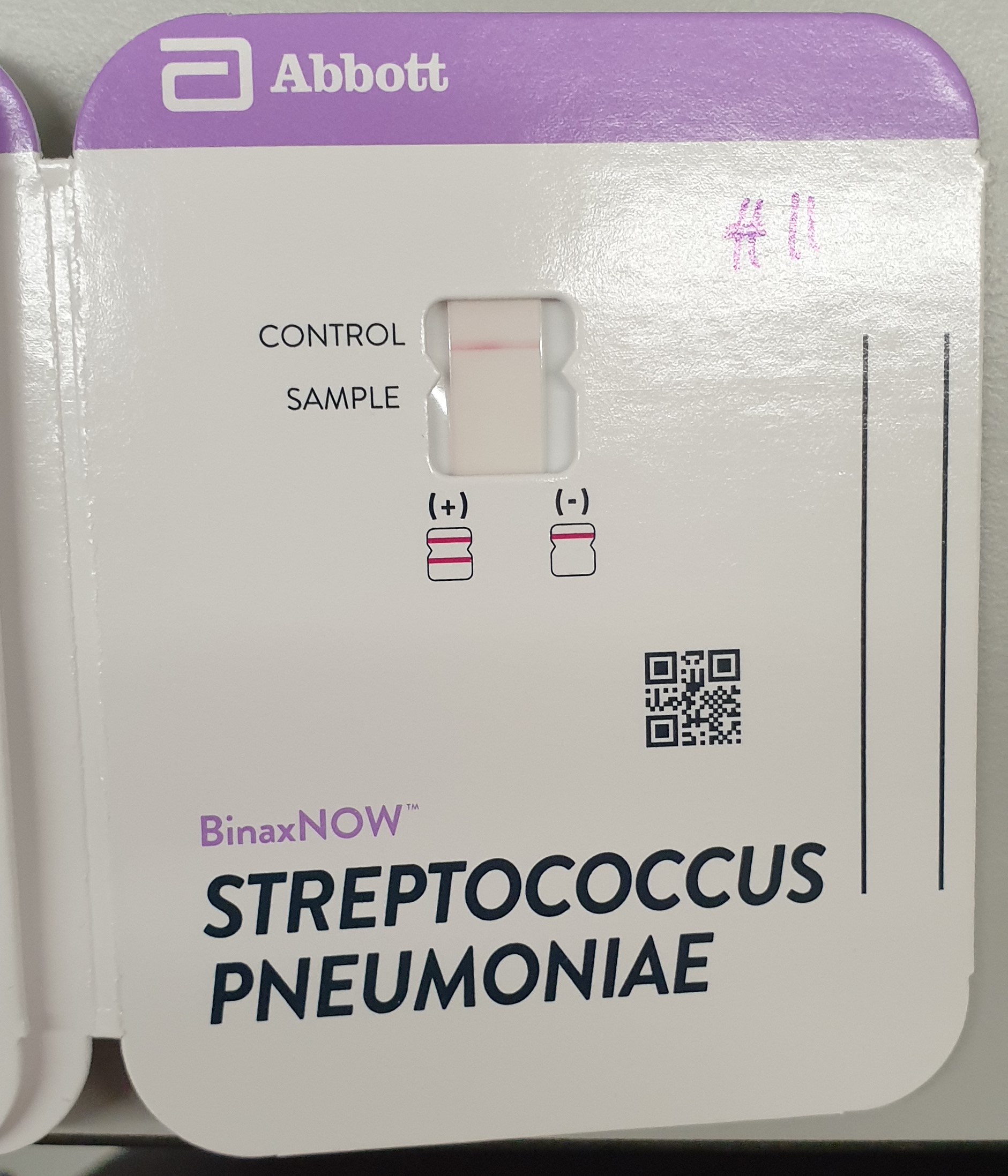

Supplement: Supplementary data [file EMS207833-supplement-Supplementary_data.zip › Blinded study/Strep pneumo LFT_Samples 11.jpg]

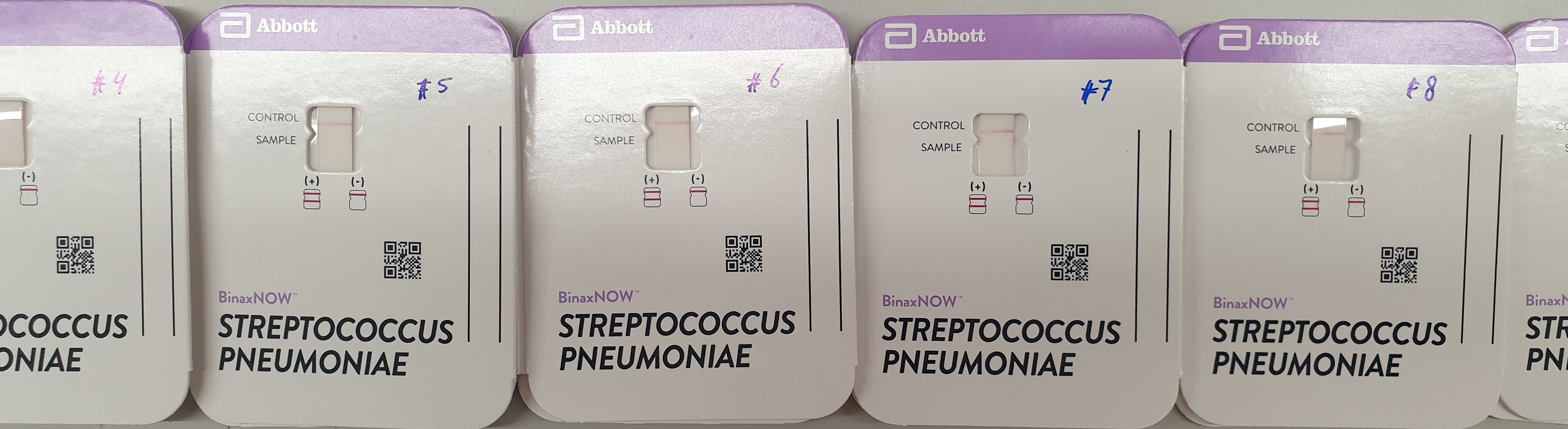

Supplement: Supplementary data [file EMS207833-supplement-Supplementary_data.zip › Blinded study/Strep pneumo LFT_Samples 4-8.jpg]

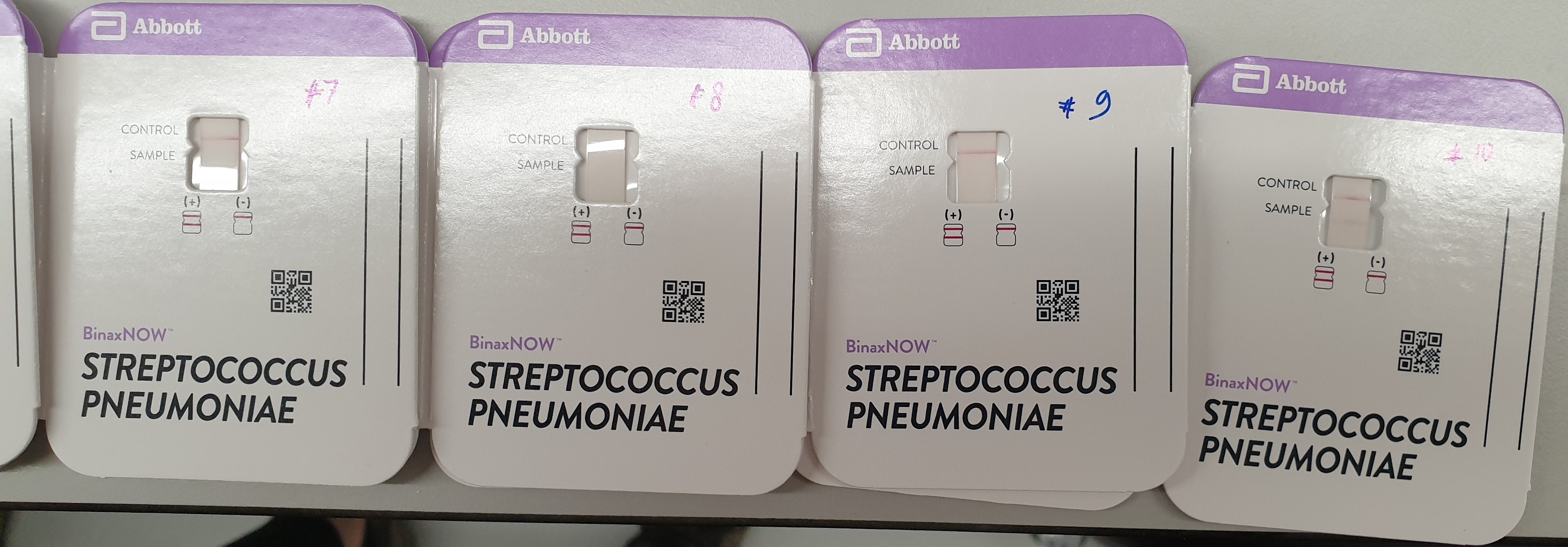

Supplement: Supplementary data [file EMS207833-supplement-Supplementary_data.zip › Blinded study/Strep pneumo LFT_Samples 7-10.jpg]

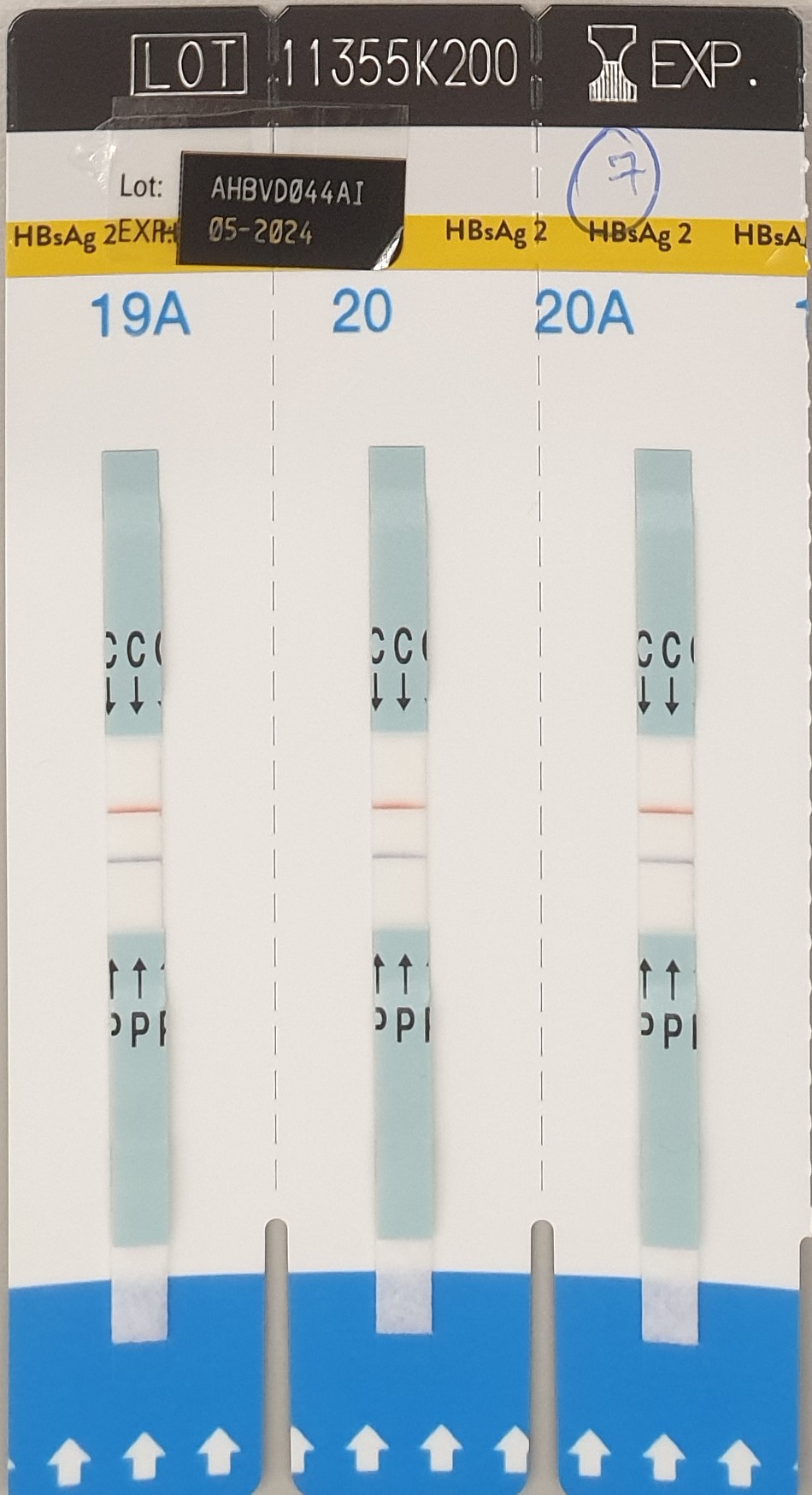

Supplement: Supplementary data [file EMS207833-supplement-Supplementary_data.zip › Initial assessment/Engerix B/Engerix B_Batch 1_Vial 1.jpg]

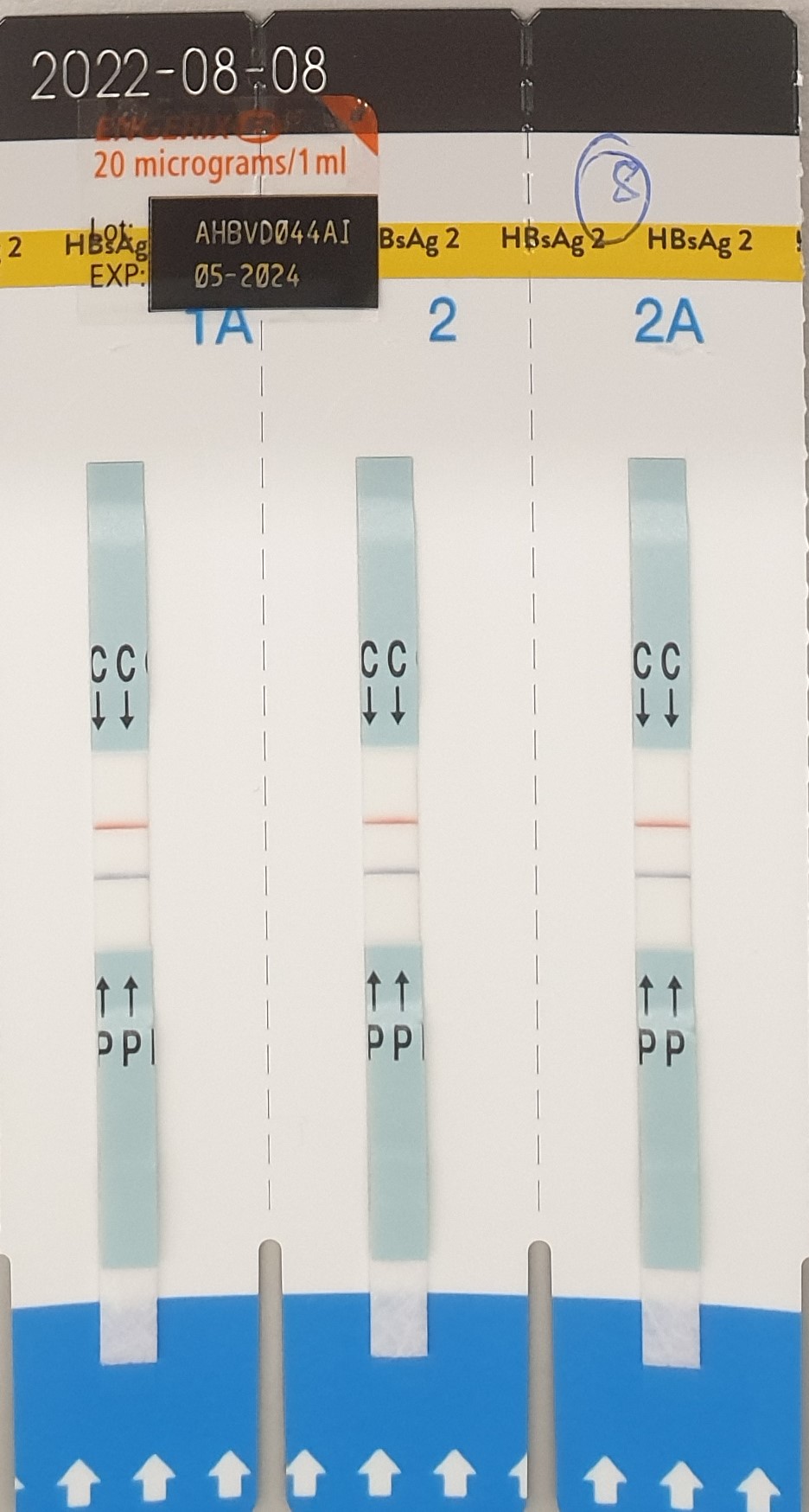

Supplement: Supplementary data [file EMS207833-supplement-Supplementary_data.zip › Initial assessment/Engerix B/Engerix B_Batch 1_Vial 2.jpg]

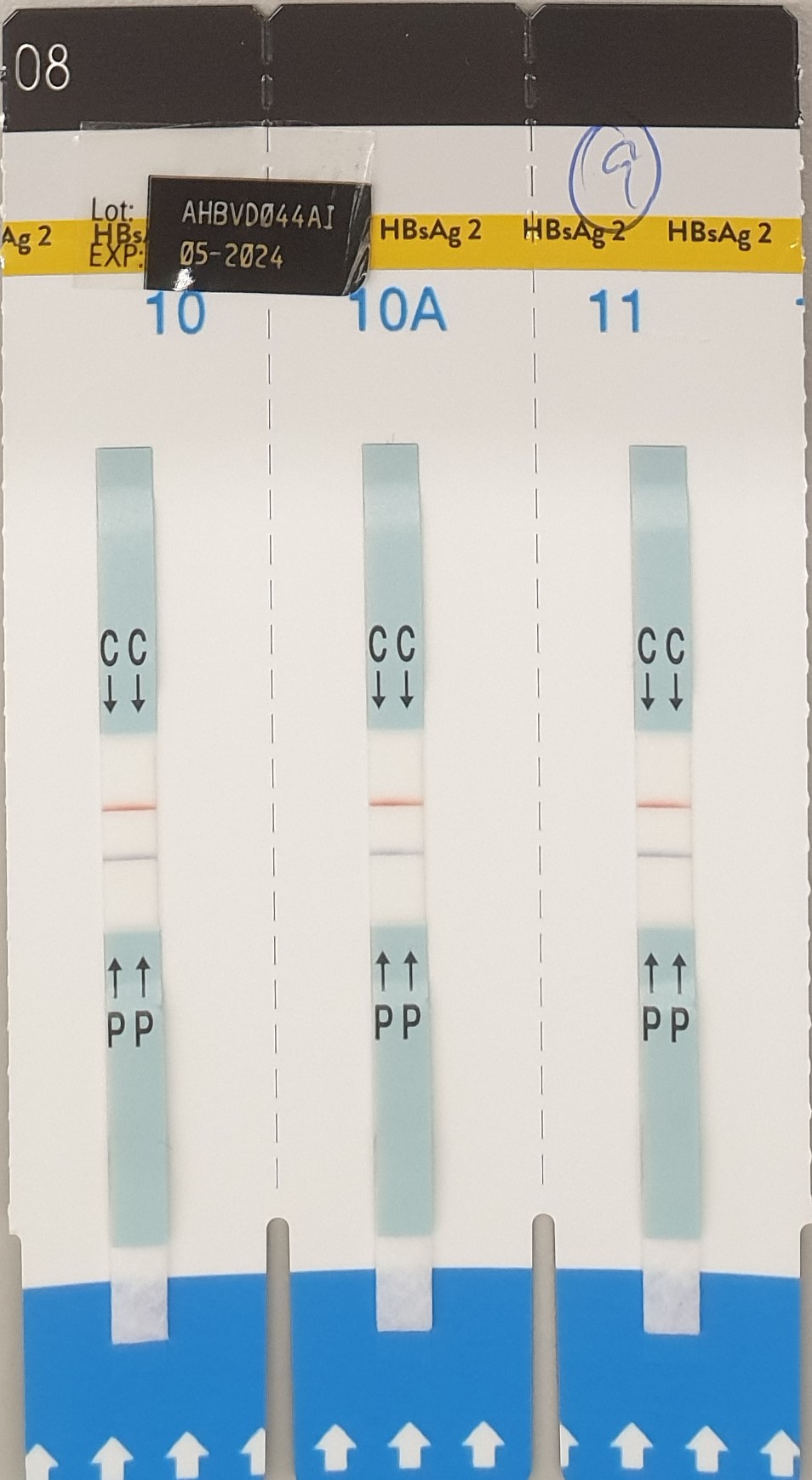

Supplement: Supplementary data [file EMS207833-supplement-Supplementary_data.zip › Initial assessment/Engerix B/Engerix B_Batch 1_Vial 3.jpg]

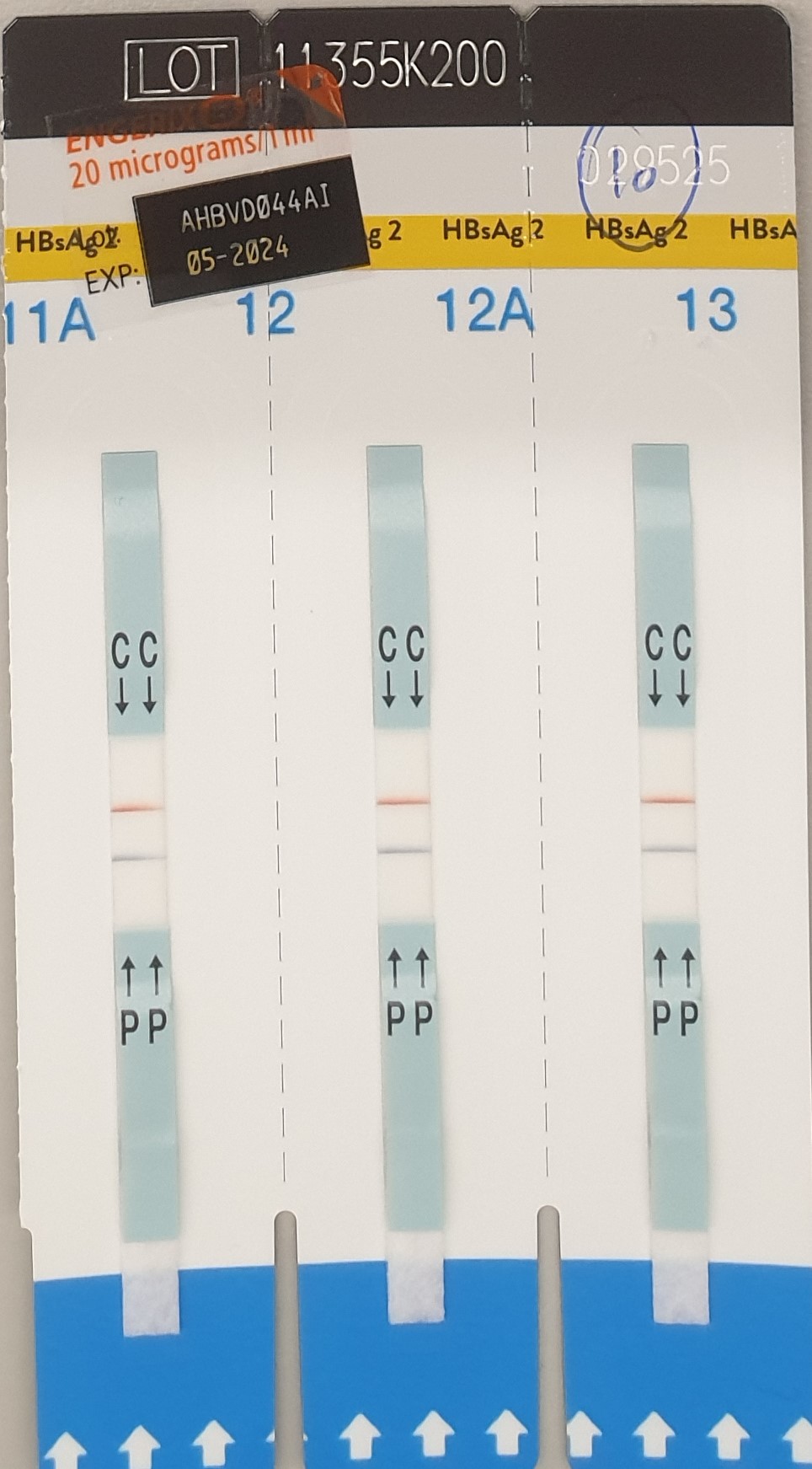

Supplement: Supplementary data [file EMS207833-supplement-Supplementary_data.zip › Initial assessment/Engerix B/Engerix B_Batch 1_Vial 4.jpg]

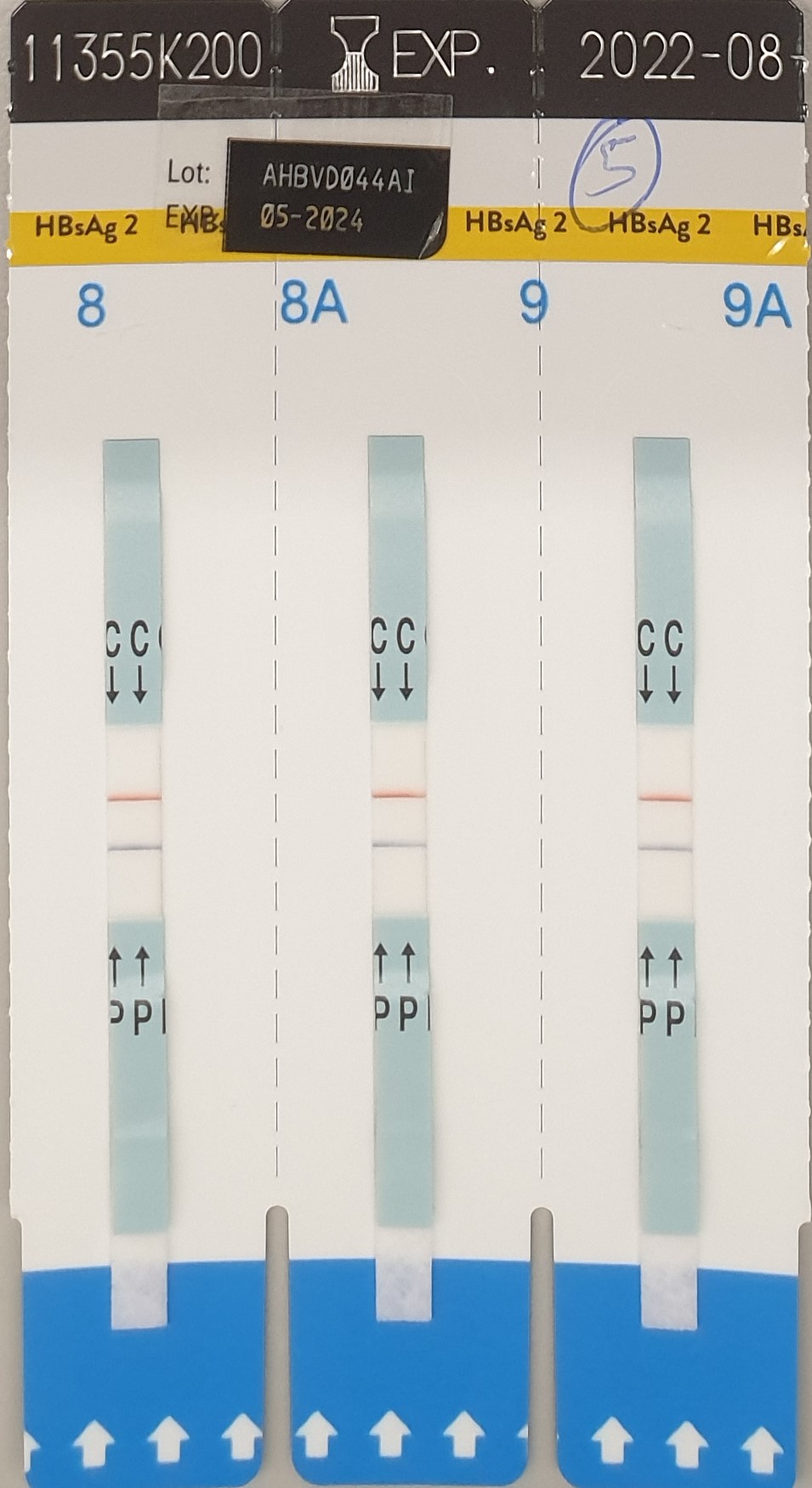

Supplement: Supplementary data [file EMS207833-supplement-Supplementary_data.zip › Initial assessment/Engerix B/Engerix B_Batch 1_Vial 5.jpg]

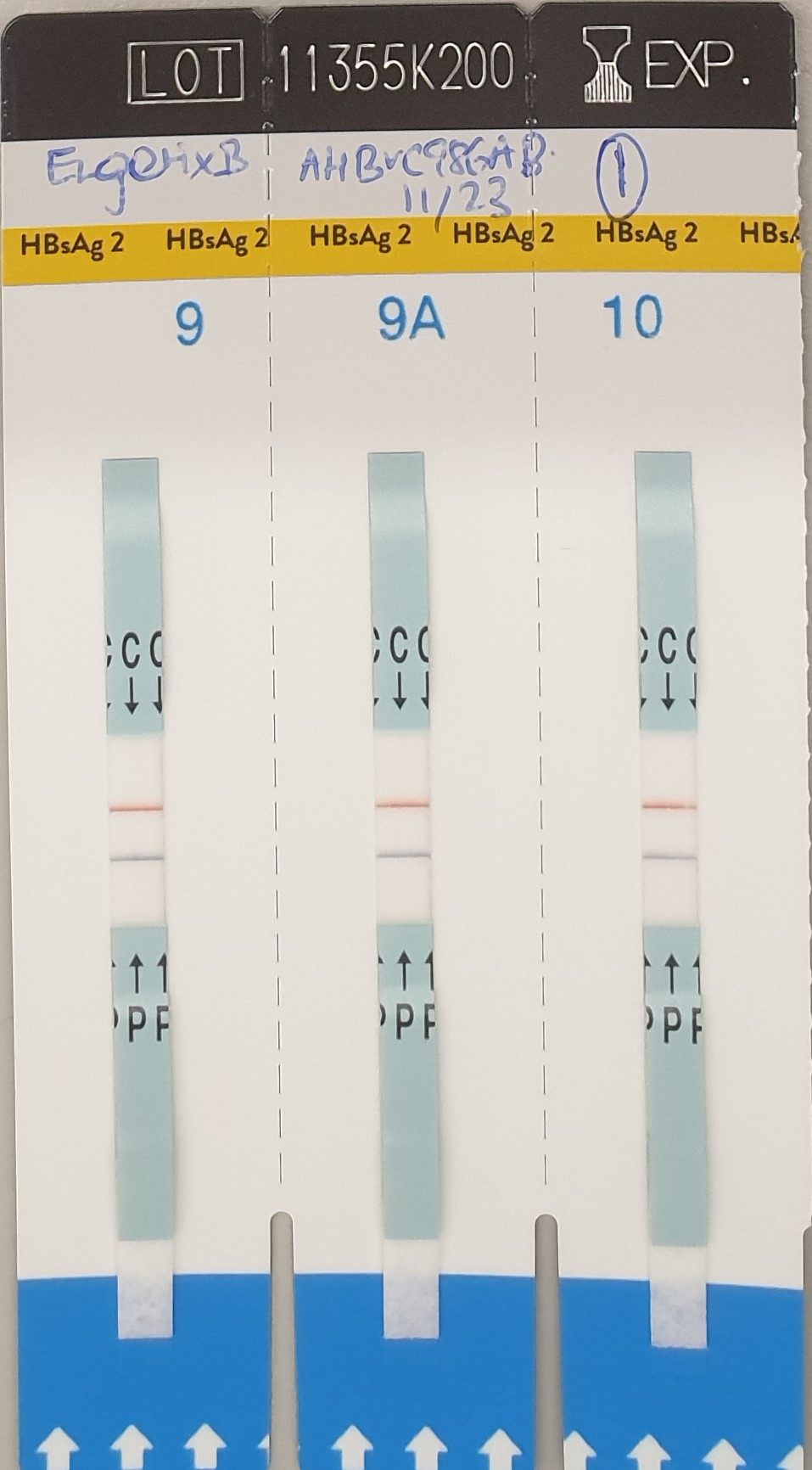

Supplement: Supplementary data [file EMS207833-supplement-Supplementary_data.zip › Initial assessment/Engerix B/Engerix B_Batch 2_Vial 1.jpg]

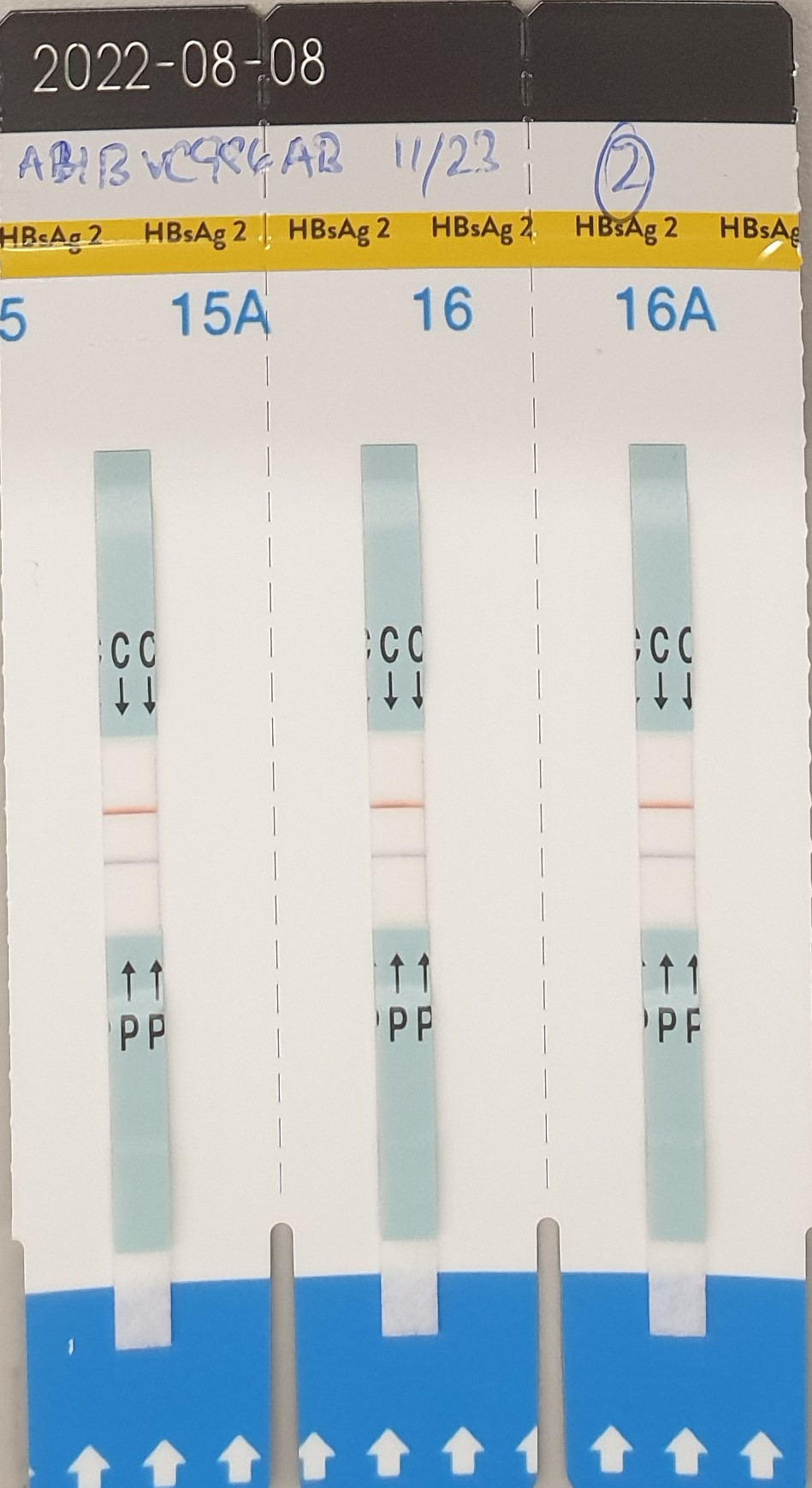

Supplement: Supplementary data [file EMS207833-supplement-Supplementary_data.zip › Initial assessment/Engerix B/Engerix B_Batch 2_Vial 2.jpg]

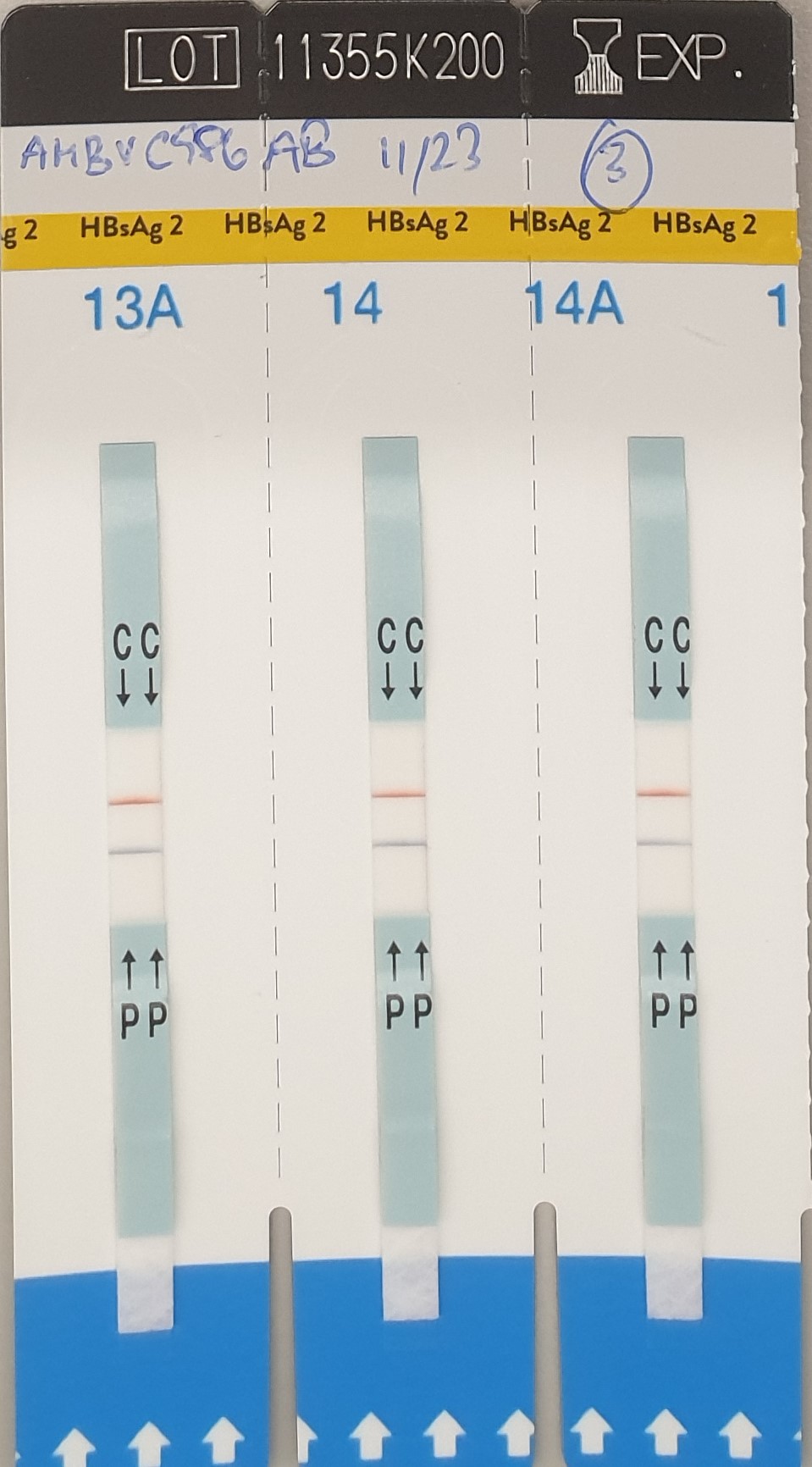

Supplement: Supplementary data [file EMS207833-supplement-Supplementary_data.zip › Initial assessment/Engerix B/Engerix B_Batch 2_Vial 3.jpg]

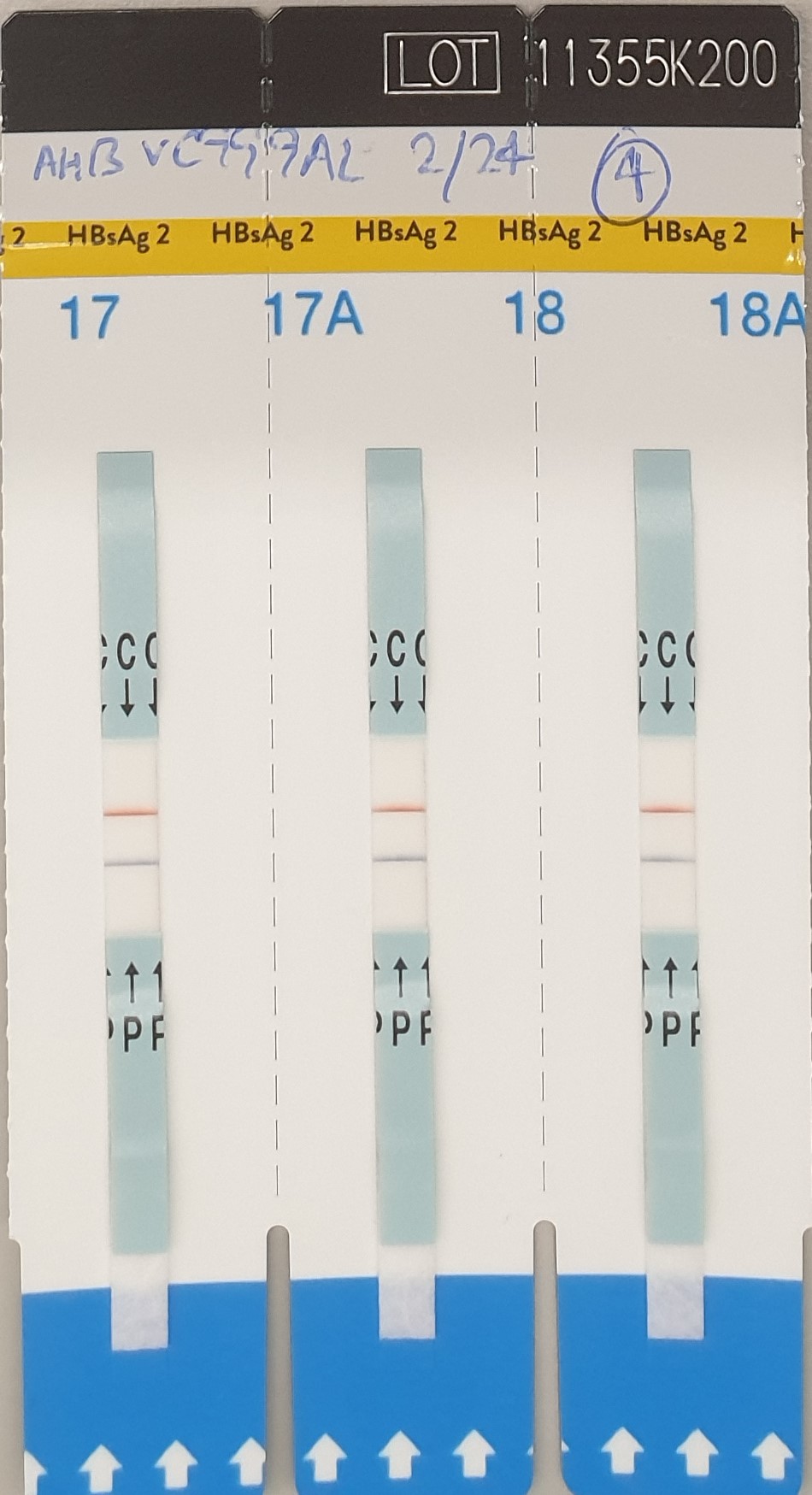

Supplement: Supplementary data [file EMS207833-supplement-Supplementary_data.zip › Initial assessment/Engerix B/Engerix B_Batch 3_Vial 1.jpg]

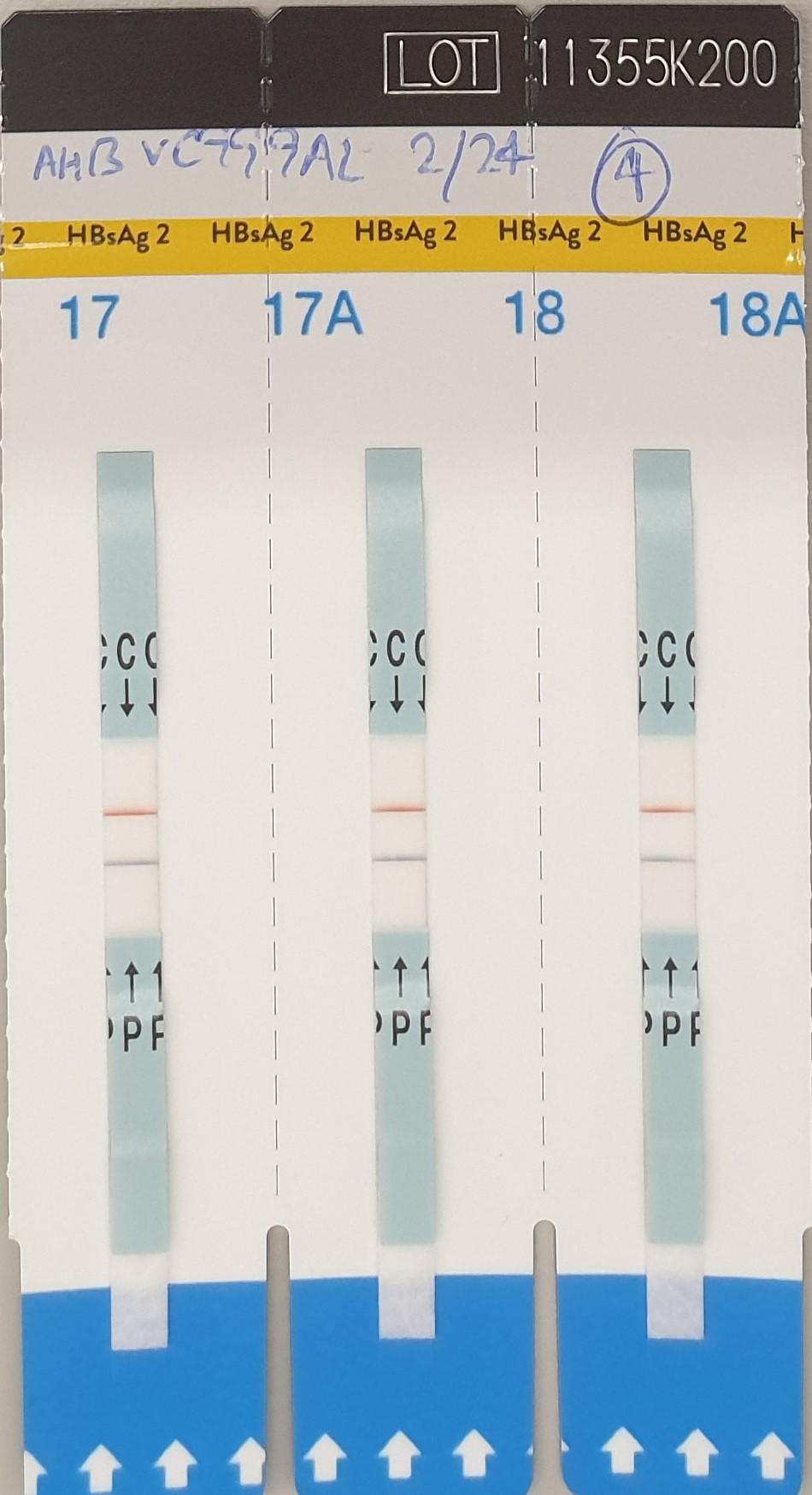

Supplement: Supplementary data [file EMS207833-supplement-Supplementary_data.zip › Initial assessment/Engerix B/Engerix B_Batch 3_Vial 2.jpg]

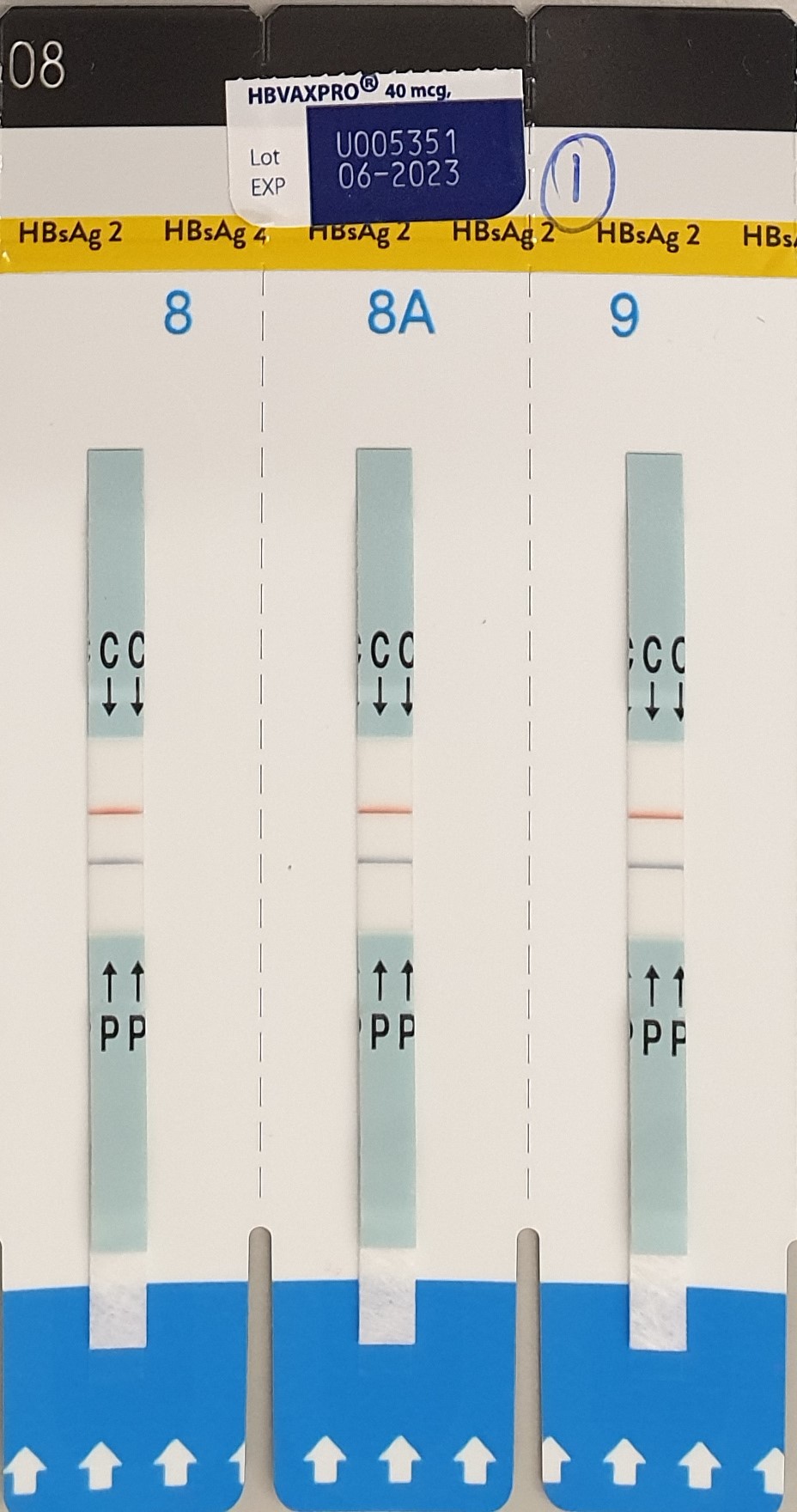

Supplement: Supplementary data [file EMS207833-supplement-Supplementary_data.zip › Initial assessment/HBVAXPRO/HBVAXPRO_Batch 1_Vial 1.jpg]

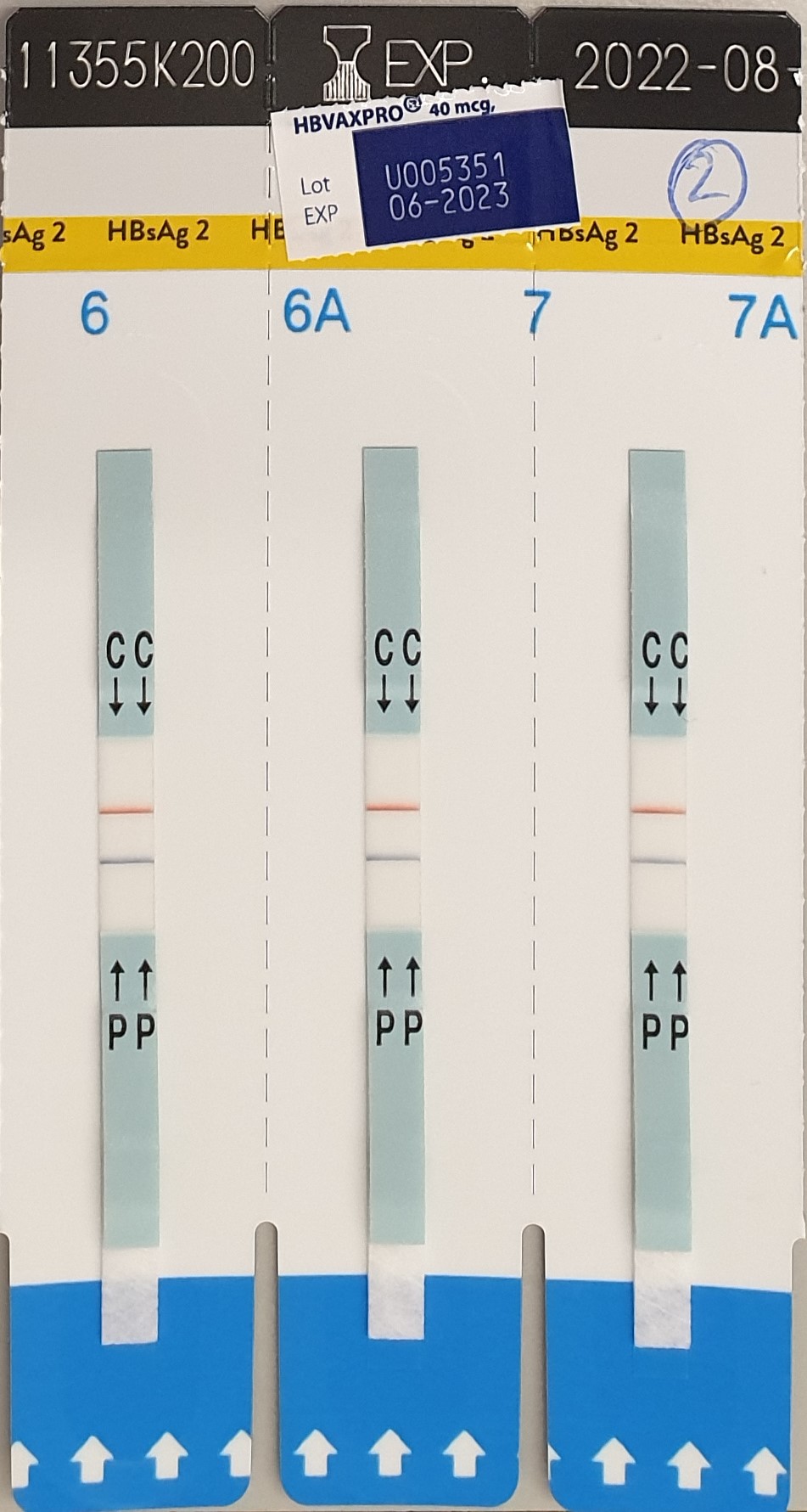

Supplement: Supplementary data [file EMS207833-supplement-Supplementary_data.zip › Initial assessment/HBVAXPRO/HBVAXPRO_Batch 1_Vial 2.jpg]

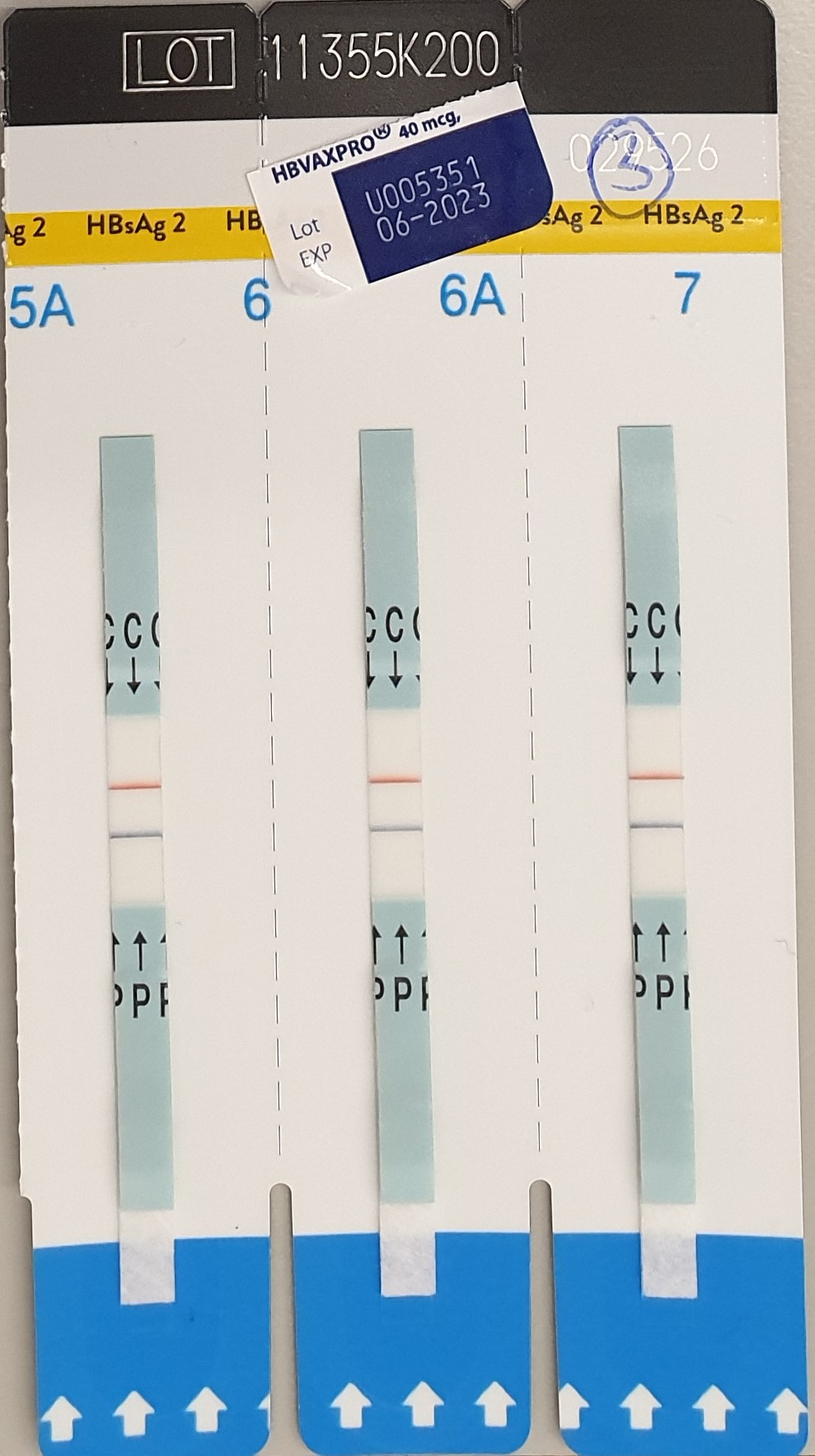

Supplement: Supplementary data [file EMS207833-supplement-Supplementary_data.zip › Initial assessment/HBVAXPRO/HBVAXPRO_Batch 1_Vial 3.jpg]

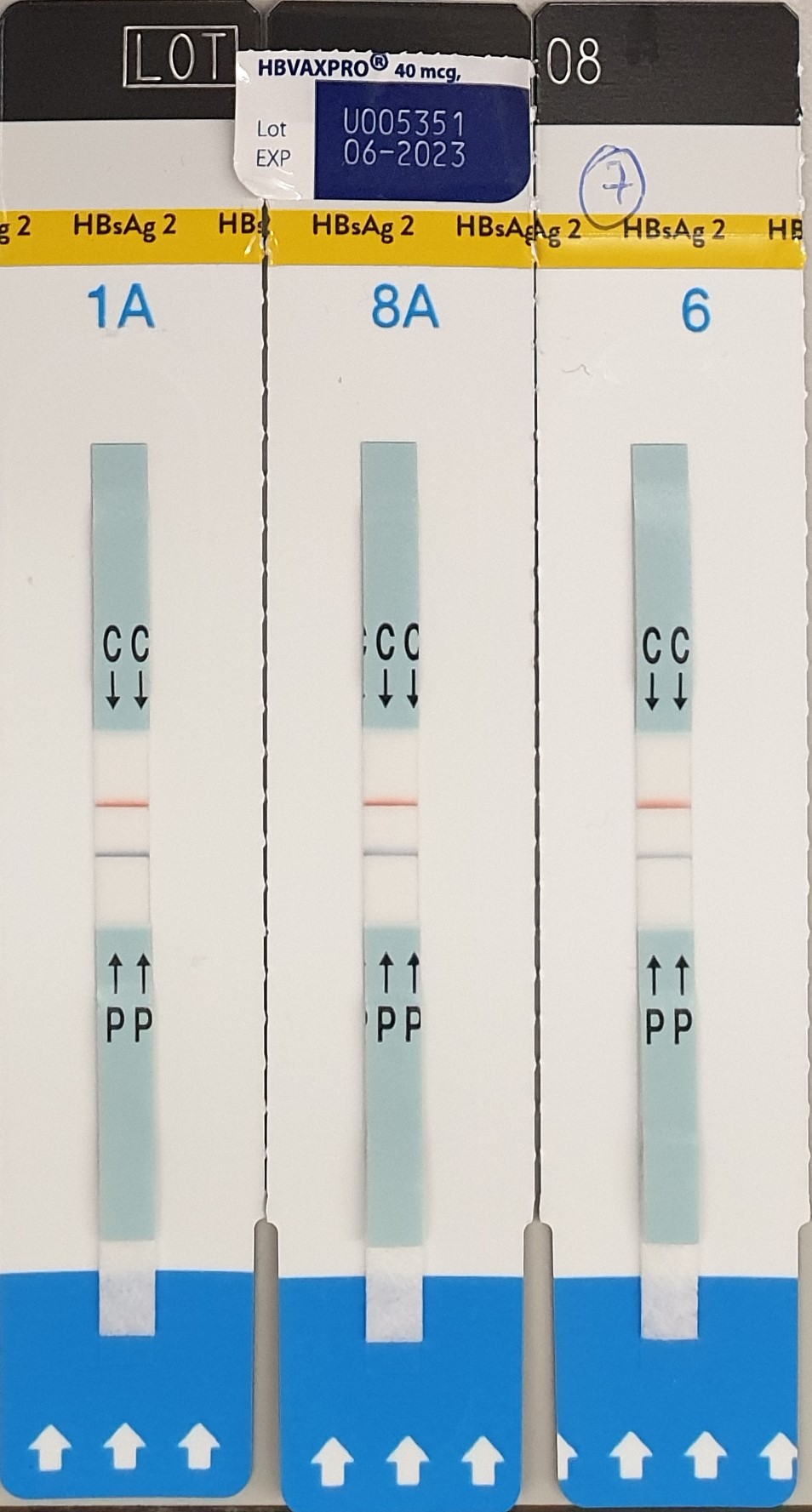

Supplement: Supplementary data [file EMS207833-supplement-Supplementary_data.zip › Initial assessment/HBVAXPRO/HBVAXPRO_Batch 1_Vial 4.jpg]

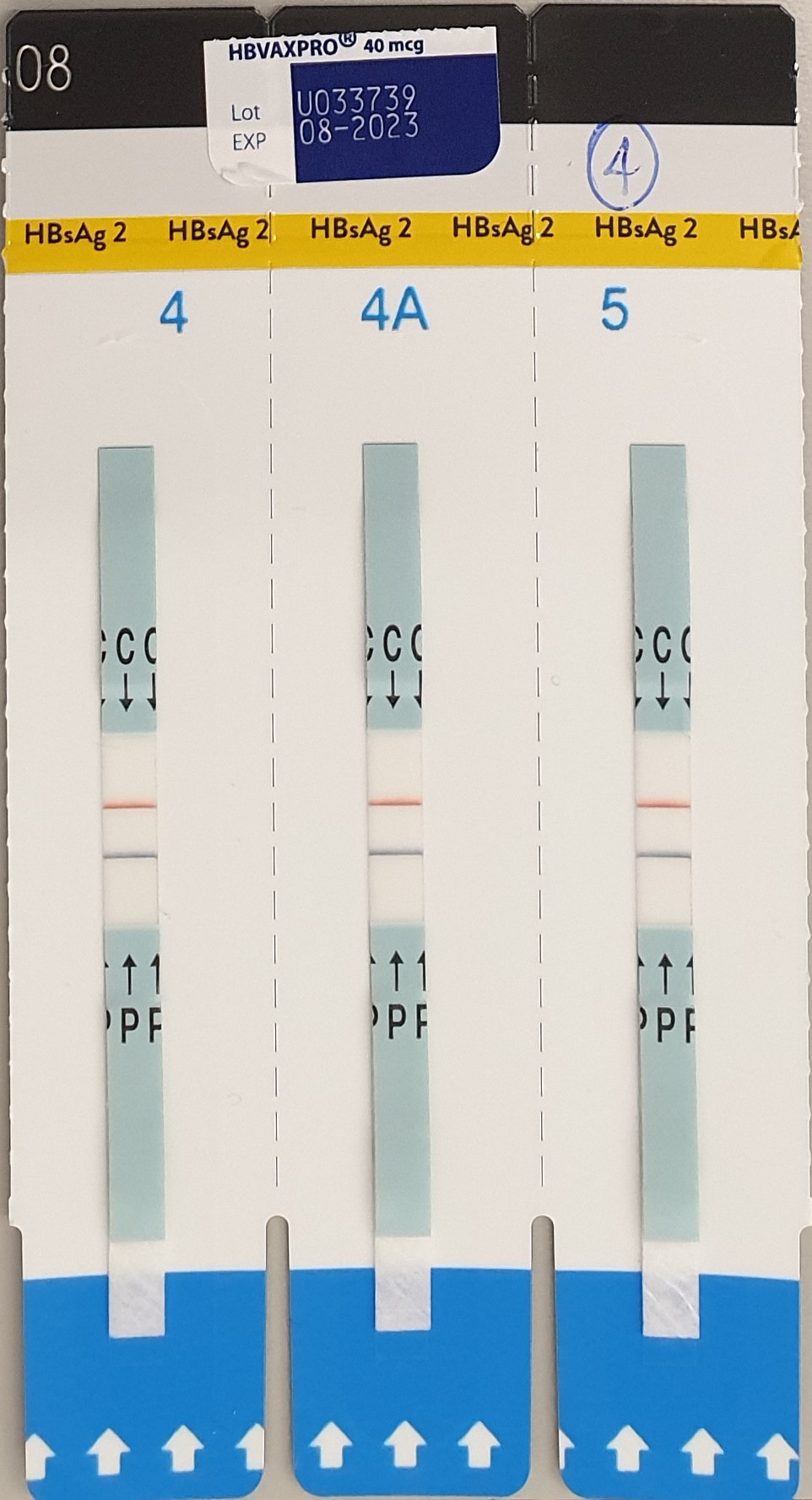

Supplement: Supplementary data [file EMS207833-supplement-Supplementary_data.zip › Initial assessment/HBVAXPRO/HBVAXPRO_Batch 2_Vial 1.jpg]

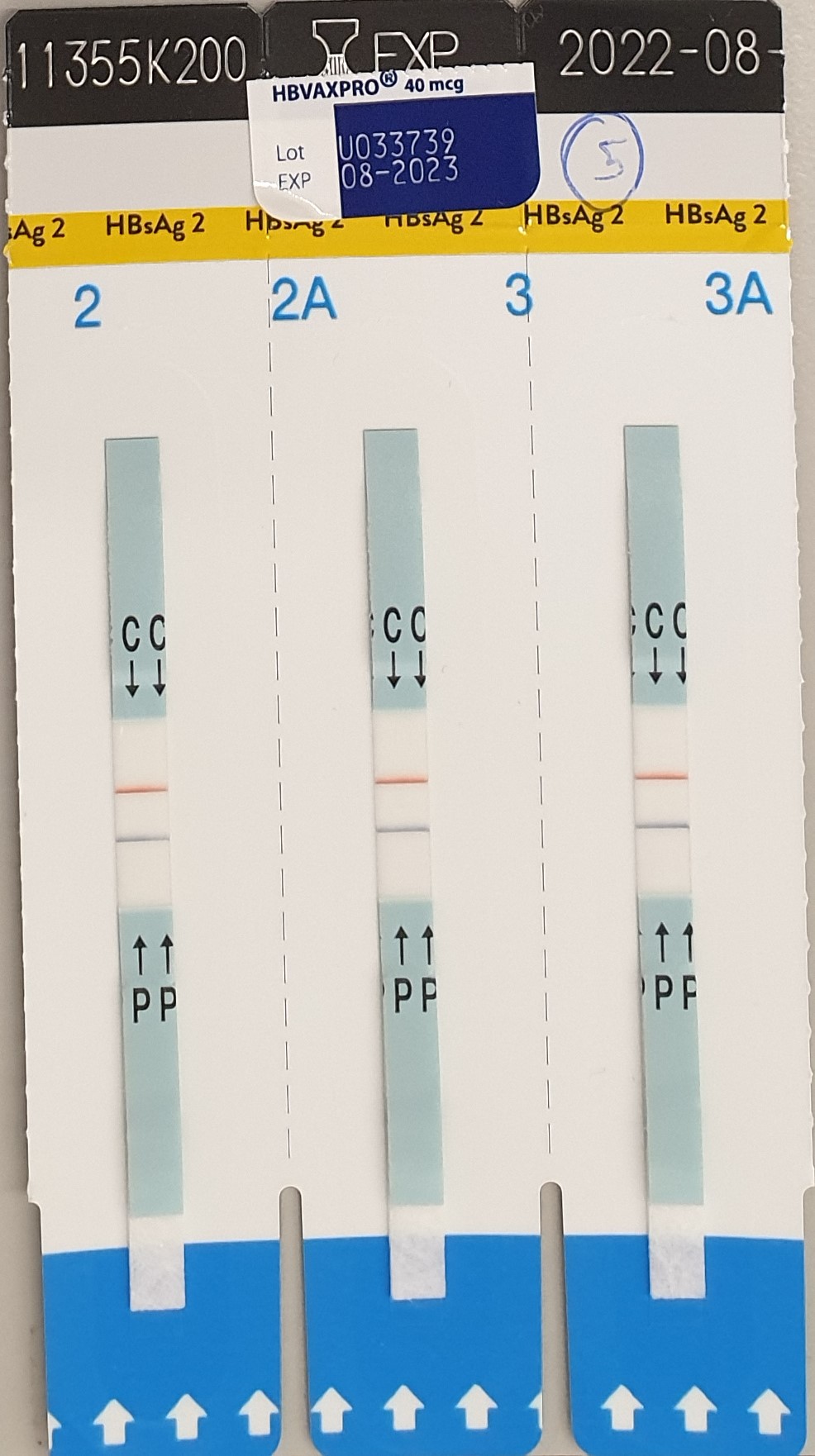

Supplement: Supplementary data [file EMS207833-supplement-Supplementary_data.zip › Initial assessment/HBVAXPRO/HBVAXPRO_Batch 2_Vial 2.jpg]

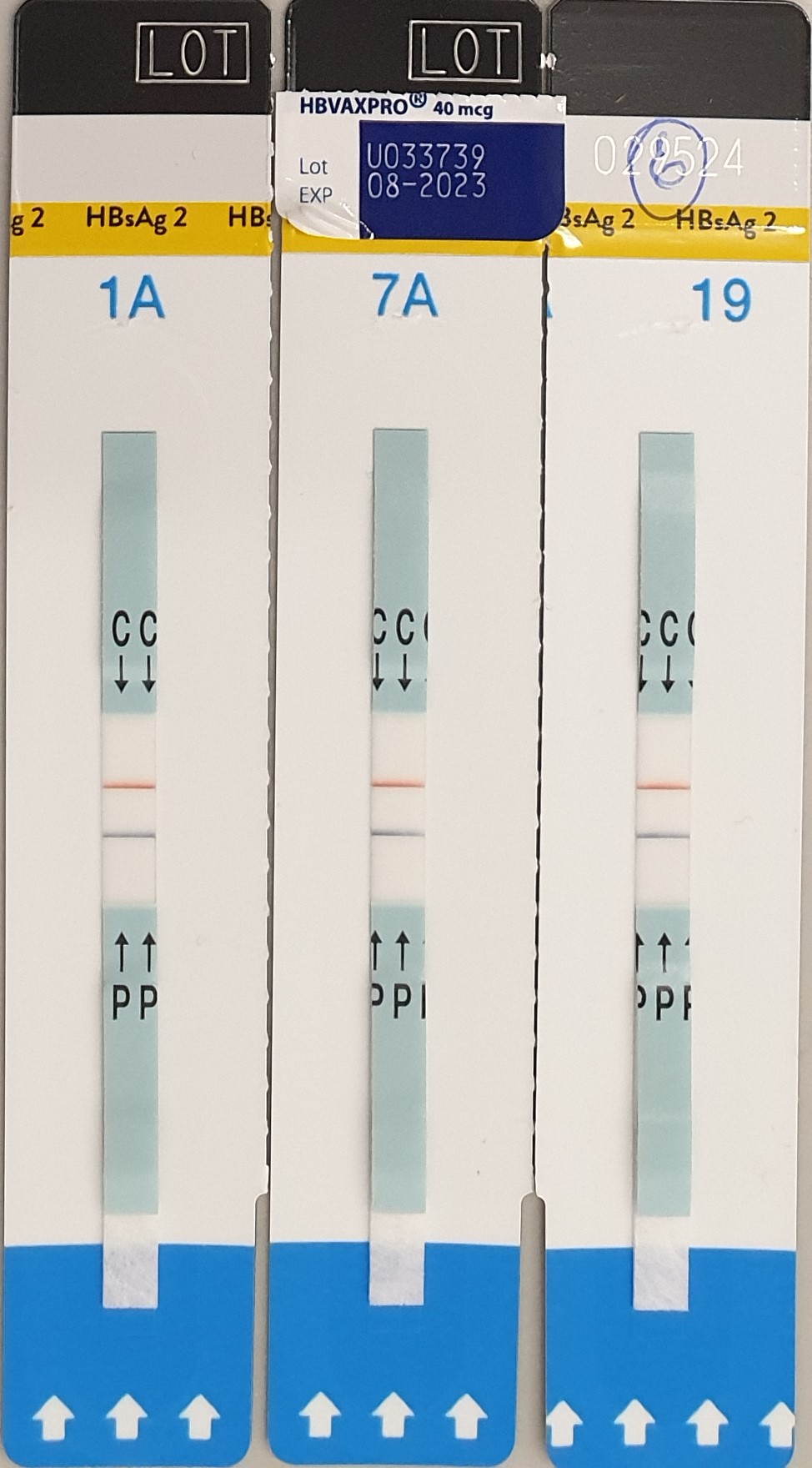

Supplement: Supplementary data [file EMS207833-supplement-Supplementary_data.zip › Initial assessment/HBVAXPRO/HBVAXPRO_Batch 2_Vial 3.jpg]

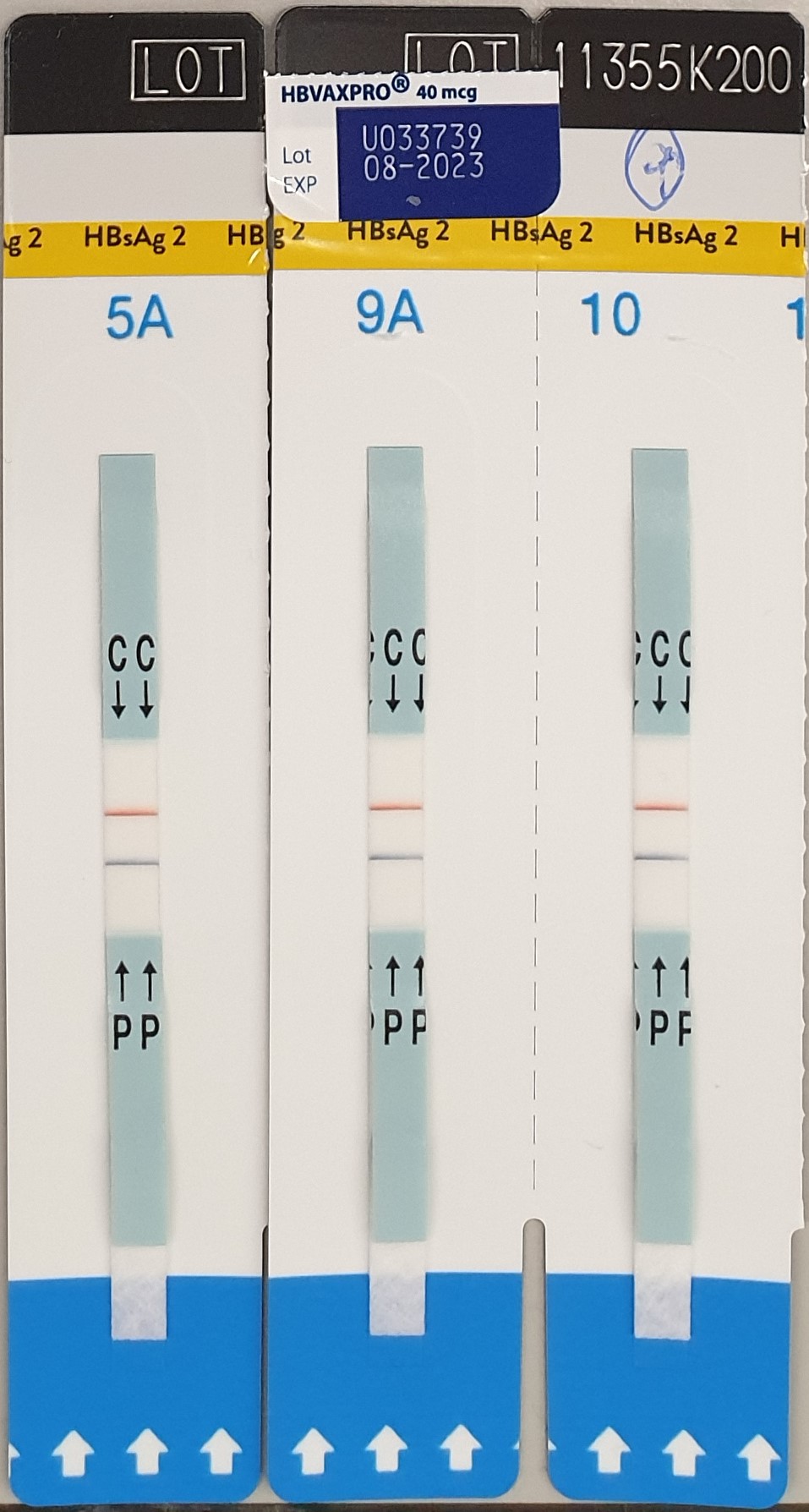

Supplement: Supplementary data [file EMS207833-supplement-Supplementary_data.zip › Initial assessment/HBVAXPRO/HBVAXPRO_Batch 2_Vial 4.jpg]

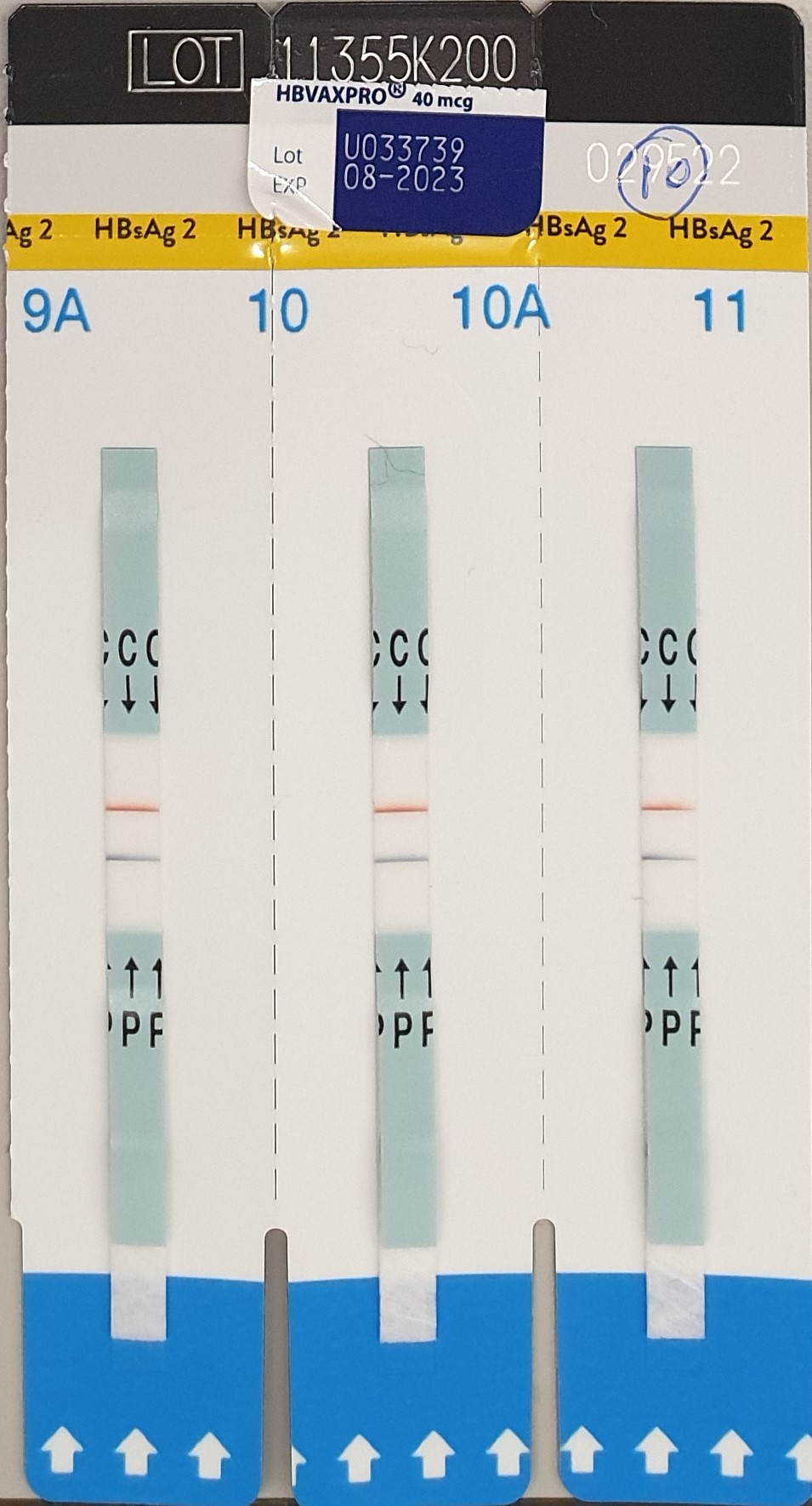

Supplement: Supplementary data [file EMS207833-supplement-Supplementary_data.zip › Initial assessment/HBVAXPRO/HBVAXPRO_Batch 2_Vial 5.jpg]

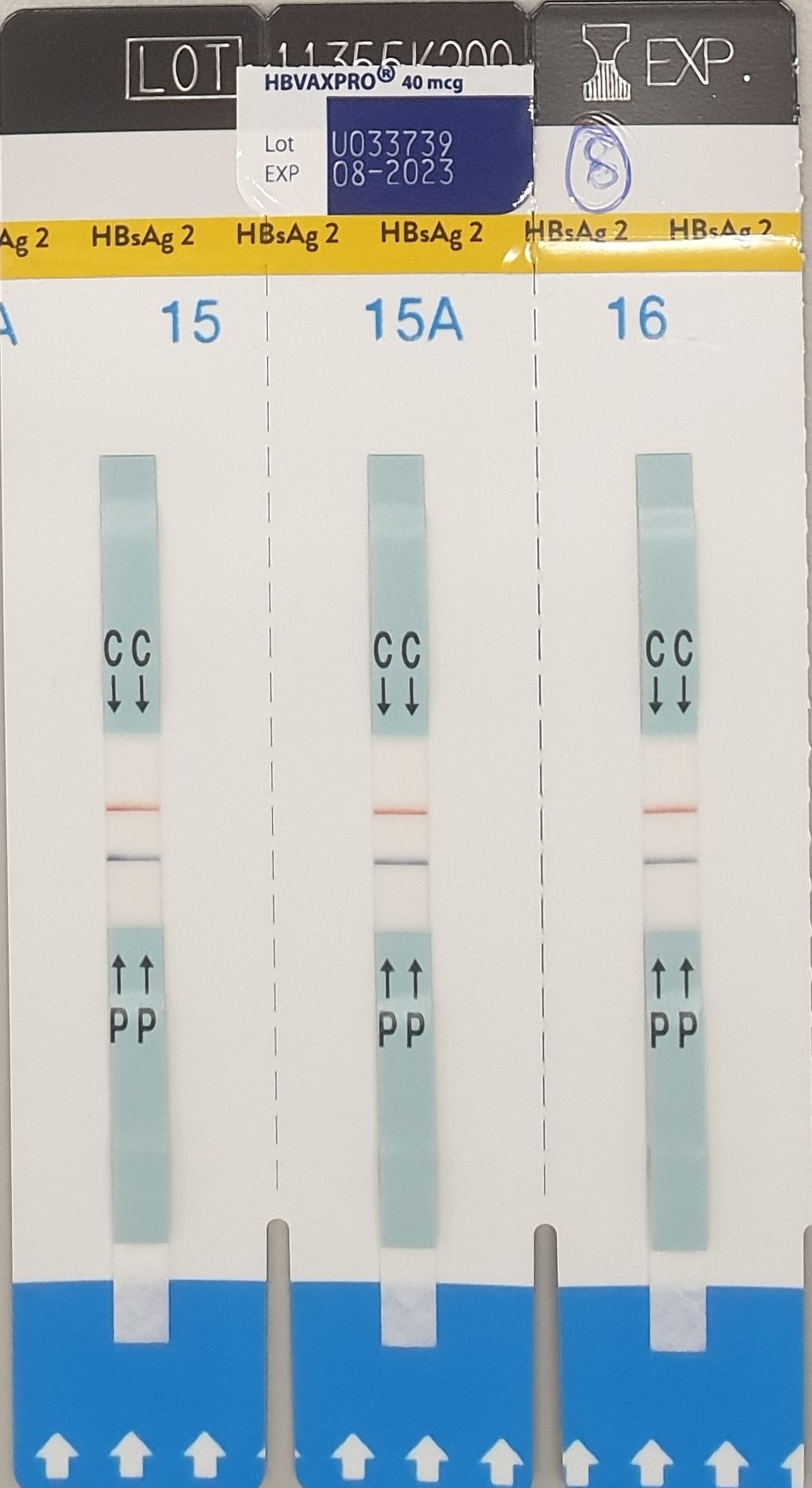

Supplement: Supplementary data [file EMS207833-supplement-Supplementary_data.zip › Initial assessment/HBVAXPRO/HBVAXPRO_Batch 2_Vial 6.jpg]

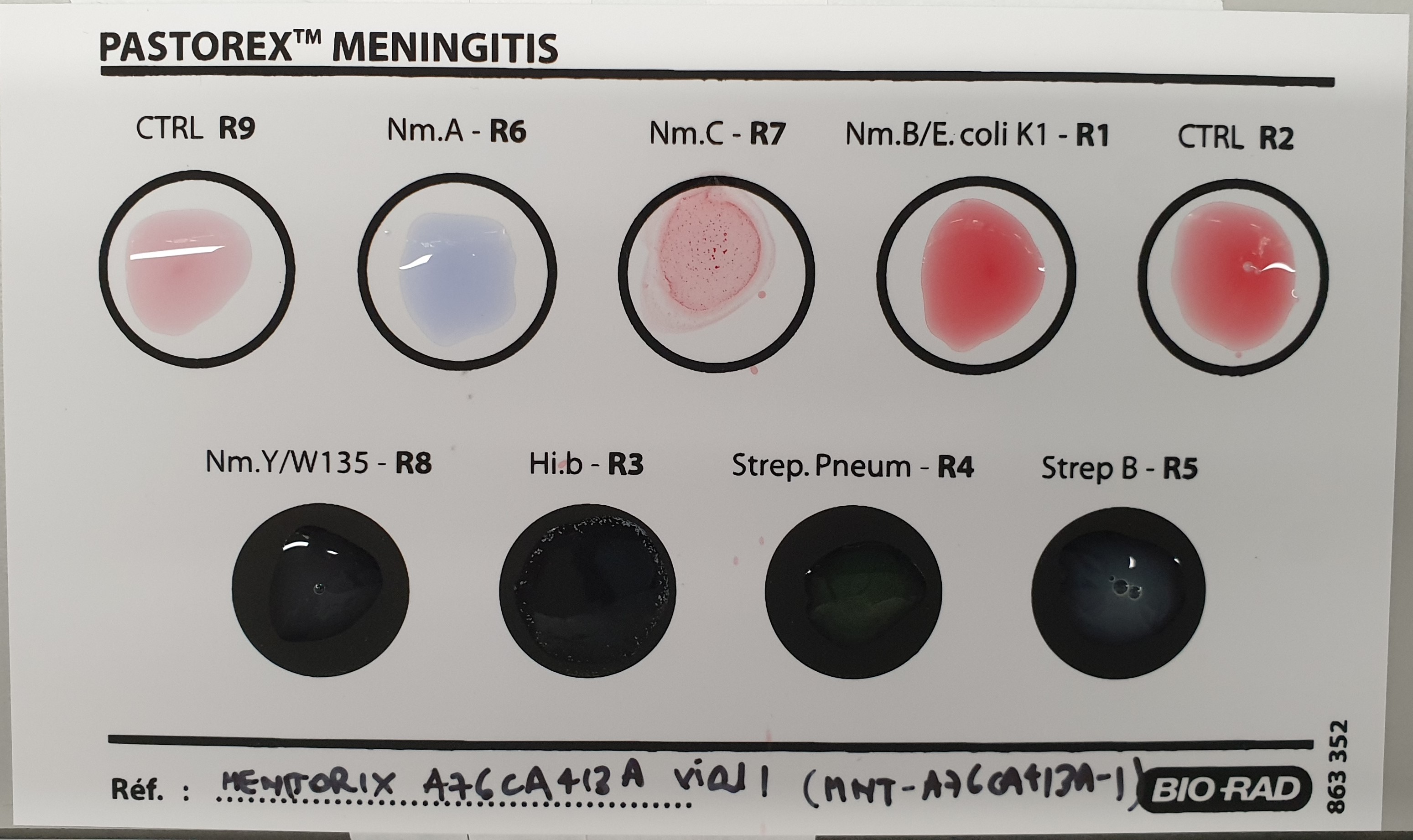

Supplement: Supplementary data [file EMS207833-supplement-Supplementary_data.zip › Initial assessment/Menitorix/Menitorix_Batch 1_Vial 1.jpg]

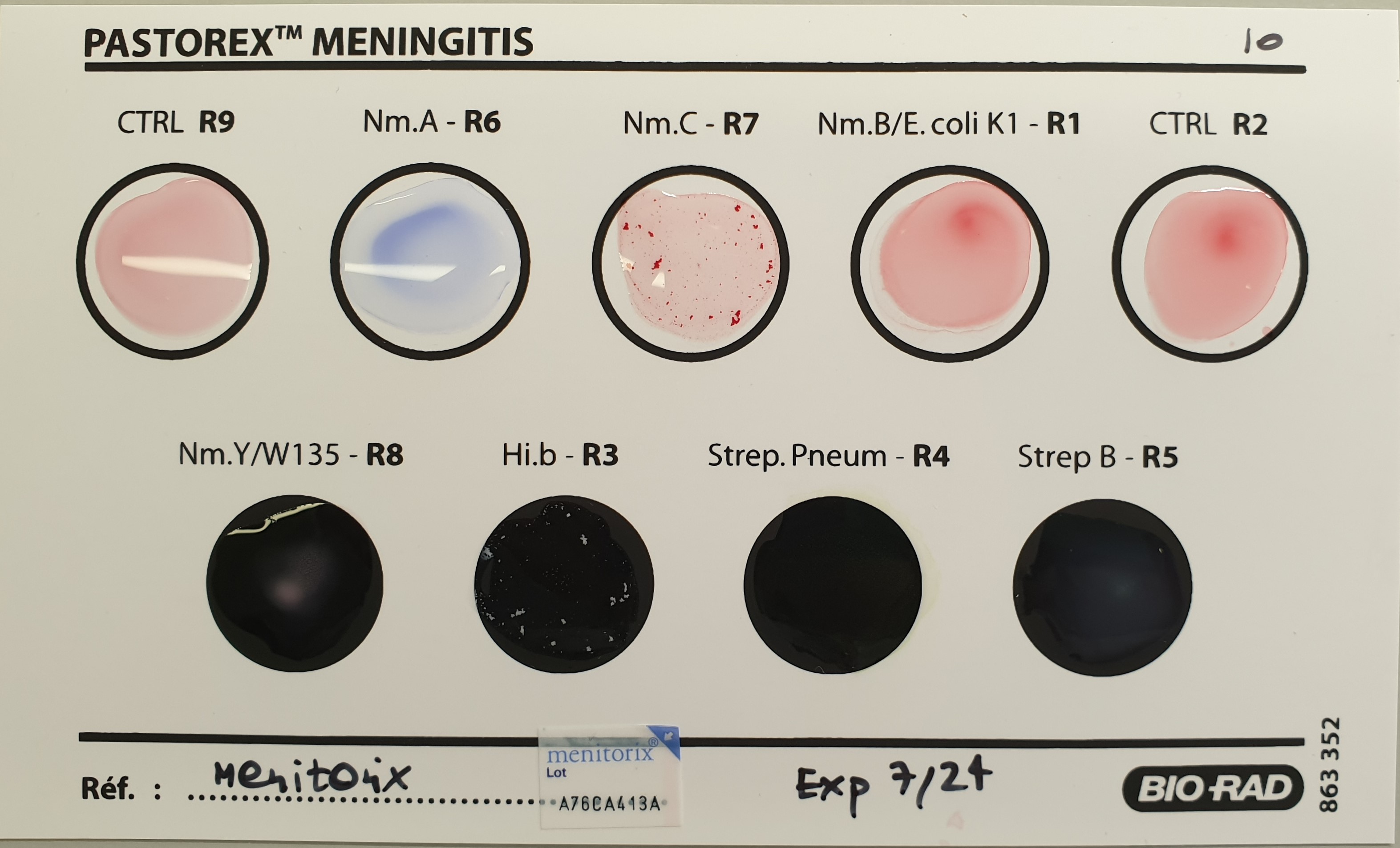

Supplement: Supplementary data [file EMS207833-supplement-Supplementary_data.zip › Initial assessment/Menitorix/Menitorix_Batch 1_Vial 10.jpg]

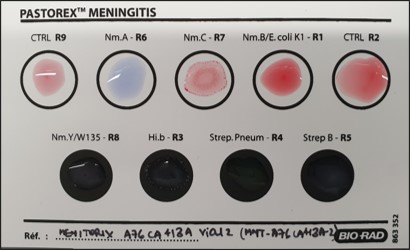

Supplement: Supplementary data [file EMS207833-supplement-Supplementary_data.zip › Initial assessment/Menitorix/Menitorix_Batch 1_Vial 2.jpg]

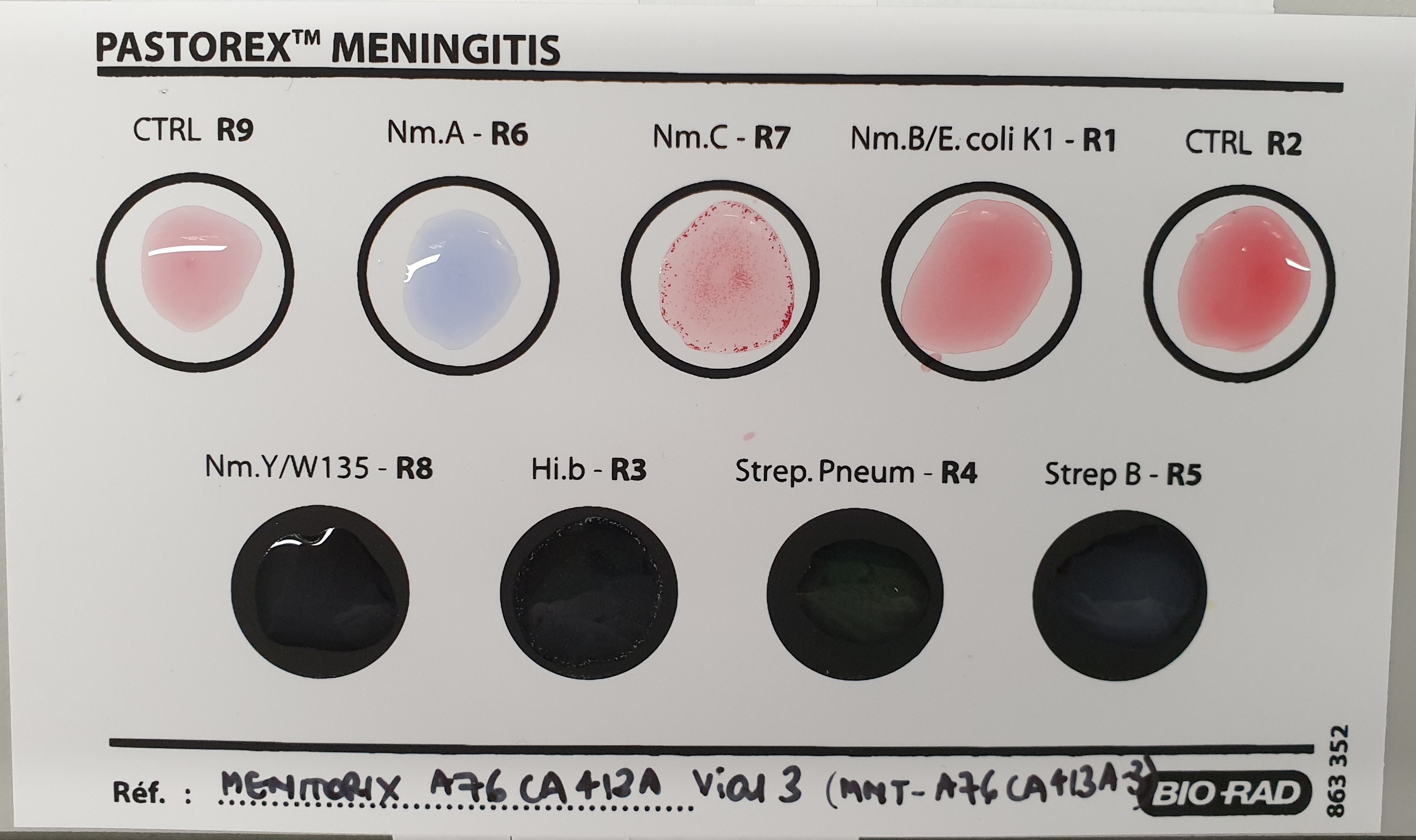

Supplement: Supplementary data [file EMS207833-supplement-Supplementary_data.zip › Initial assessment/Menitorix/Menitorix_Batch 1_Vial 3.jpg]

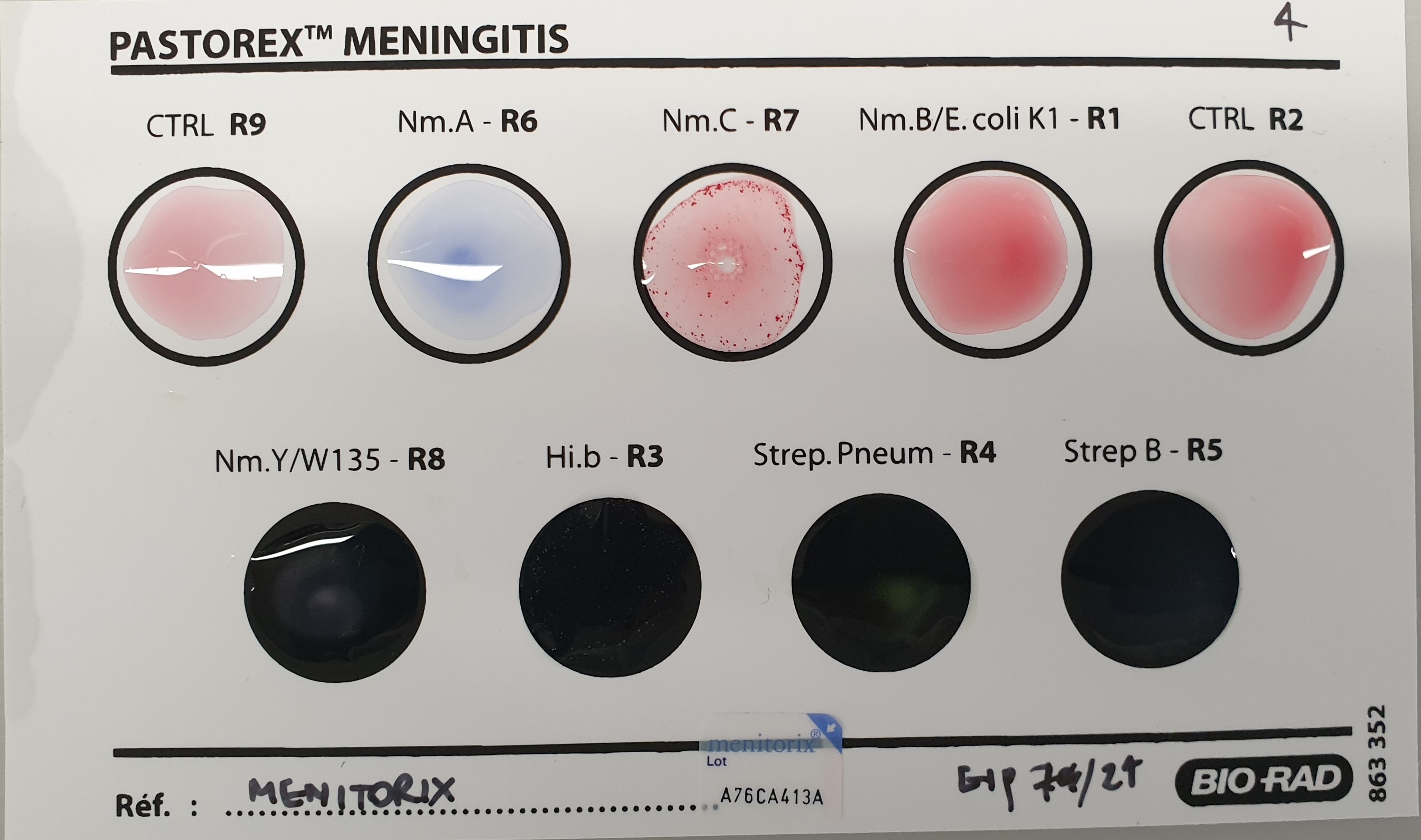

Supplement: Supplementary data [file EMS207833-supplement-Supplementary_data.zip › Initial assessment/Menitorix/Menitorix_Batch 1_Vial 4.jpg]

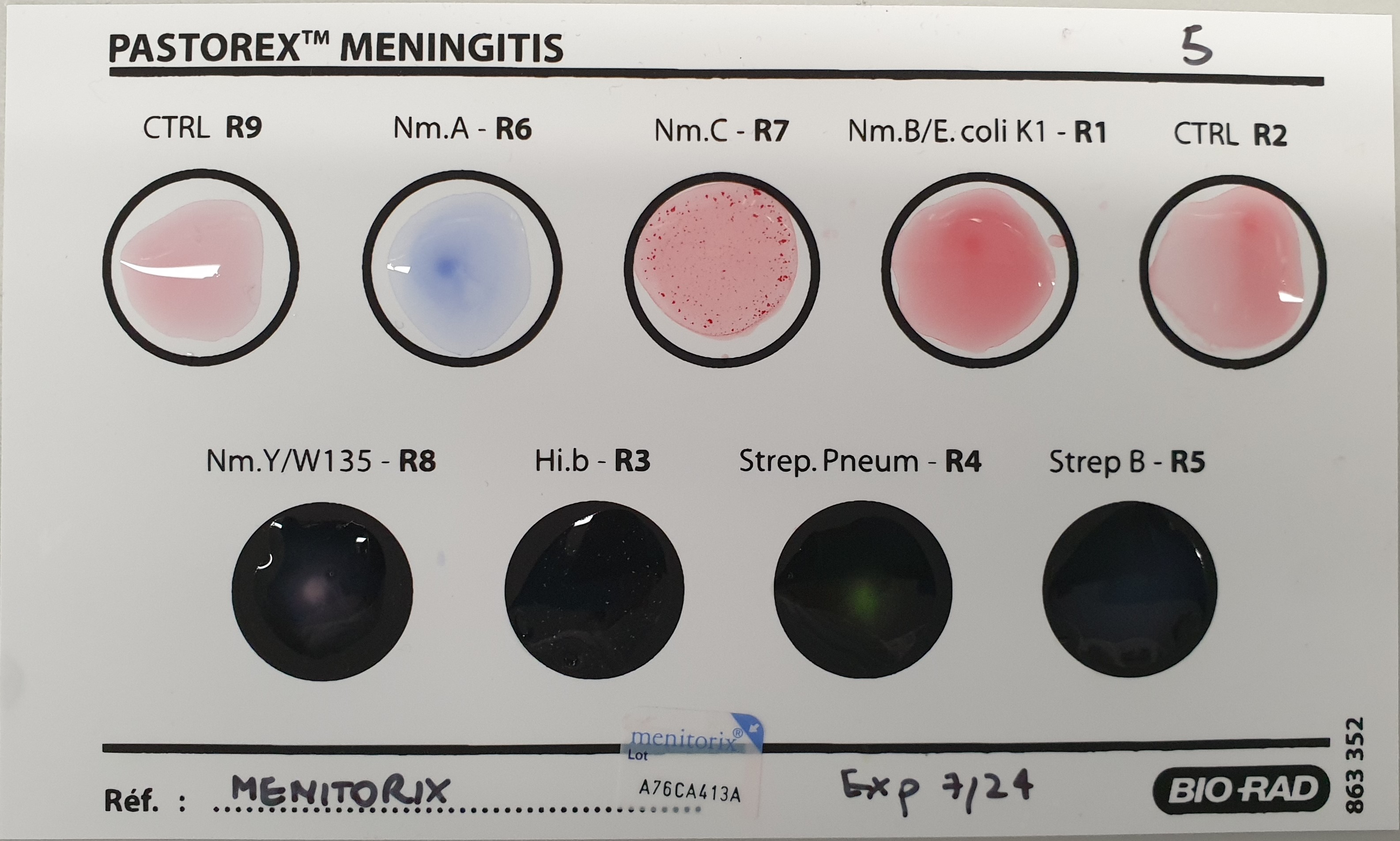

Supplement: Supplementary data [file EMS207833-supplement-Supplementary_data.zip › Initial assessment/Menitorix/Menitorix_Batch 1_Vial 5.jpg]

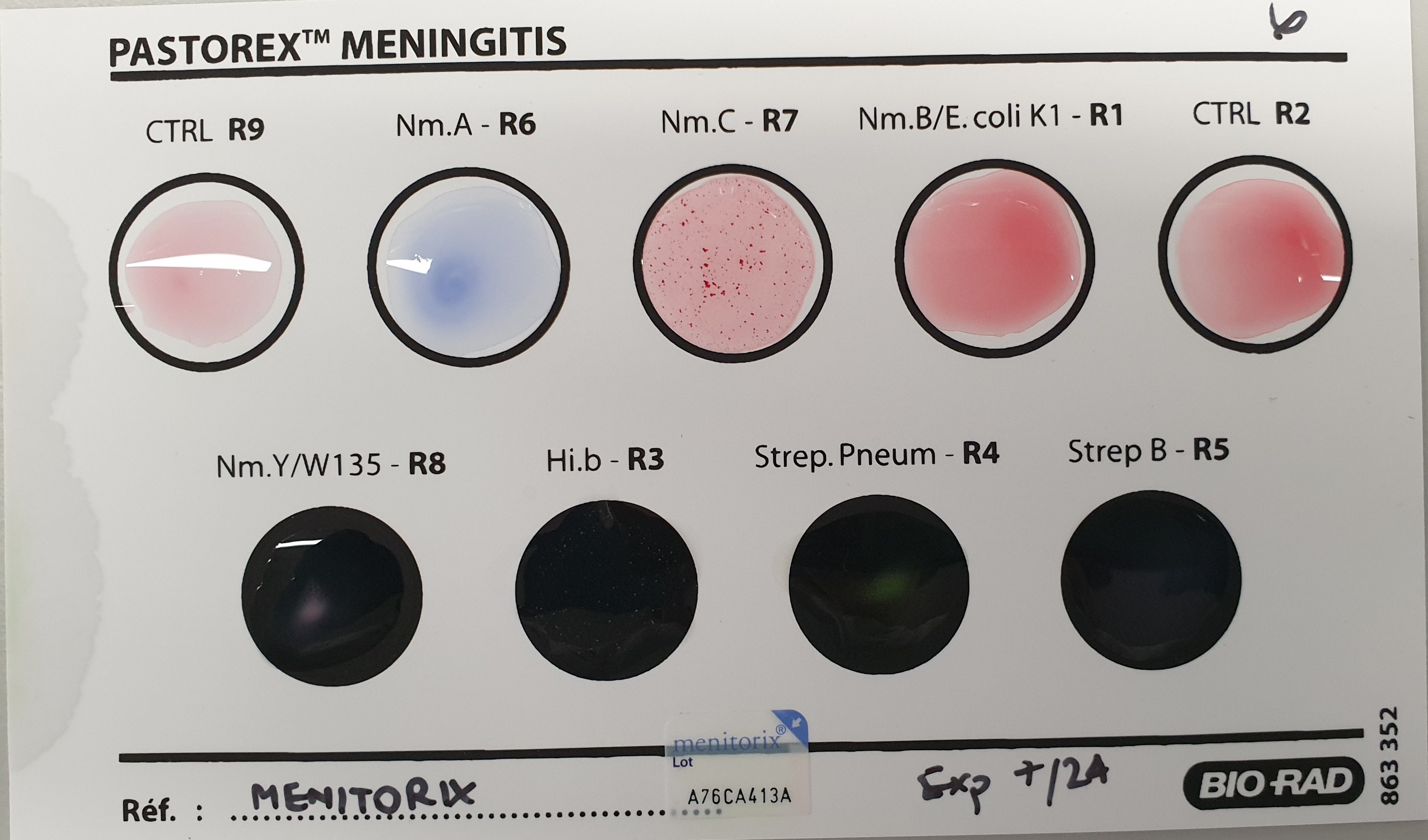

Supplement: Supplementary data [file EMS207833-supplement-Supplementary_data.zip › Initial assessment/Menitorix/Menitorix_Batch 1_Vial 6.jpg]

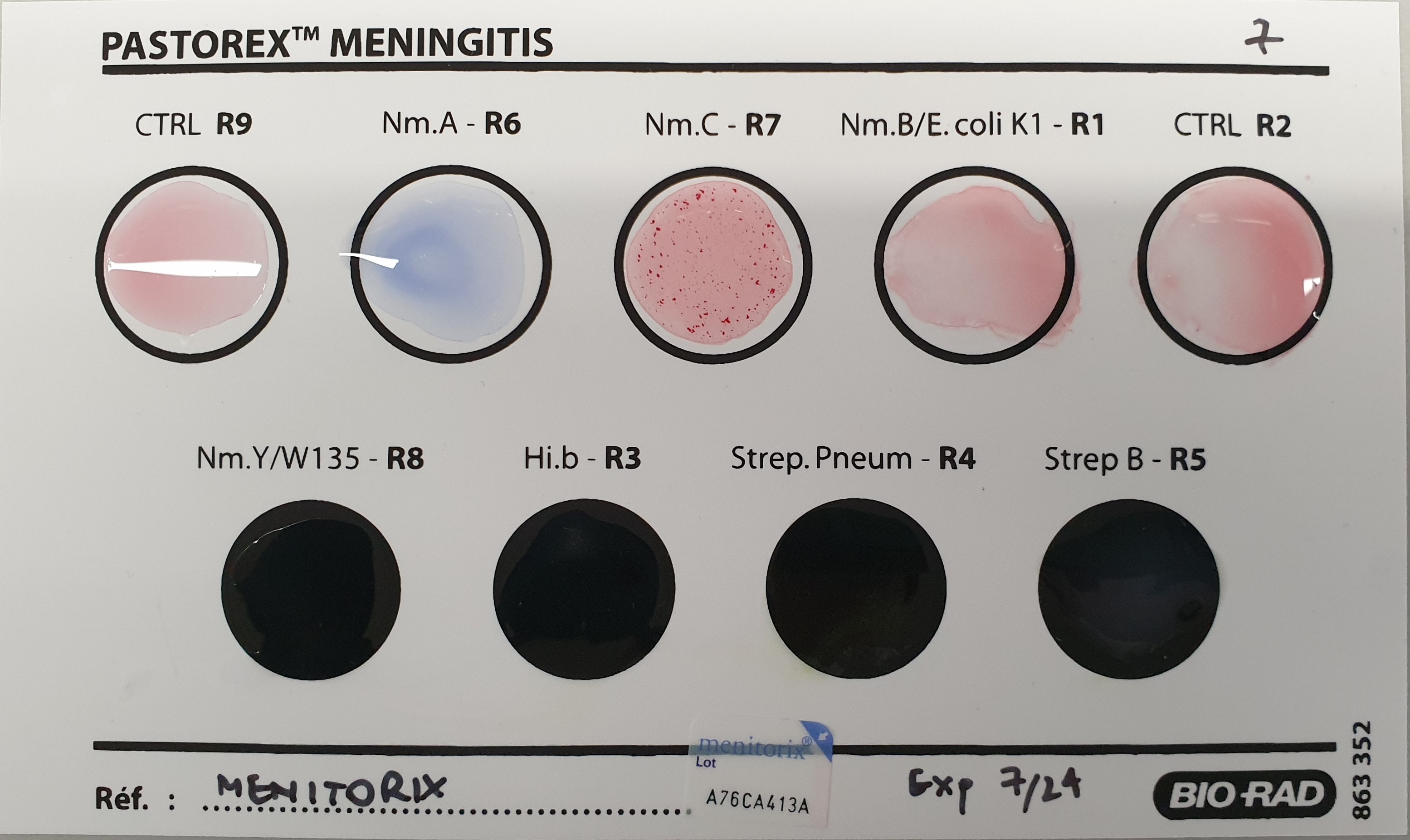

Supplement: Supplementary data [file EMS207833-supplement-Supplementary_data.zip › Initial assessment/Menitorix/Menitorix_Batch 1_Vial 7.jpg]

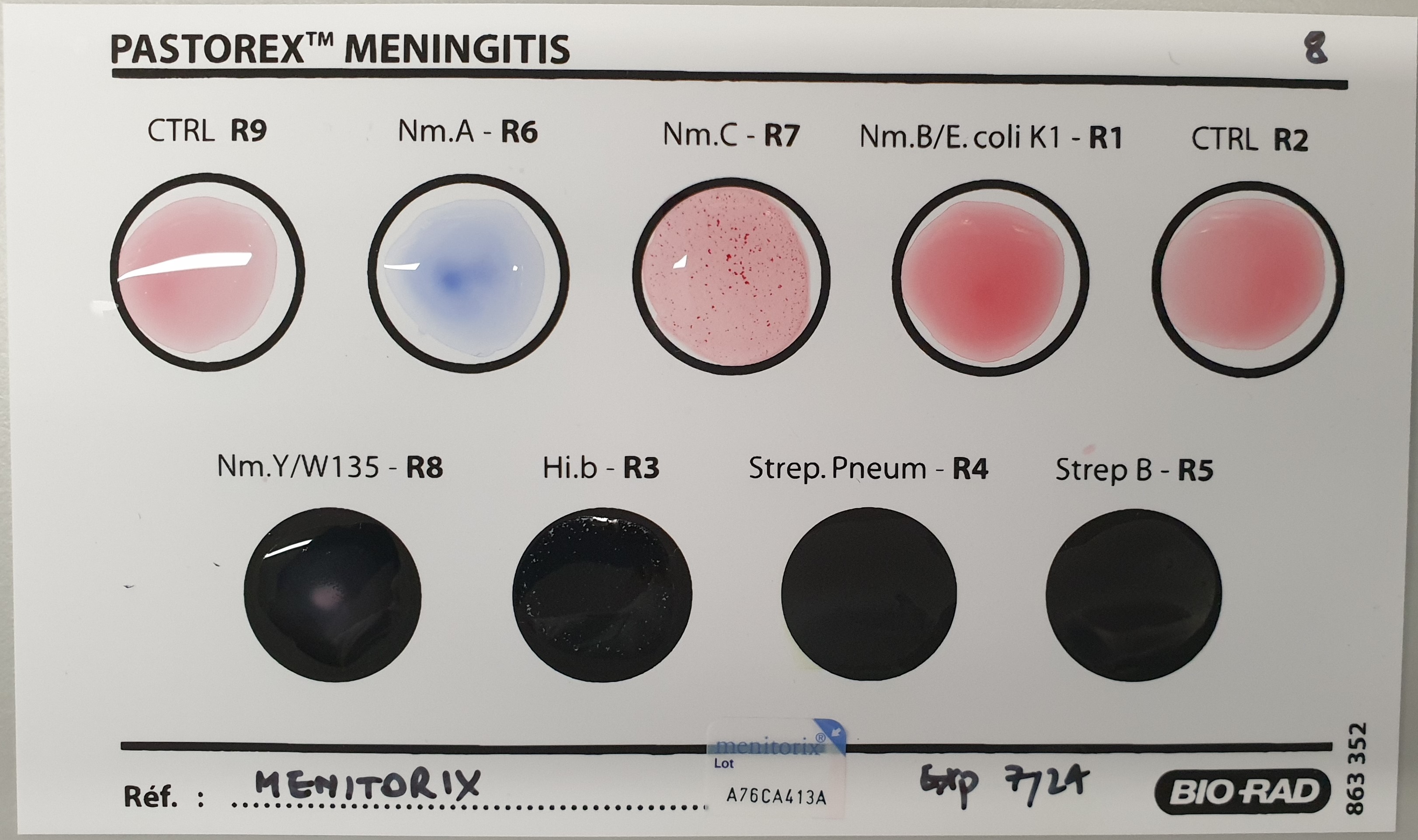

Supplement: Supplementary data [file EMS207833-supplement-Supplementary_data.zip › Initial assessment/Menitorix/Menitorix_Batch 1_Vial 8.jpg]

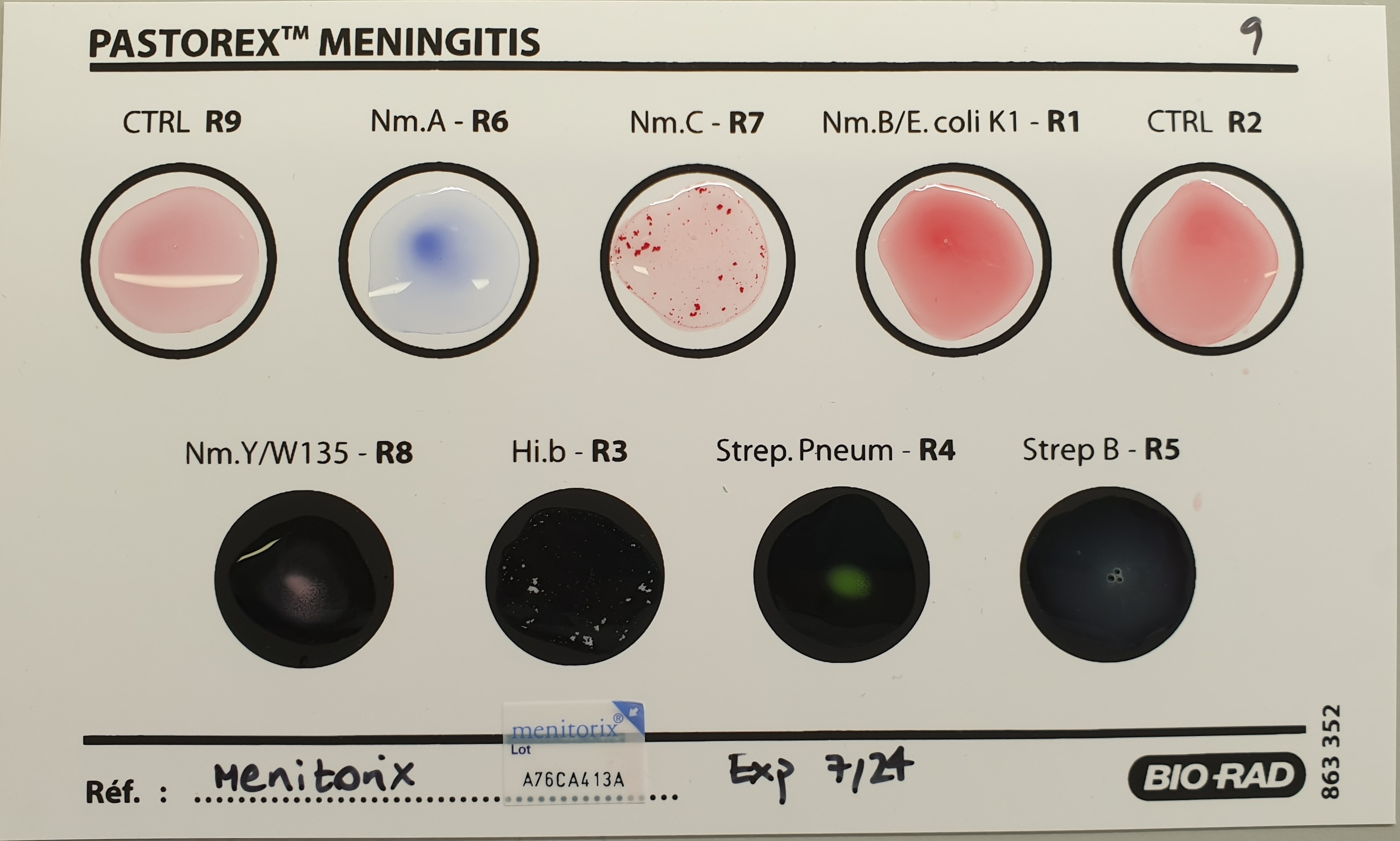

Supplement: Supplementary data [file EMS207833-supplement-Supplementary_data.zip › Initial assessment/Menitorix/Menitorix_Batch 1_Vial 9.jpg]

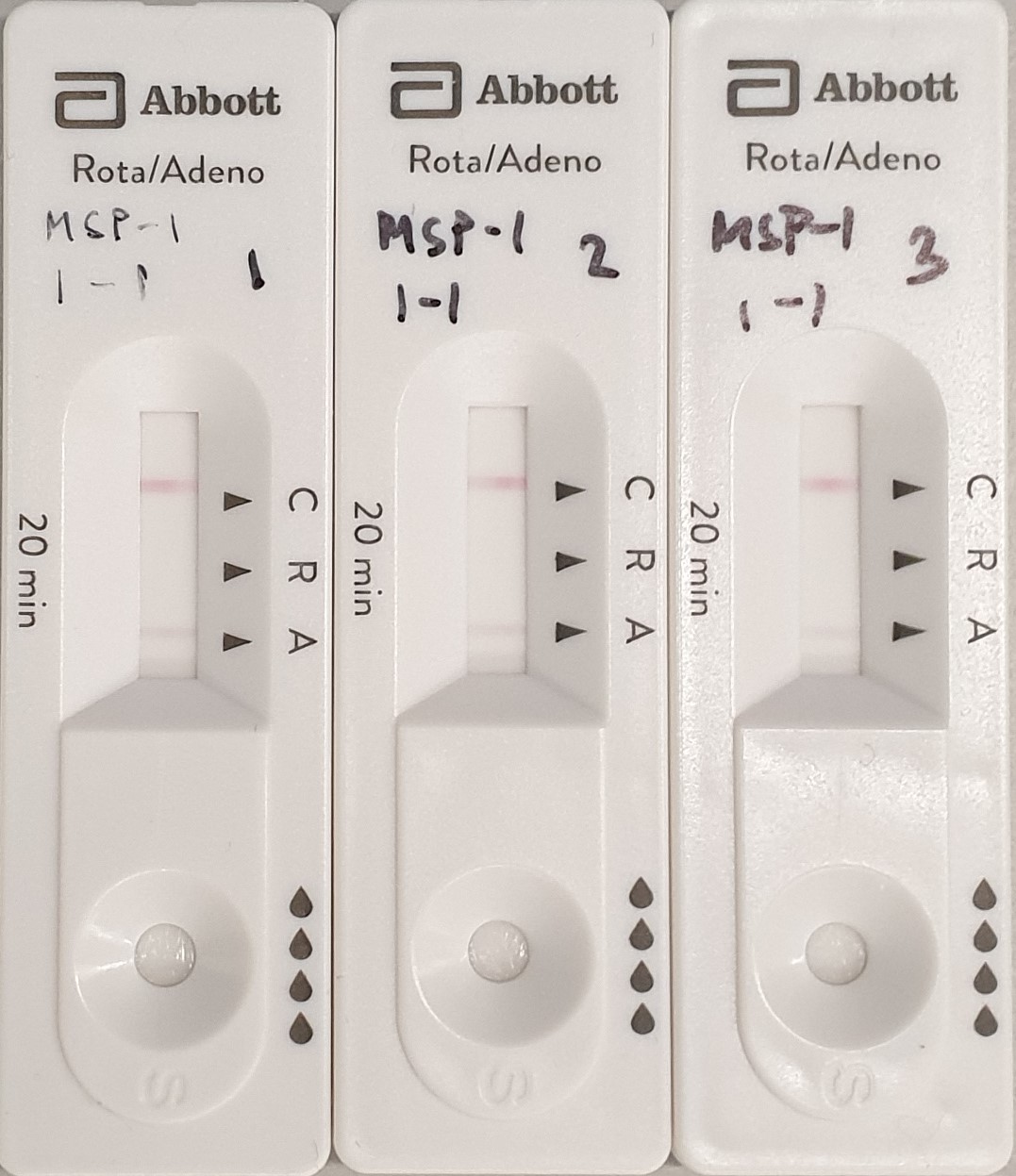

Supplement: Supplementary data [file EMS207833-supplement-Supplementary_data.zip › Initial assessment/MSP1/MSP1_Batch 1_Vial 1.jpg]

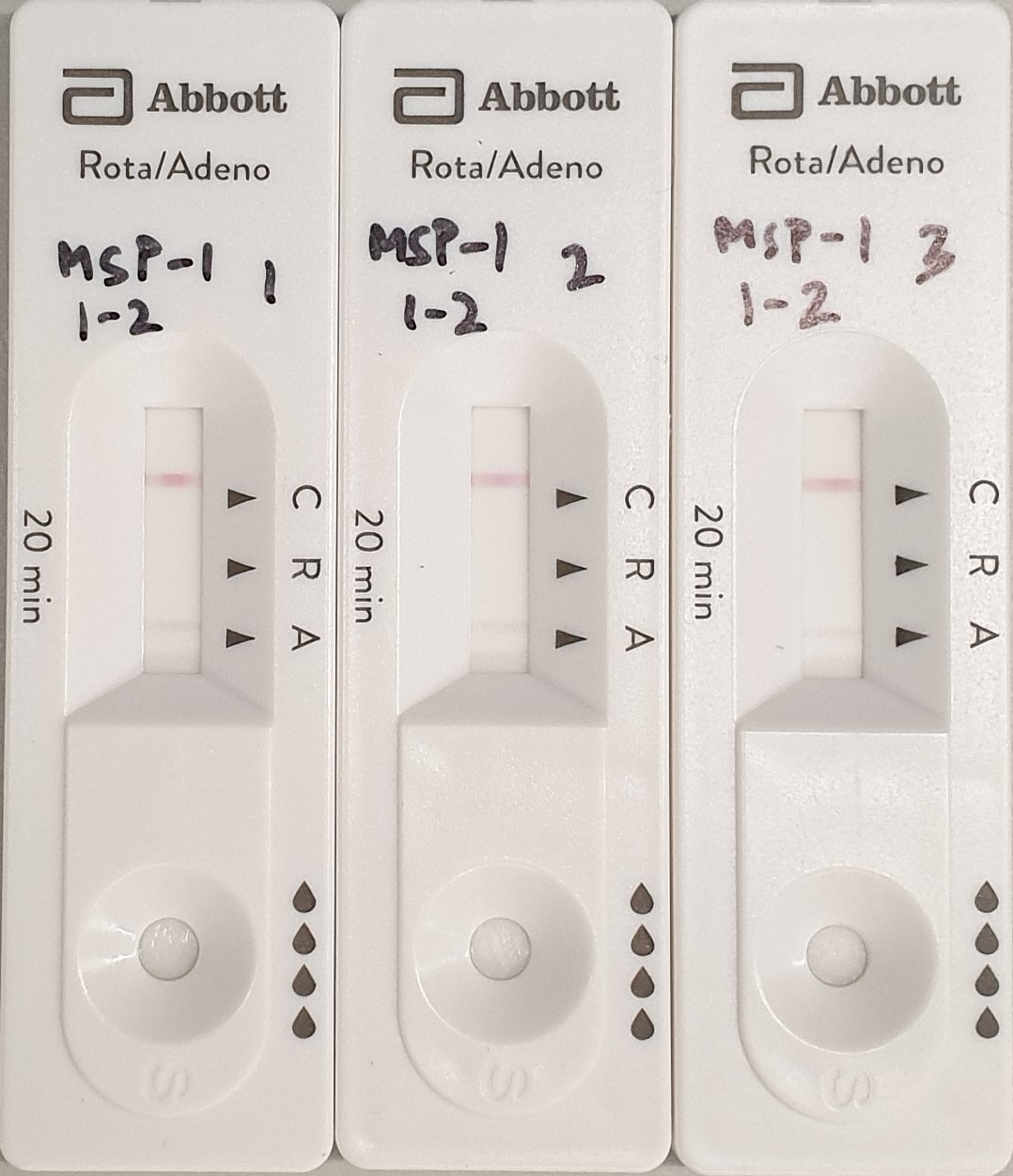

Supplement: Supplementary data [file EMS207833-supplement-Supplementary_data.zip › Initial assessment/MSP1/MSP1_Batch 1_Vial 2.jpg]

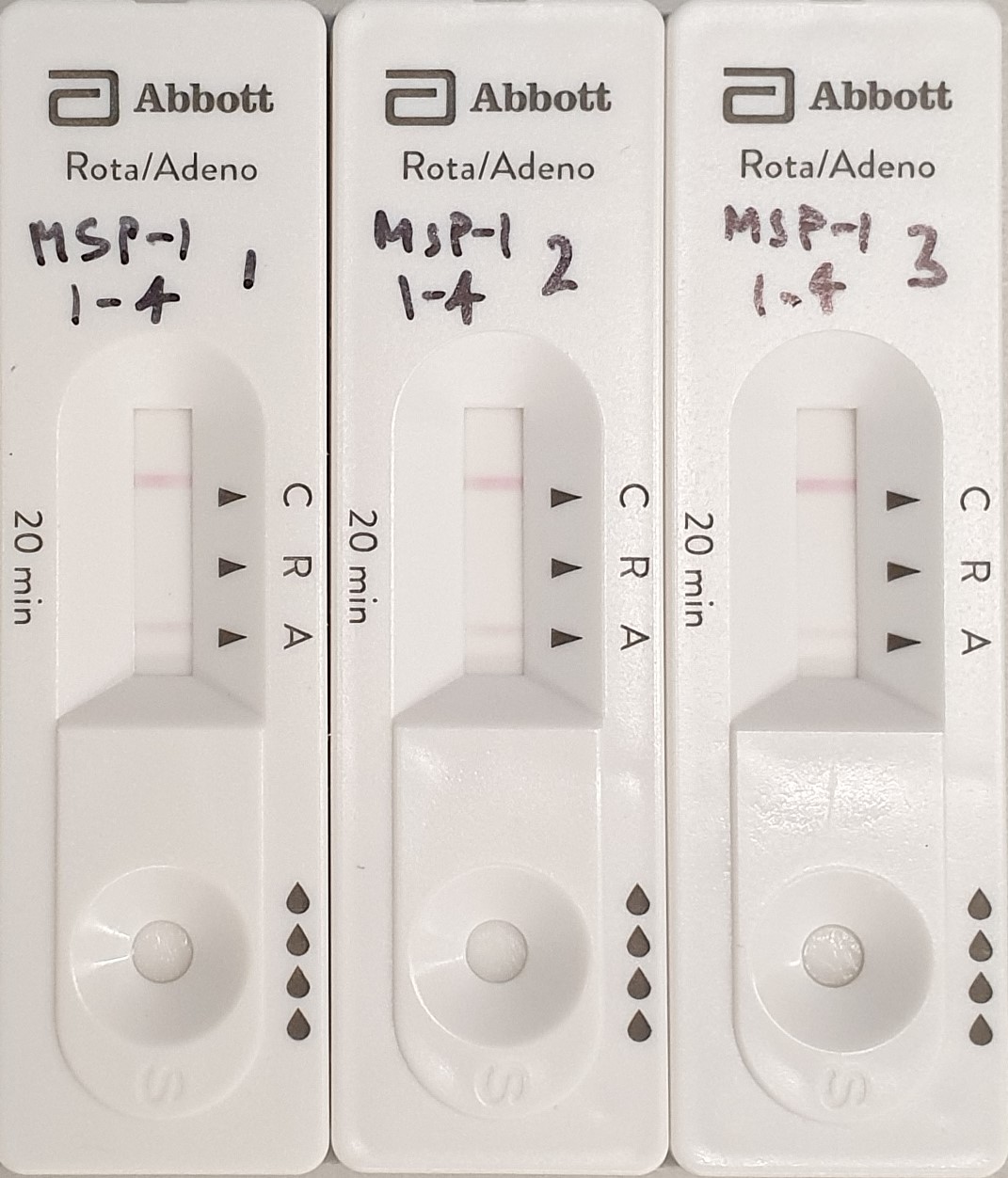

Supplement: Supplementary data [file EMS207833-supplement-Supplementary_data.zip › Initial assessment/MSP1/MSP1_Batch 1_Vial 4.jpg]

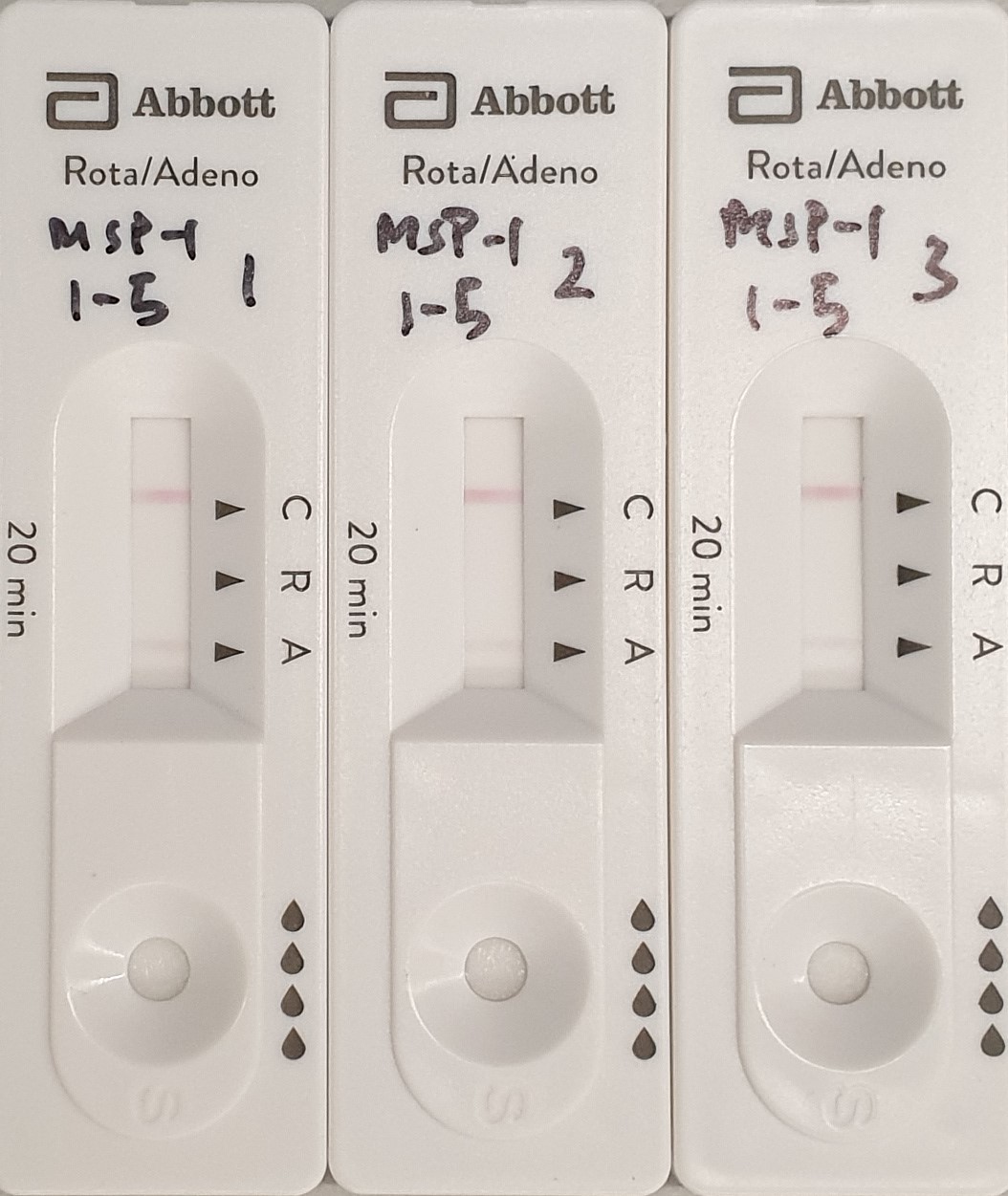

Supplement: Supplementary data [file EMS207833-supplement-Supplementary_data.zip › Initial assessment/MSP1/MSP1_Batch 1_Vial 5.jpg]

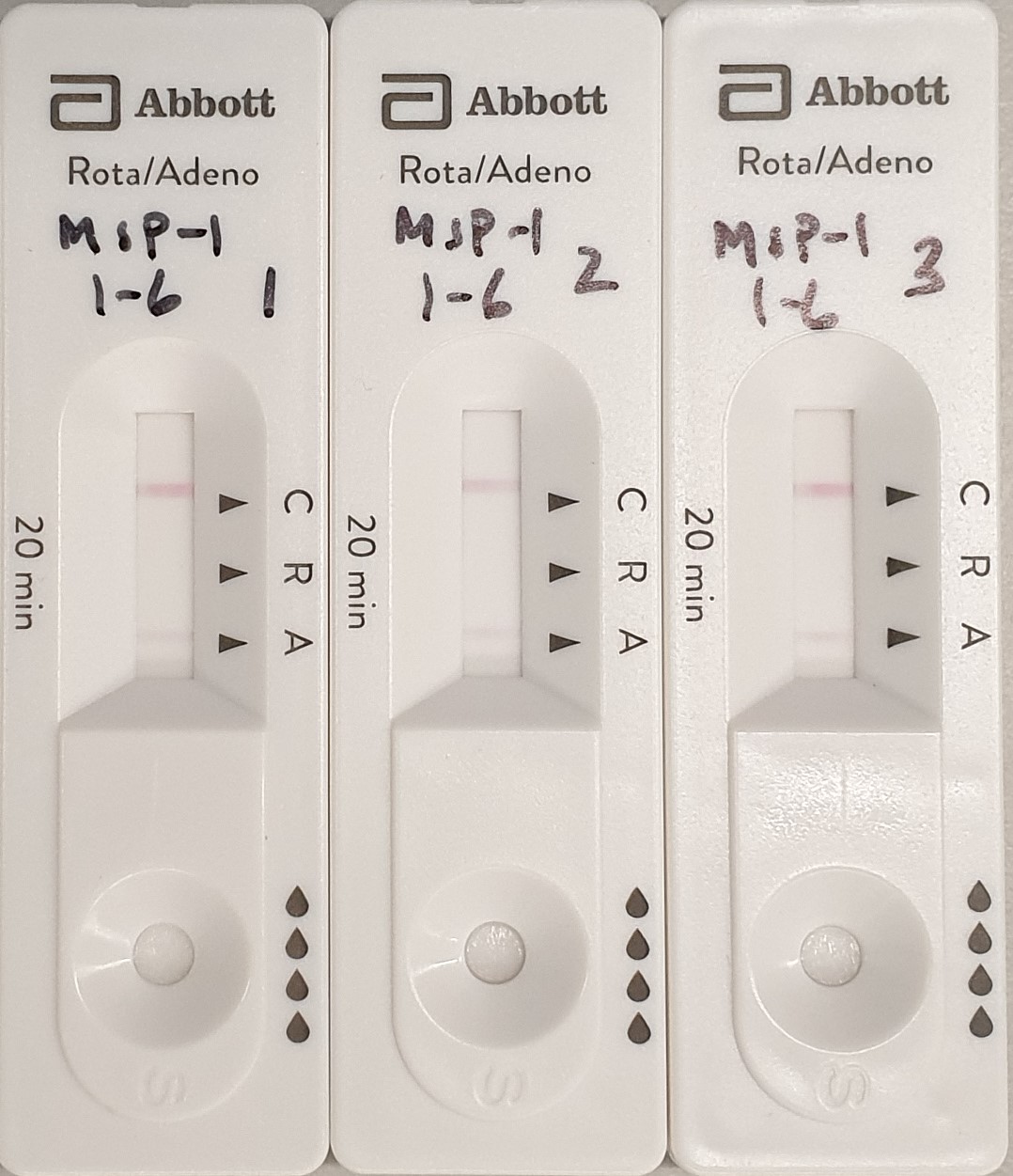

Supplement: Supplementary data [file EMS207833-supplement-Supplementary_data.zip › Initial assessment/MSP1/MSP1_Batch 1_Vial 6.jpg]

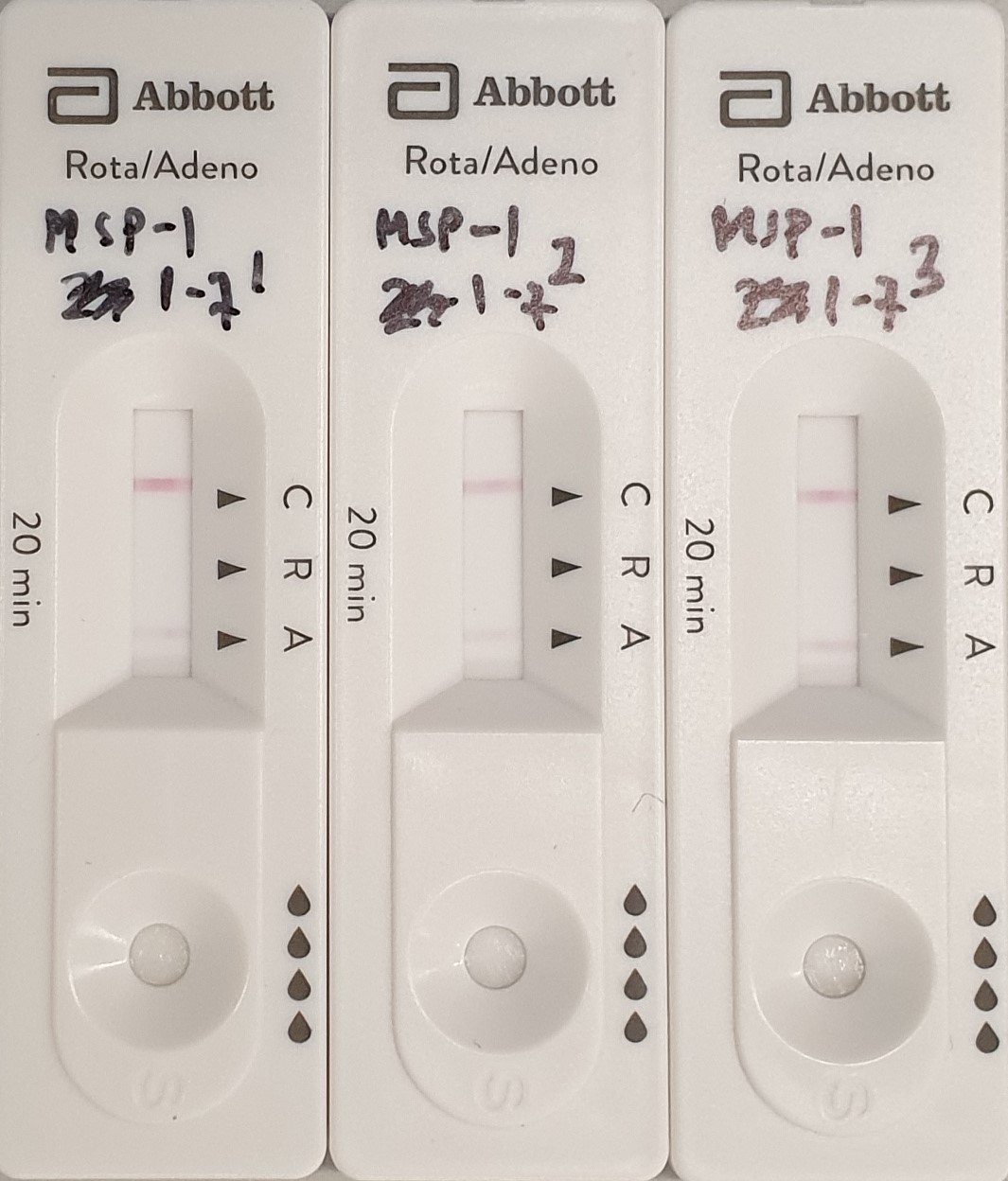

Supplement: Supplementary data [file EMS207833-supplement-Supplementary_data.zip › Initial assessment/MSP1/MSP1_Batch 1_Vial 7.jpg]

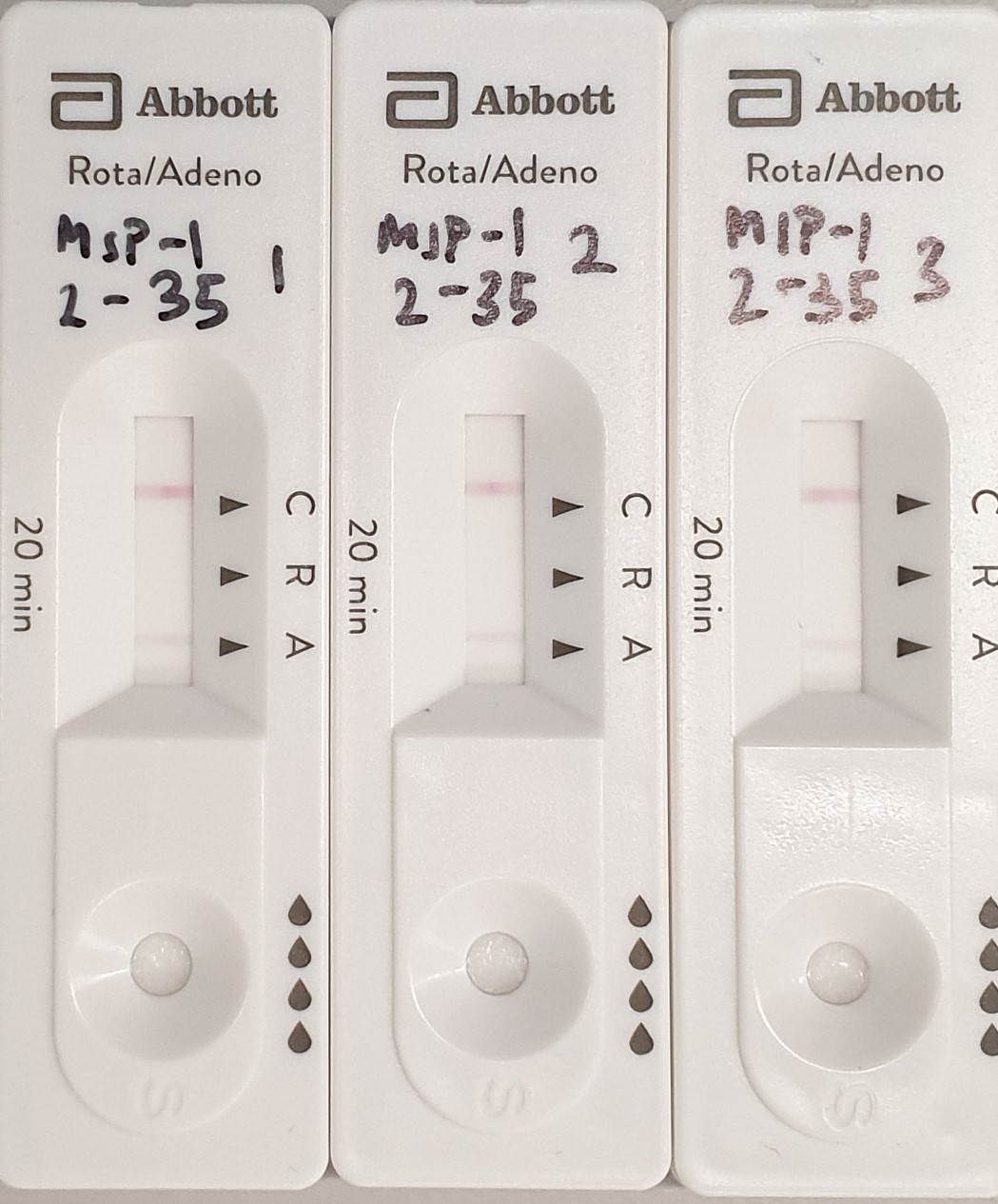

Supplement: Supplementary data [file EMS207833-supplement-Supplementary_data.zip › Initial assessment/MSP1/MSP1_Batch 2_Vial 35.jpg]

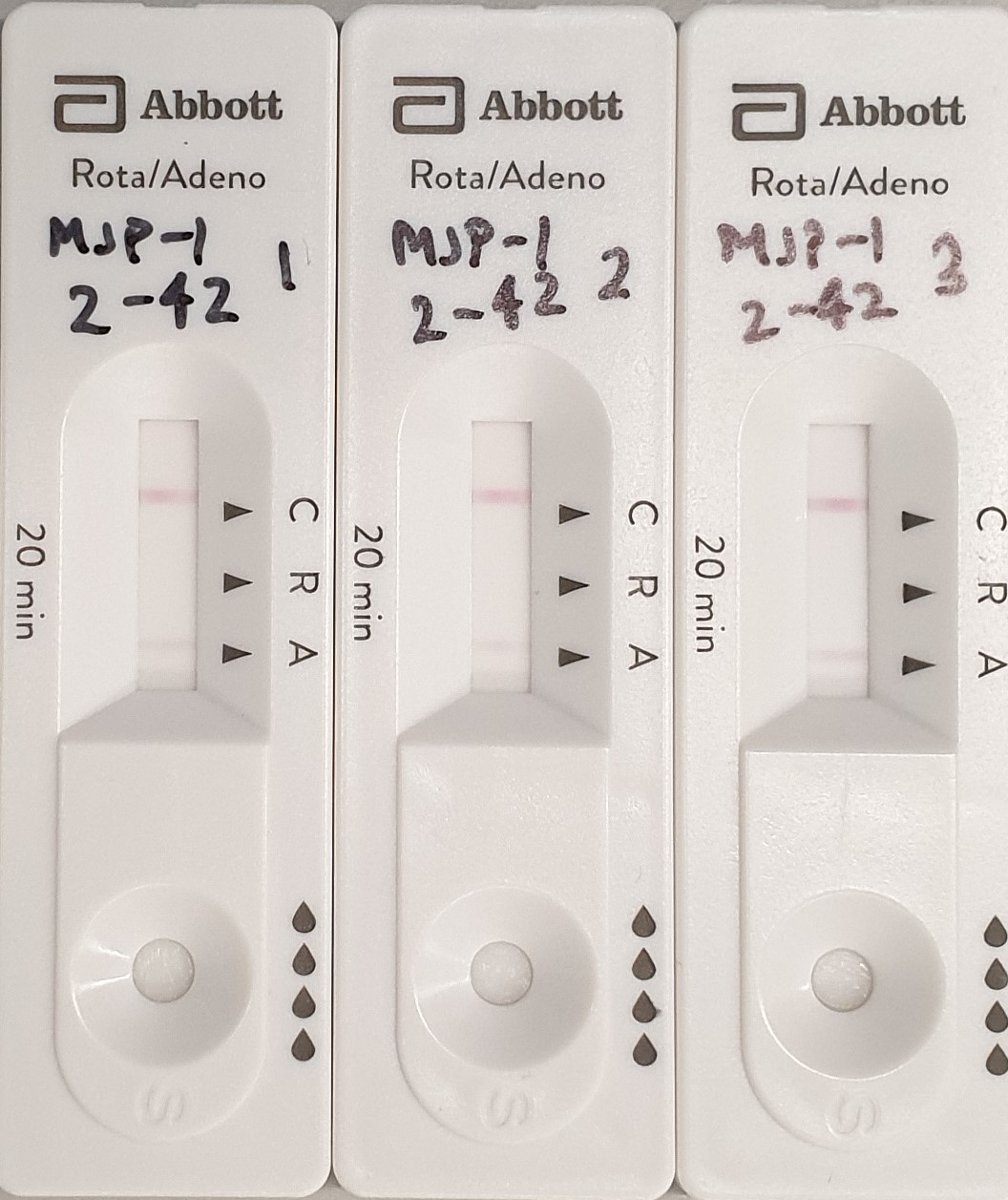

Supplement: Supplementary data [file EMS207833-supplement-Supplementary_data.zip › Initial assessment/MSP1/MSP1_Batch 2_Vial 42.jpg]

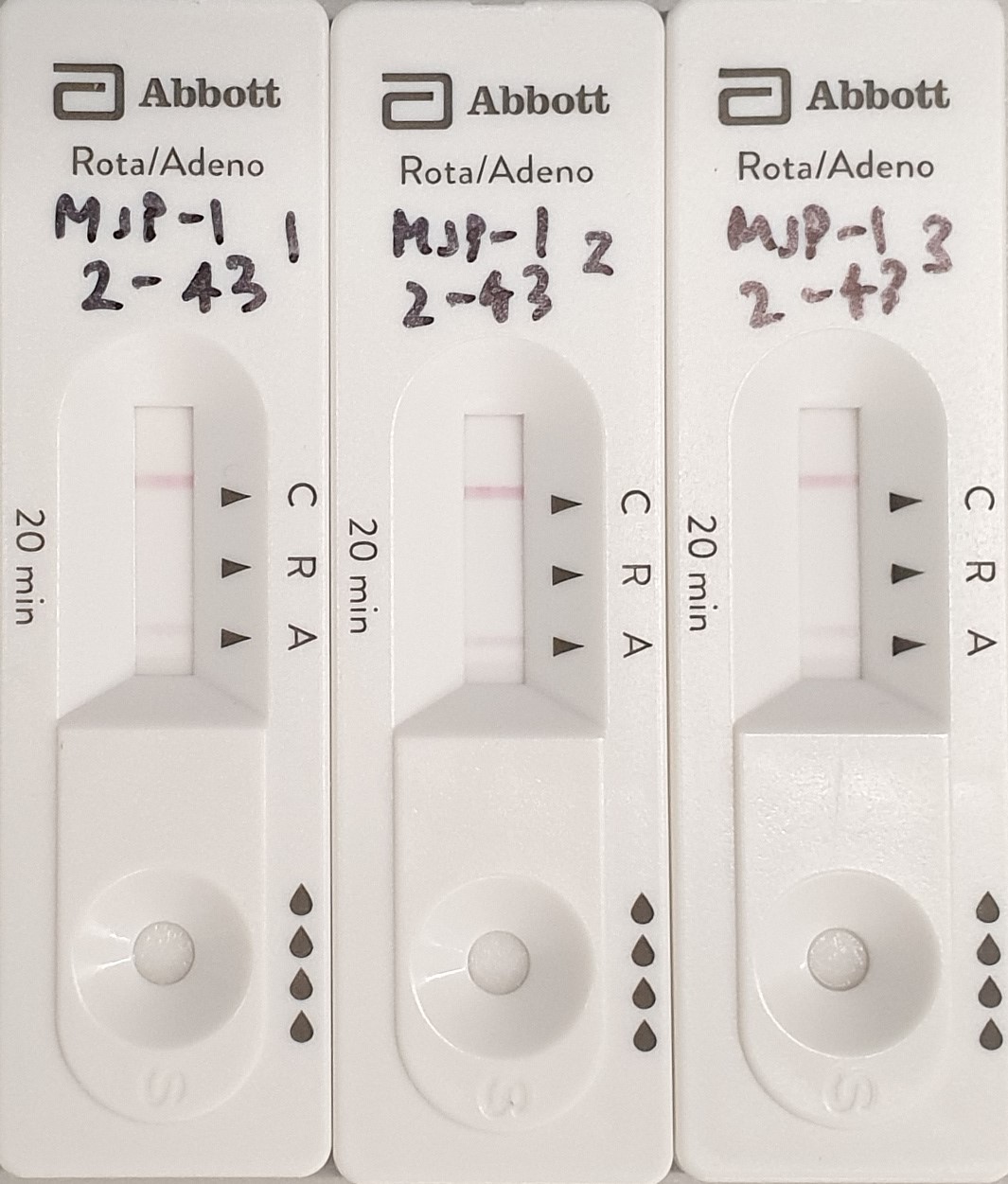

Supplement: Supplementary data [file EMS207833-supplement-Supplementary_data.zip › Initial assessment/MSP1/MSP1_Batch 2_Vial 43.jpg]

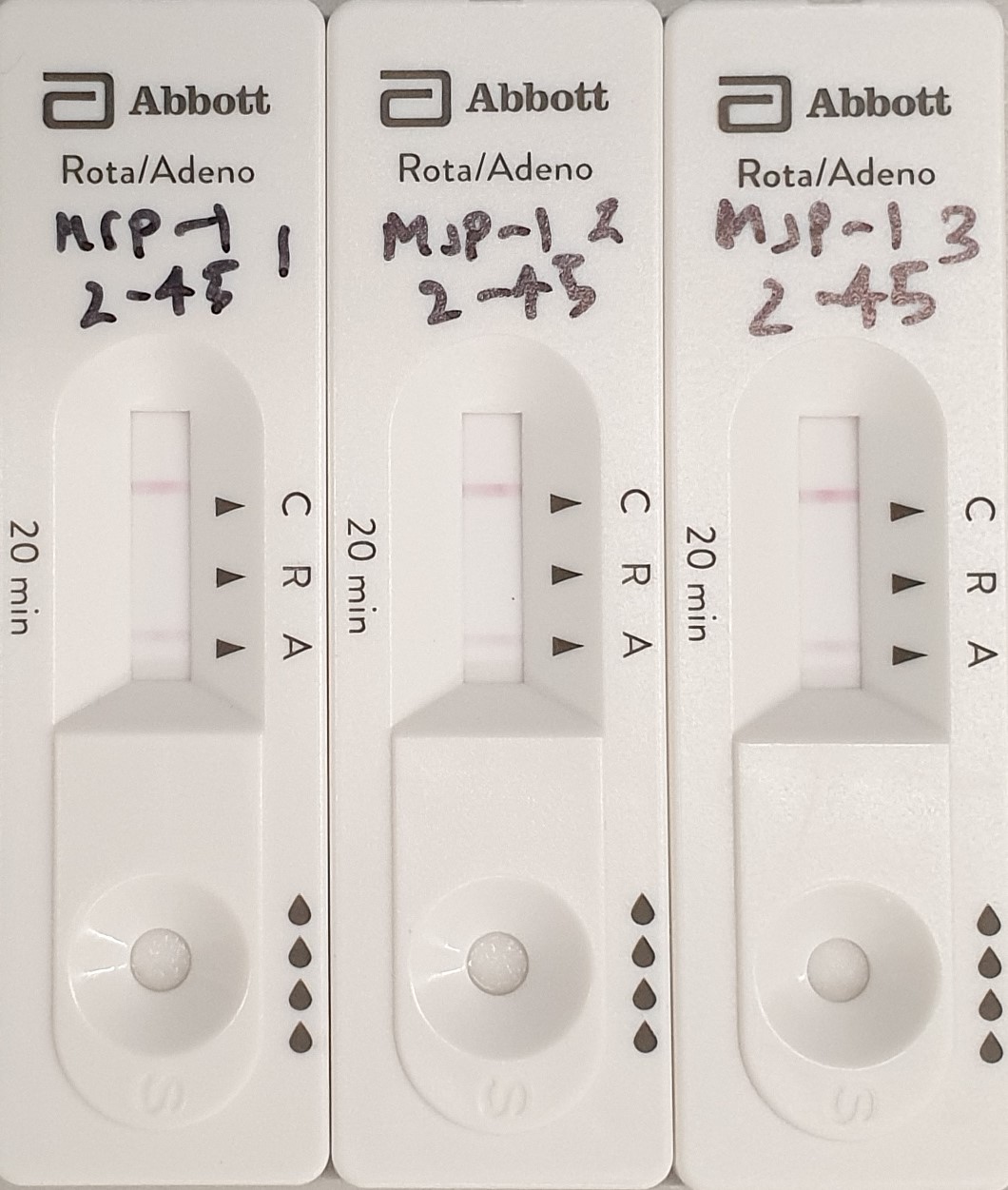

Supplement: Supplementary data [file EMS207833-supplement-Supplementary_data.zip › Initial assessment/MSP1/MSP1_Batch 2_Vial 45.jpg]

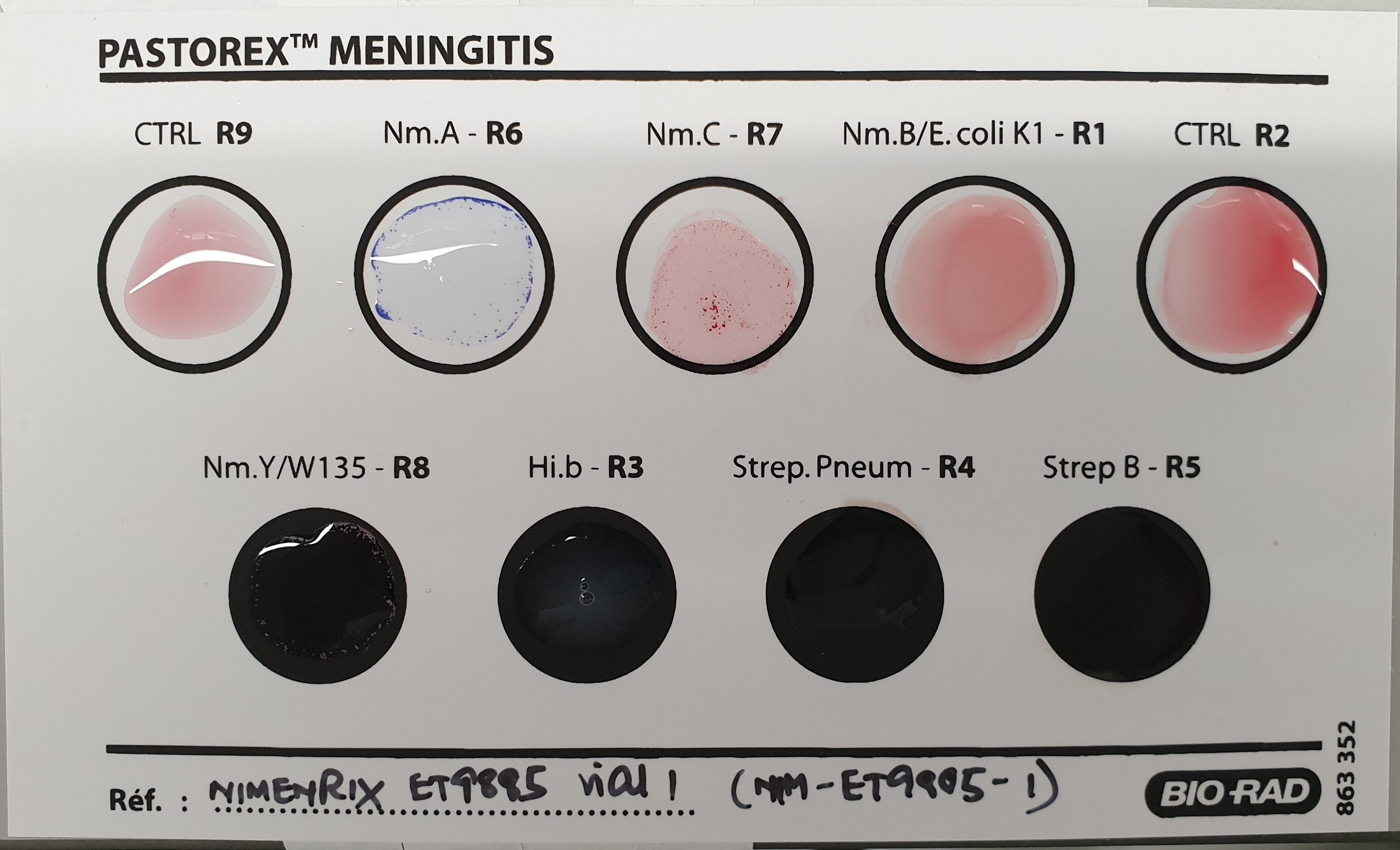

Supplement: Supplementary data [file EMS207833-supplement-Supplementary_data.zip › Initial assessment/Nimenrix/Nimenrix_Batch 1_Vial 1.jpg]

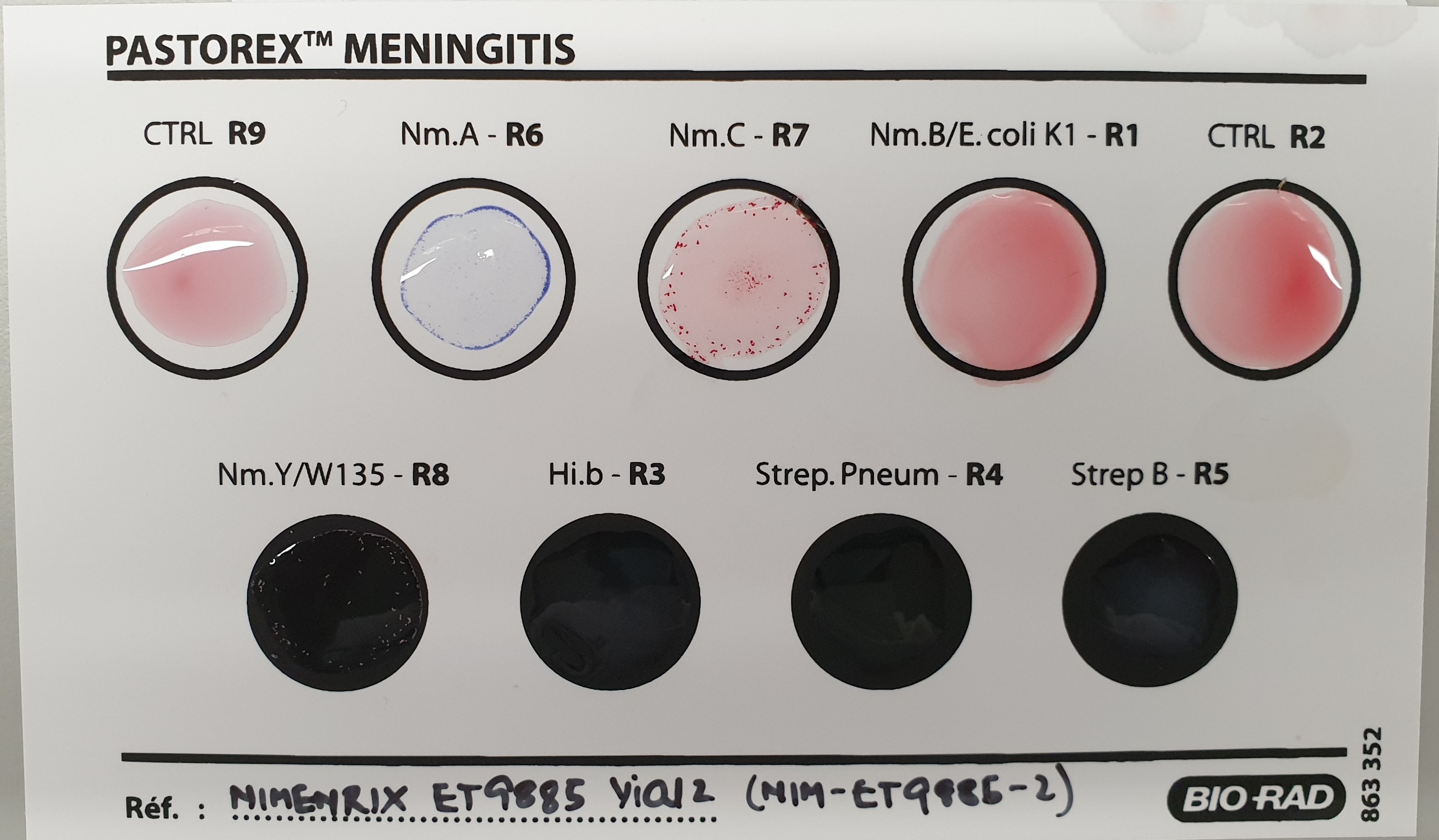

Supplement: Supplementary data [file EMS207833-supplement-Supplementary_data.zip › Initial assessment/Nimenrix/Nimenrix_Batch 1_Vial 2.jpg]

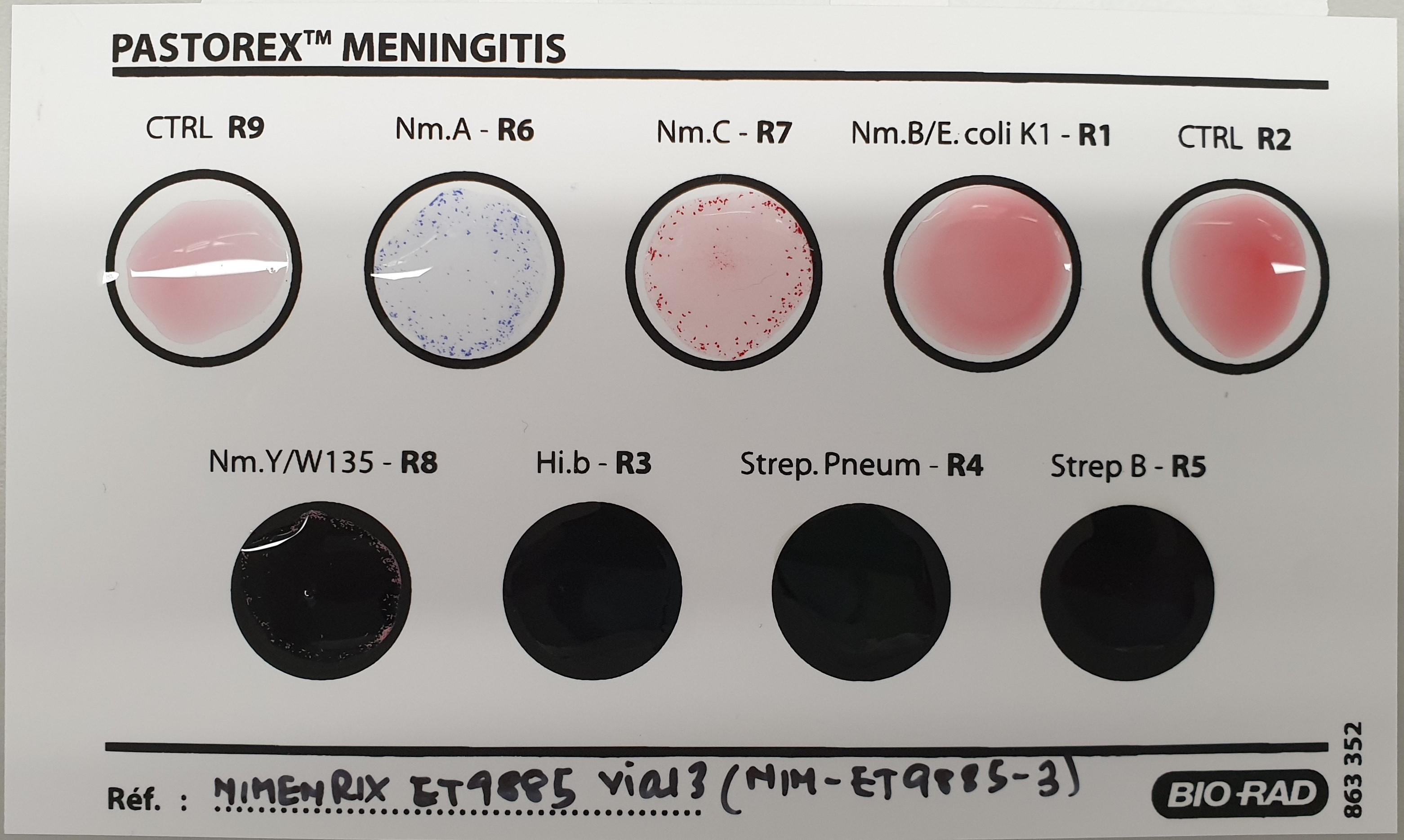

Supplement: Supplementary data [file EMS207833-supplement-Supplementary_data.zip › Initial assessment/Nimenrix/Nimenrix_Batch 1_Vial 3.jpg]

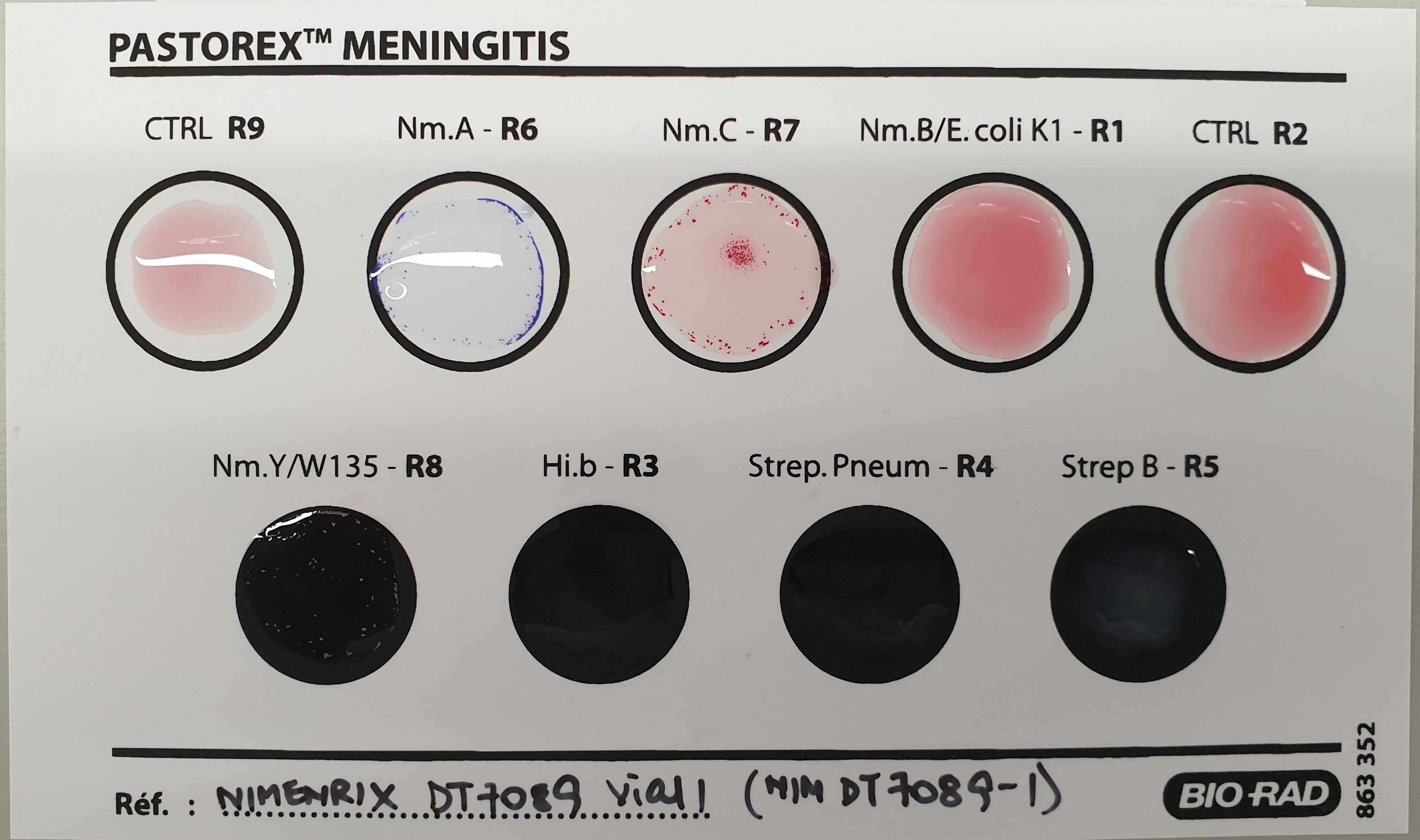

Supplement: Supplementary data [file EMS207833-supplement-Supplementary_data.zip › Initial assessment/Nimenrix/Nimenrix_Batch 2_Vial 1.jpg]

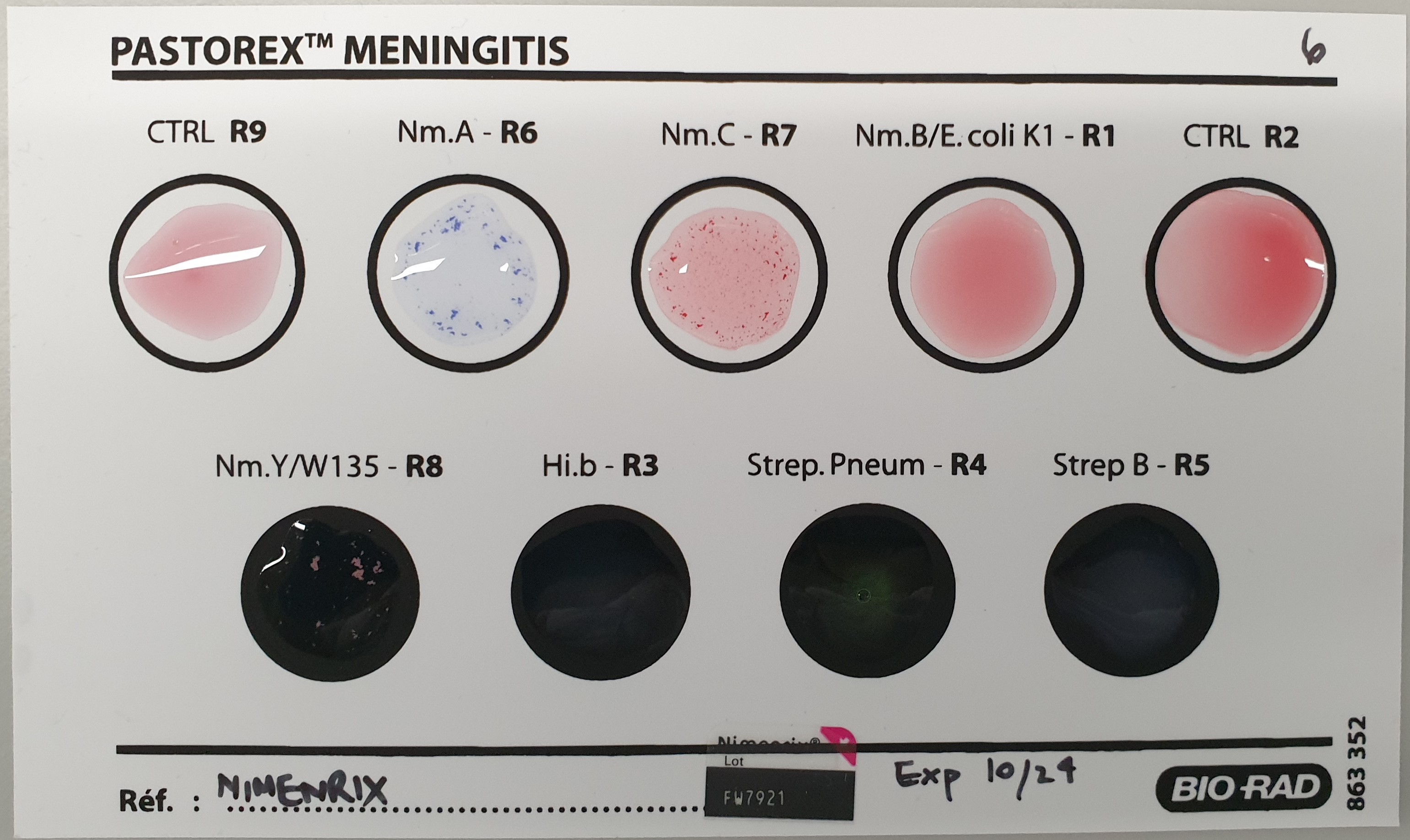

Supplement: Supplementary data [file EMS207833-supplement-Supplementary_data.zip › Initial assessment/Nimenrix/Nimenrix_Batch 3_Vial 1.jpg]

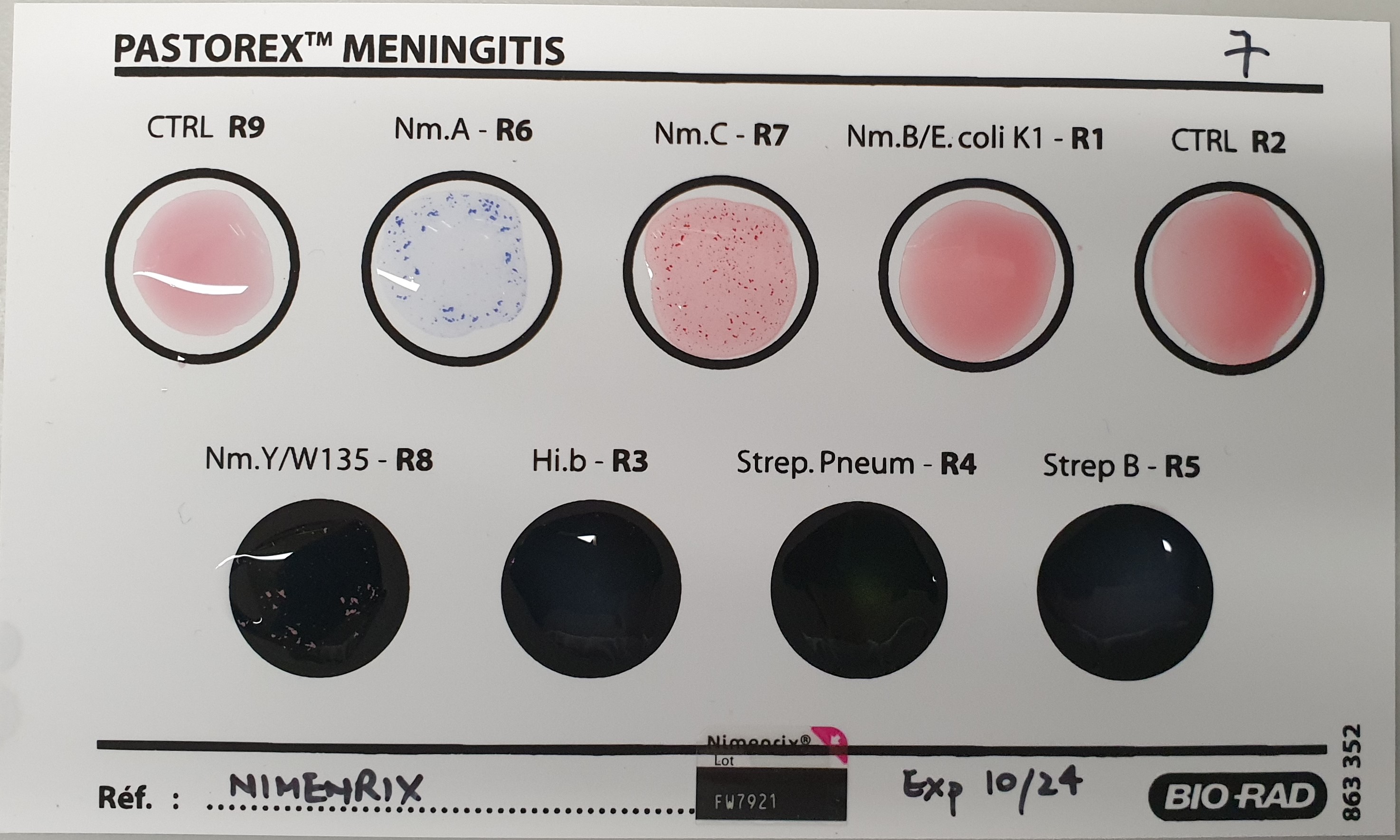

Supplement: Supplementary data [file EMS207833-supplement-Supplementary_data.zip › Initial assessment/Nimenrix/Nimenrix_Batch 3_Vial 2.jpg]

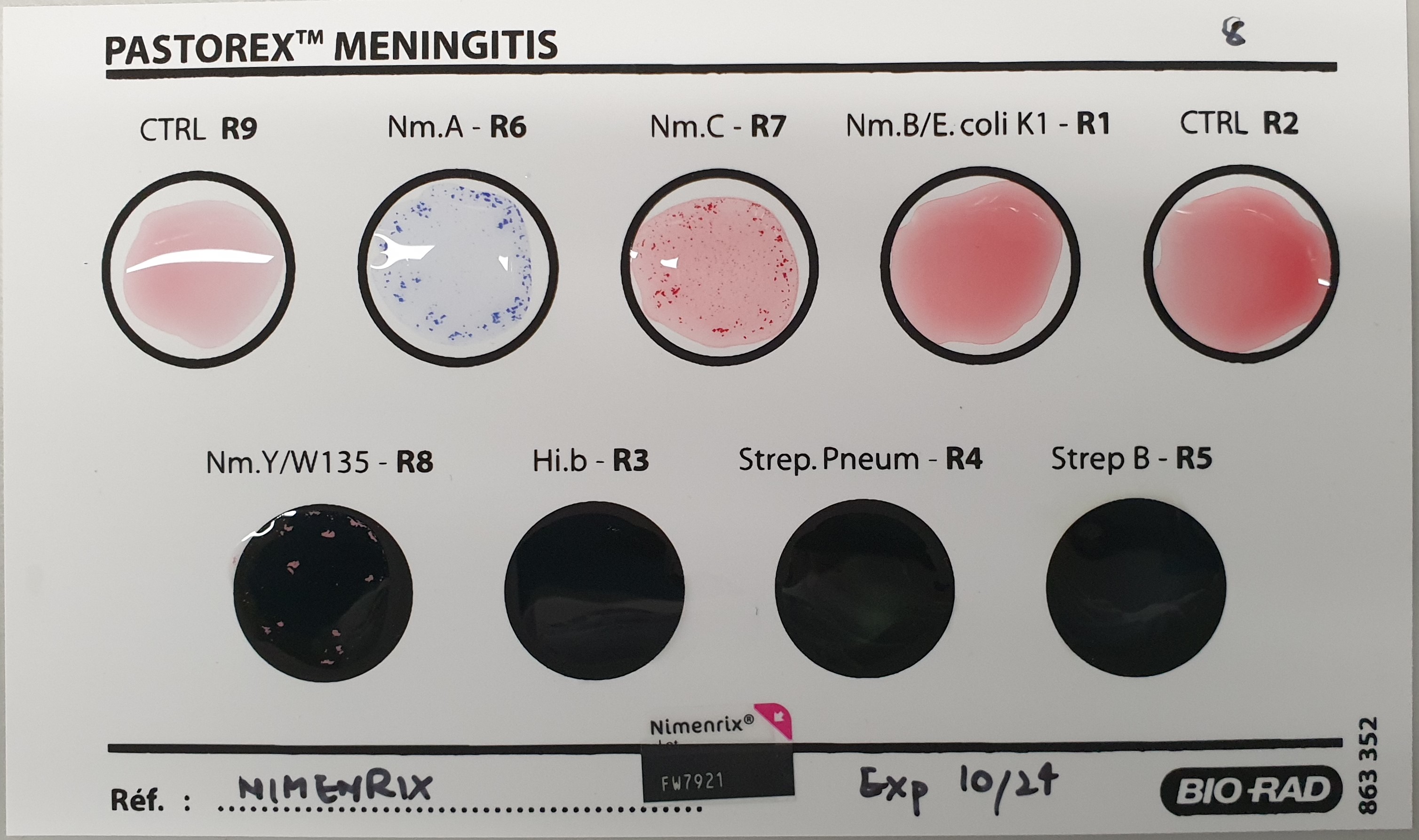

Supplement: Supplementary data [file EMS207833-supplement-Supplementary_data.zip › Initial assessment/Nimenrix/Nimenrix_Batch 3_Vial 3.jpg]

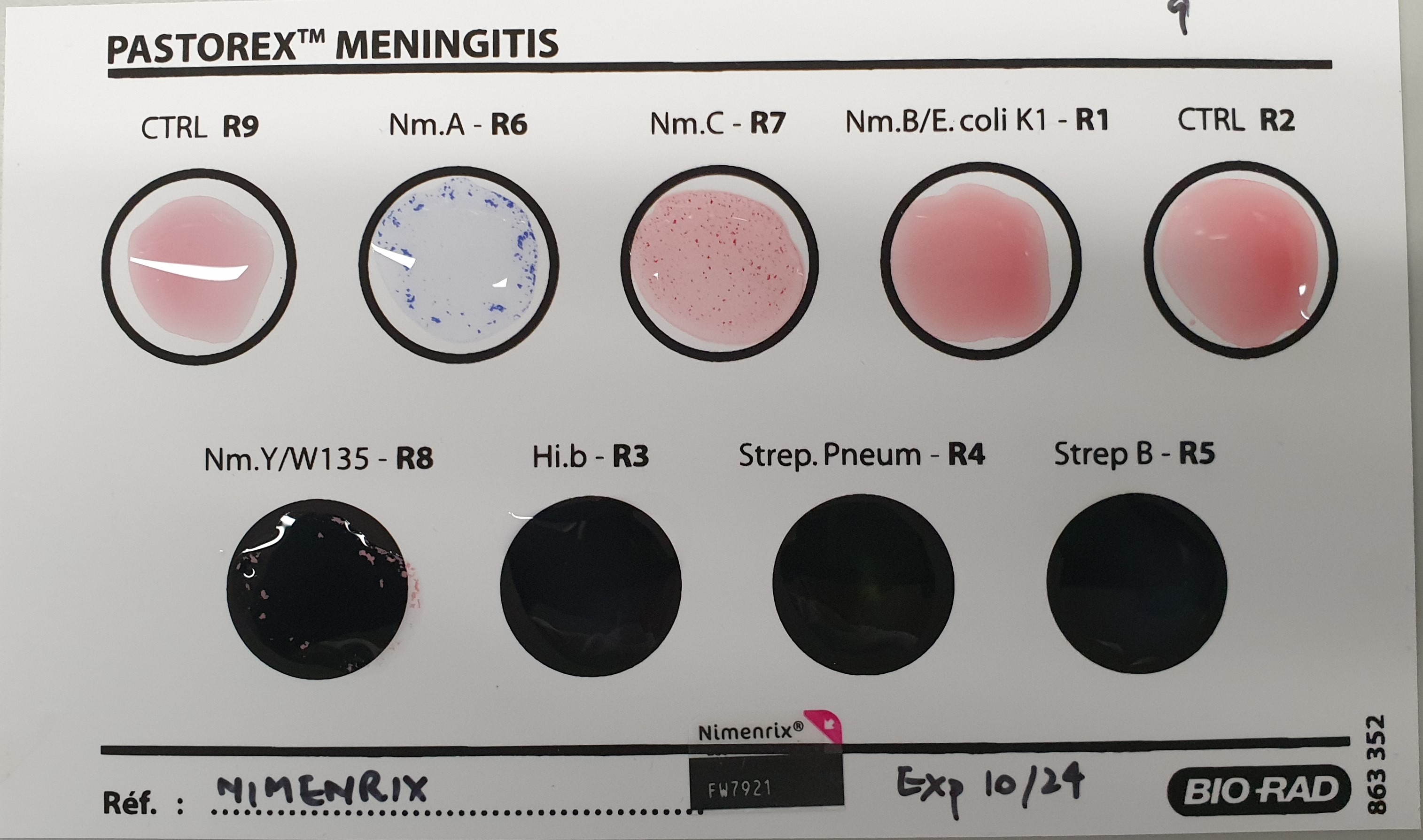

Supplement: Supplementary data [file EMS207833-supplement-Supplementary_data.zip › Initial assessment/Nimenrix/Nimenrix_Batch 3_Vial 4.jpg]

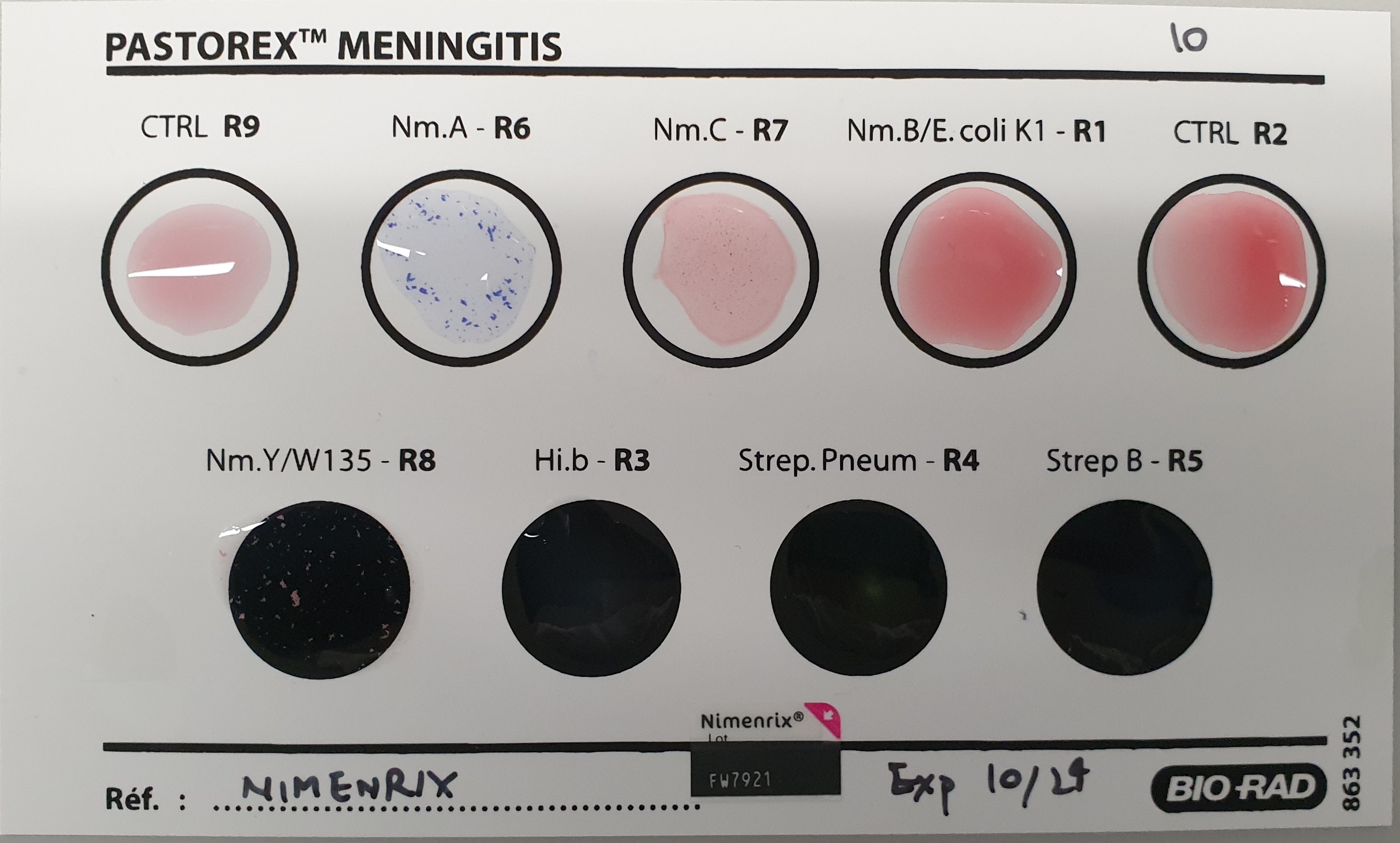

Supplement: Supplementary data [file EMS207833-supplement-Supplementary_data.zip › Initial assessment/Nimenrix/Nimenrix_Batch 3_Vial 5.jpg]

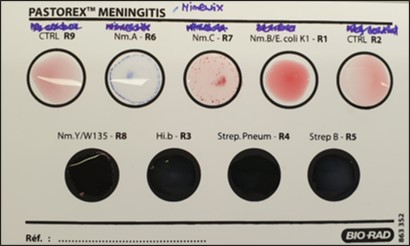

Supplement: Supplementary data [file EMS207833-supplement-Supplementary_data.zip › Initial assessment/Nimenrix/Nimenrix_Batch 4_Vial 1.jpg]

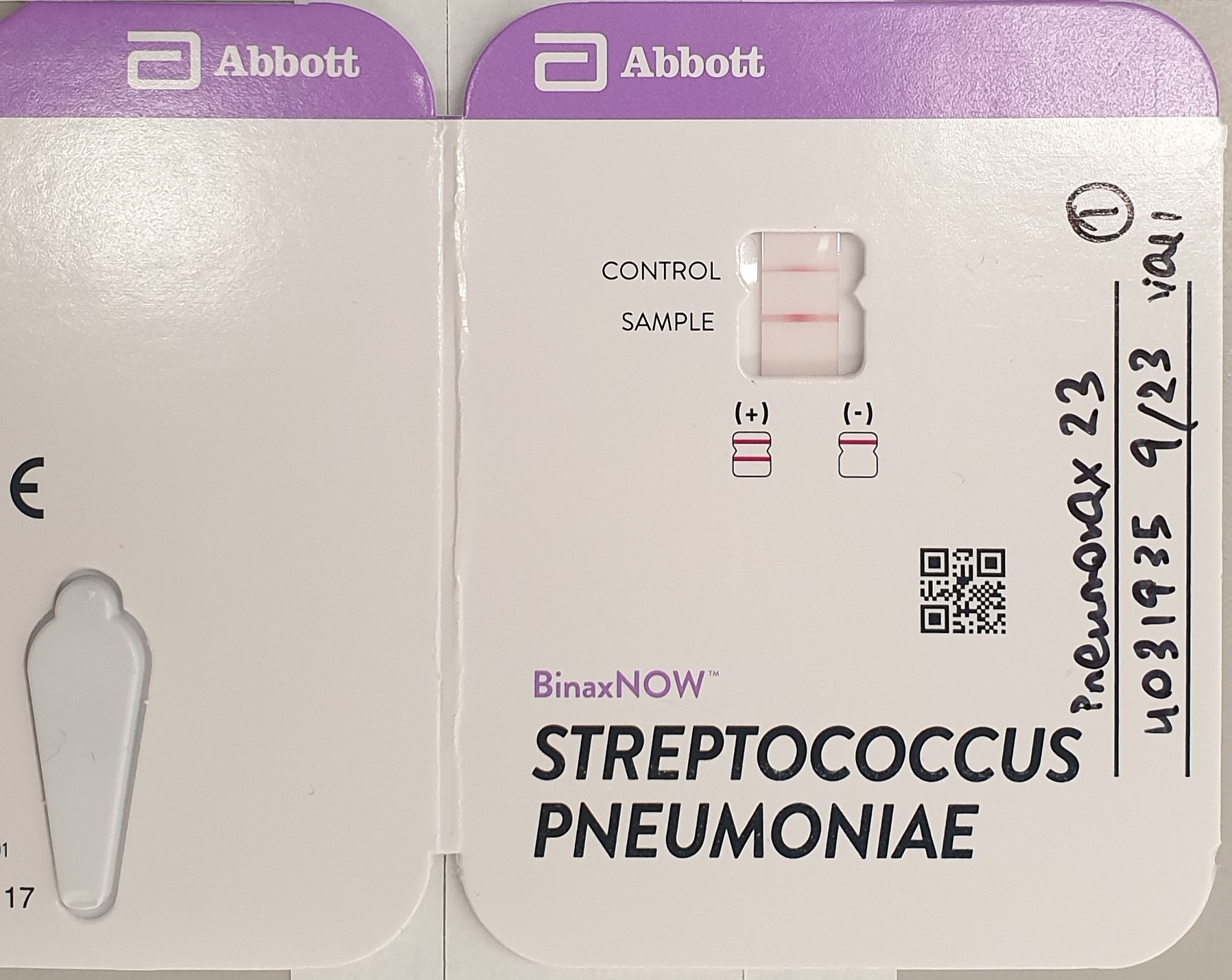

Supplement: Supplementary data [file EMS207833-supplement-Supplementary_data.zip › Initial assessment/Pneumovax-23/Pneumovax23_Batch1_Vial1_1.jpg]

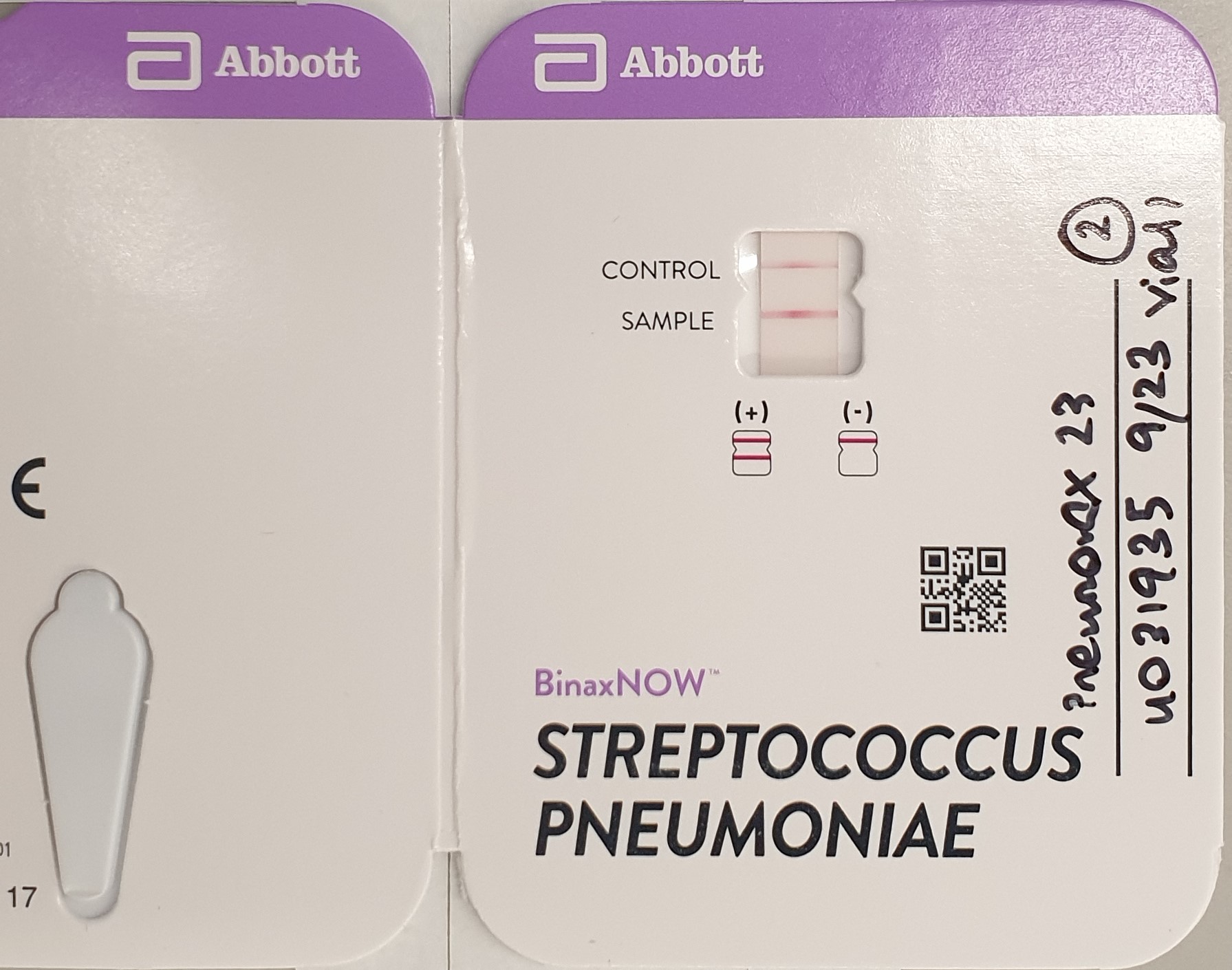

Supplement: Supplementary data [file EMS207833-supplement-Supplementary_data.zip › Initial assessment/Pneumovax-23/Pneumovax23_Batch1_Vial1_2.jpg]

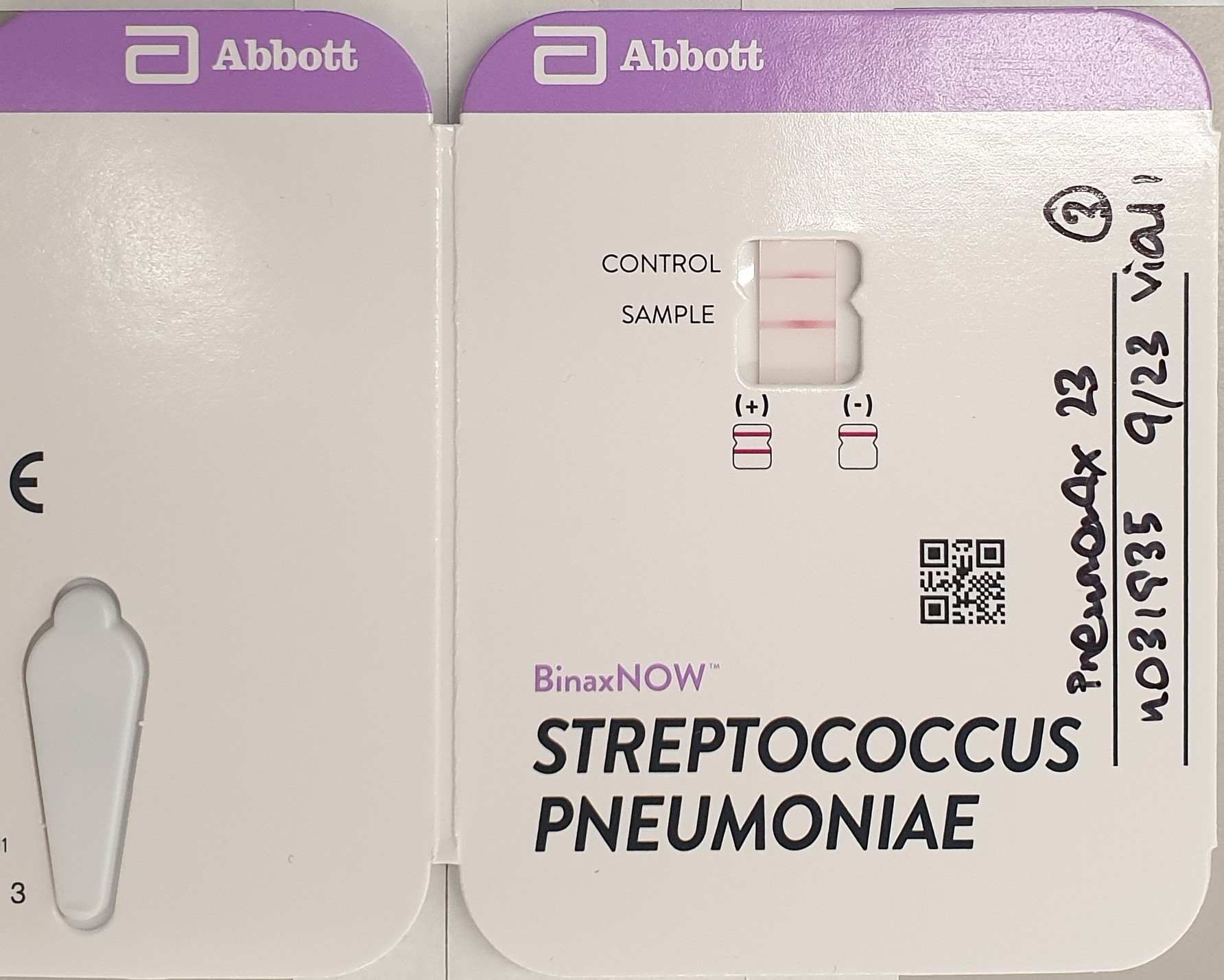

Supplement: Supplementary data [file EMS207833-supplement-Supplementary_data.zip › Initial assessment/Pneumovax-23/Pneumovax23_Batch1_Vial1_3.jpg]

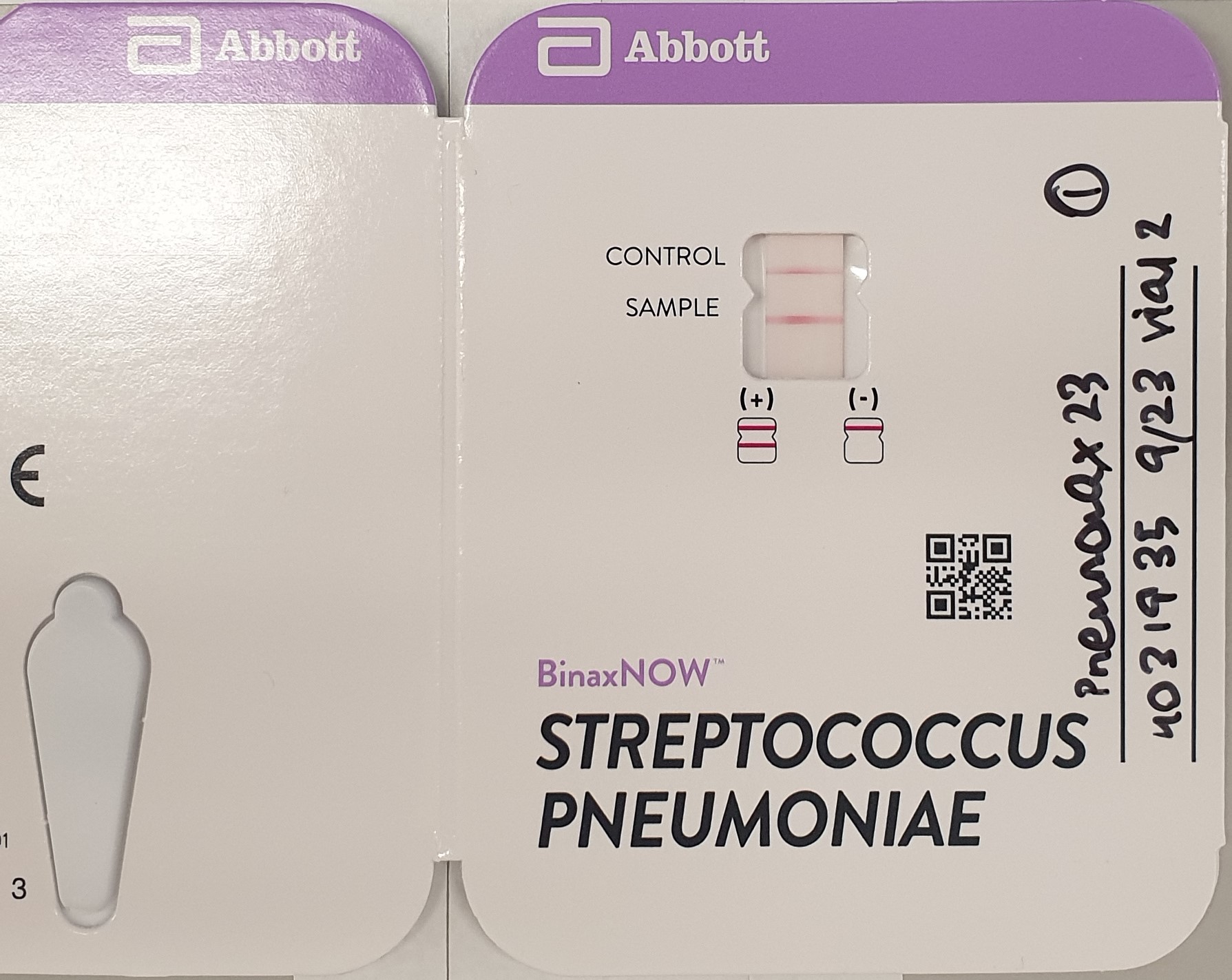

Supplement: Supplementary data [file EMS207833-supplement-Supplementary_data.zip › Initial assessment/Pneumovax-23/Pneumovax23_Batch1_Vial2_1.jpg]

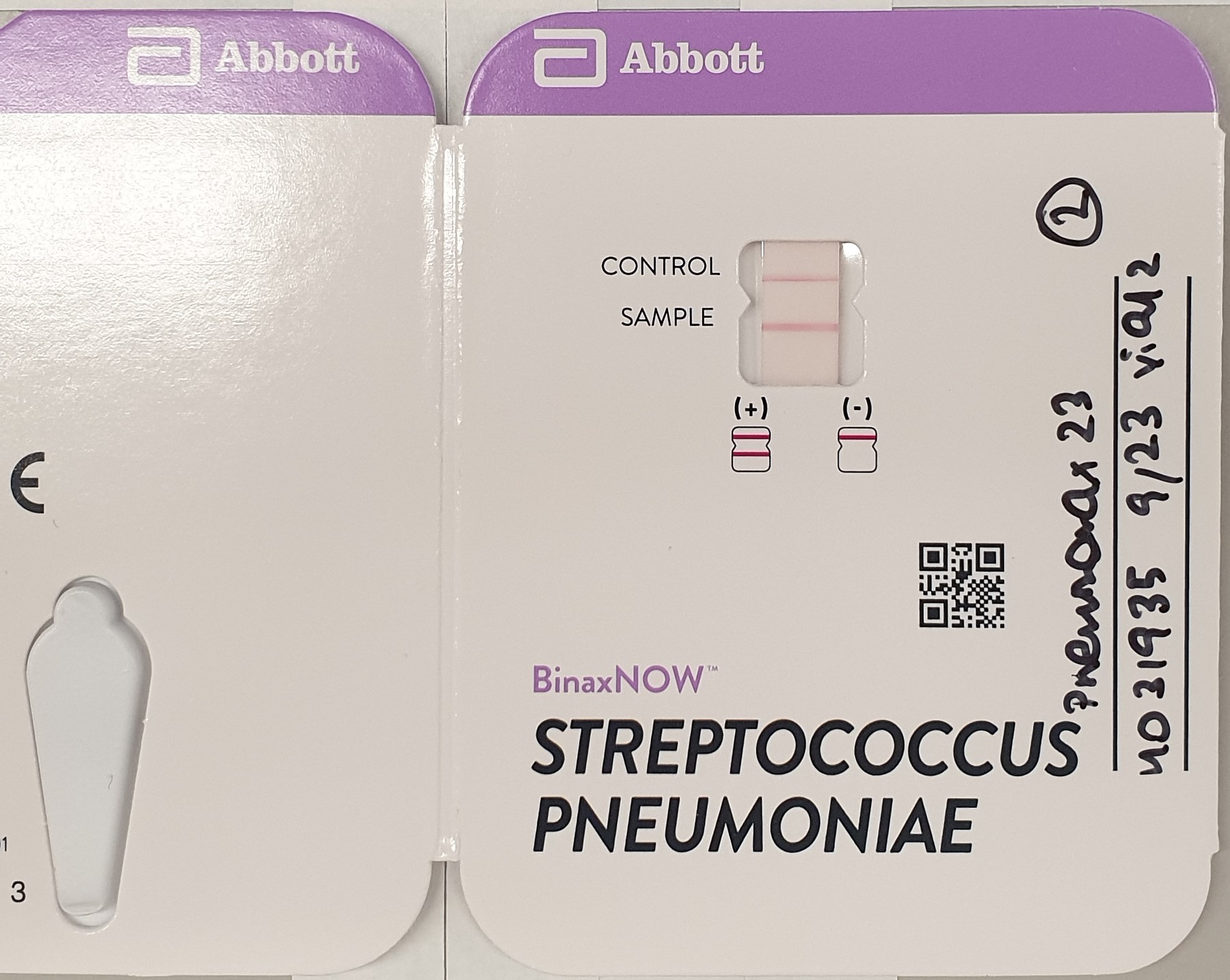

Supplement: Supplementary data [file EMS207833-supplement-Supplementary_data.zip › Initial assessment/Pneumovax-23/Pneumovax23_Batch1_Vial2_2.jpg]

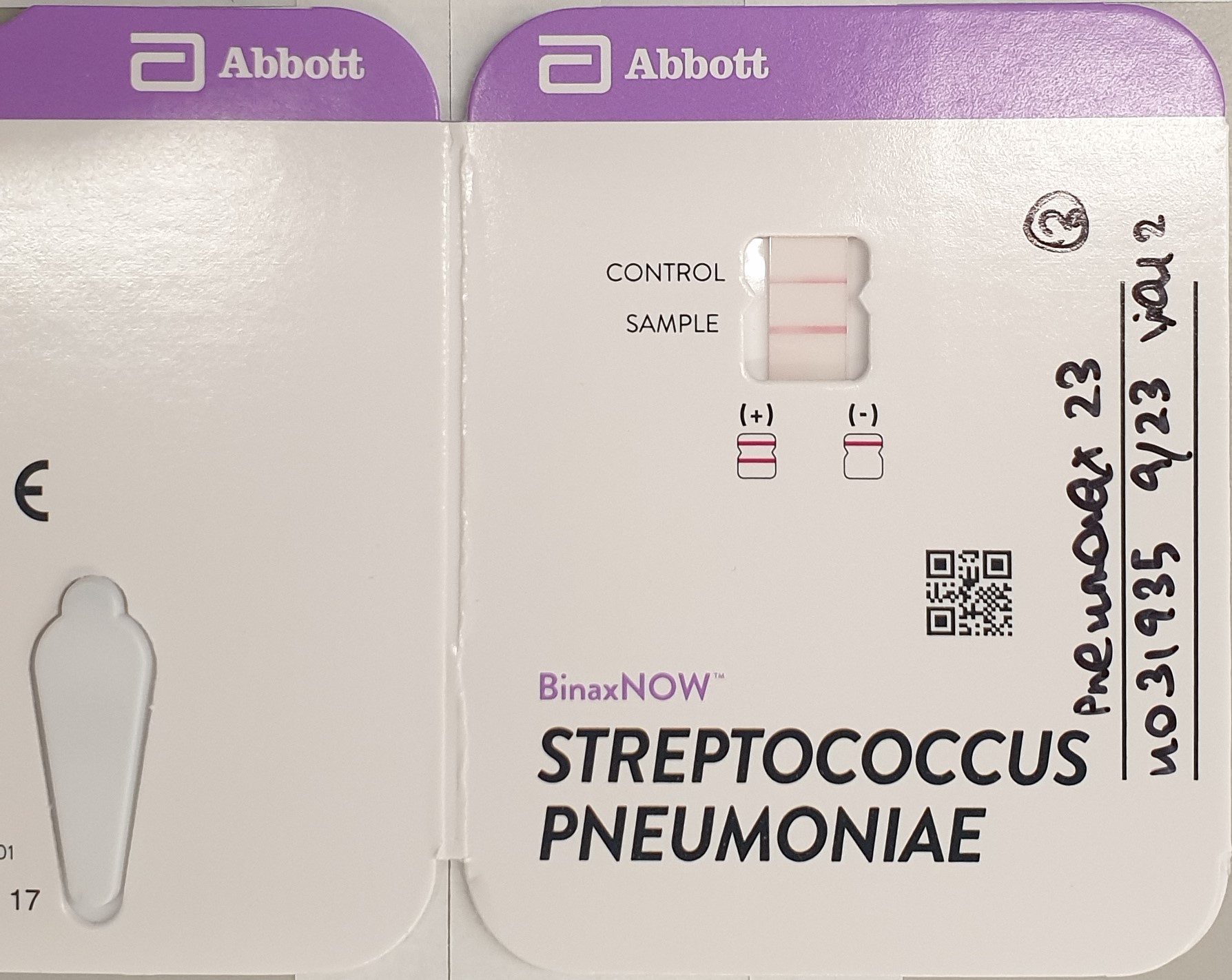

Supplement: Supplementary data [file EMS207833-supplement-Supplementary_data.zip › Initial assessment/Pneumovax-23/Pneumovax23_Batch1_Vial2_3.jpg]

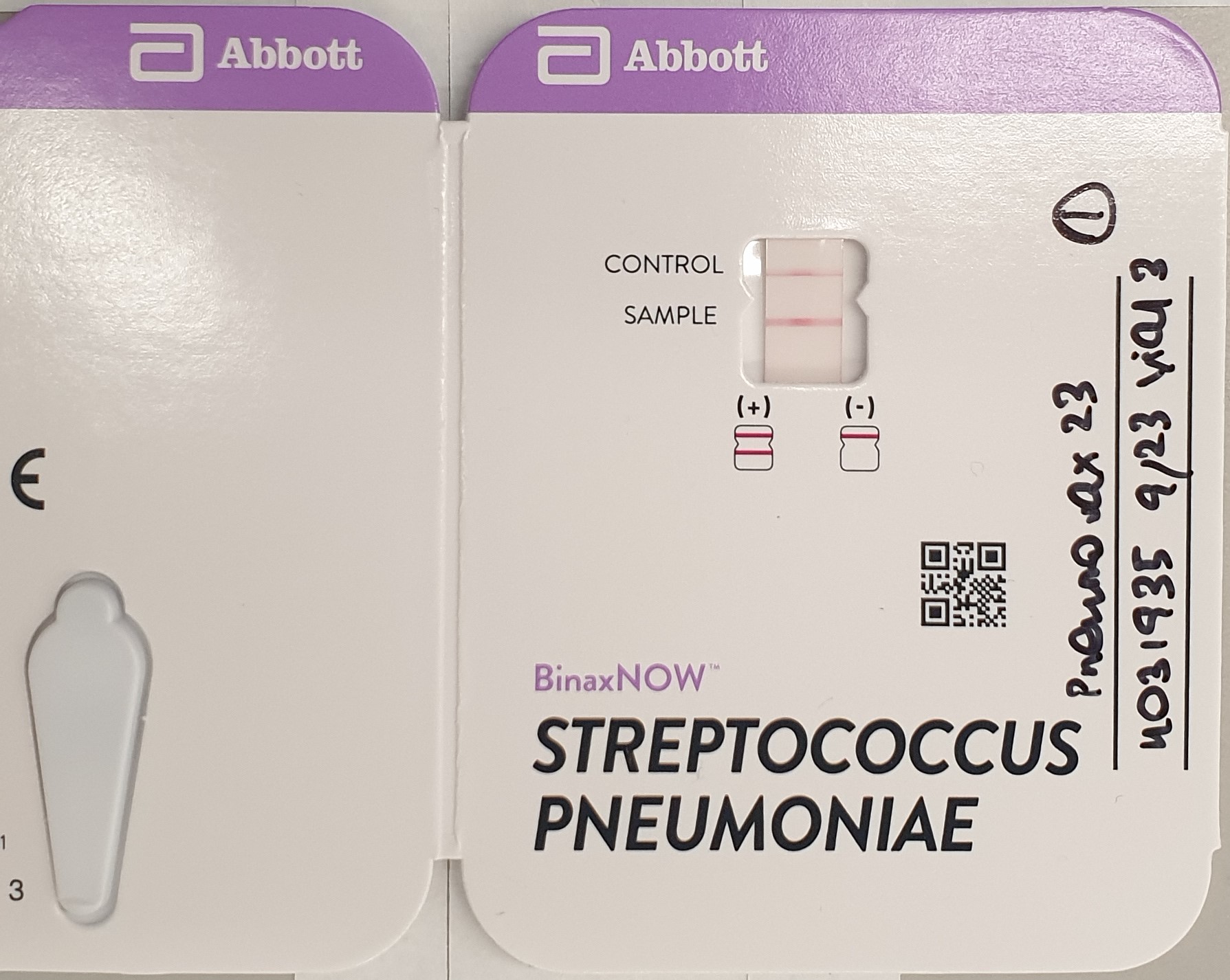

Supplement: Supplementary data [file EMS207833-supplement-Supplementary_data.zip › Initial assessment/Pneumovax-23/Pneumovax23_Batch1_Vial3_1.jpg]

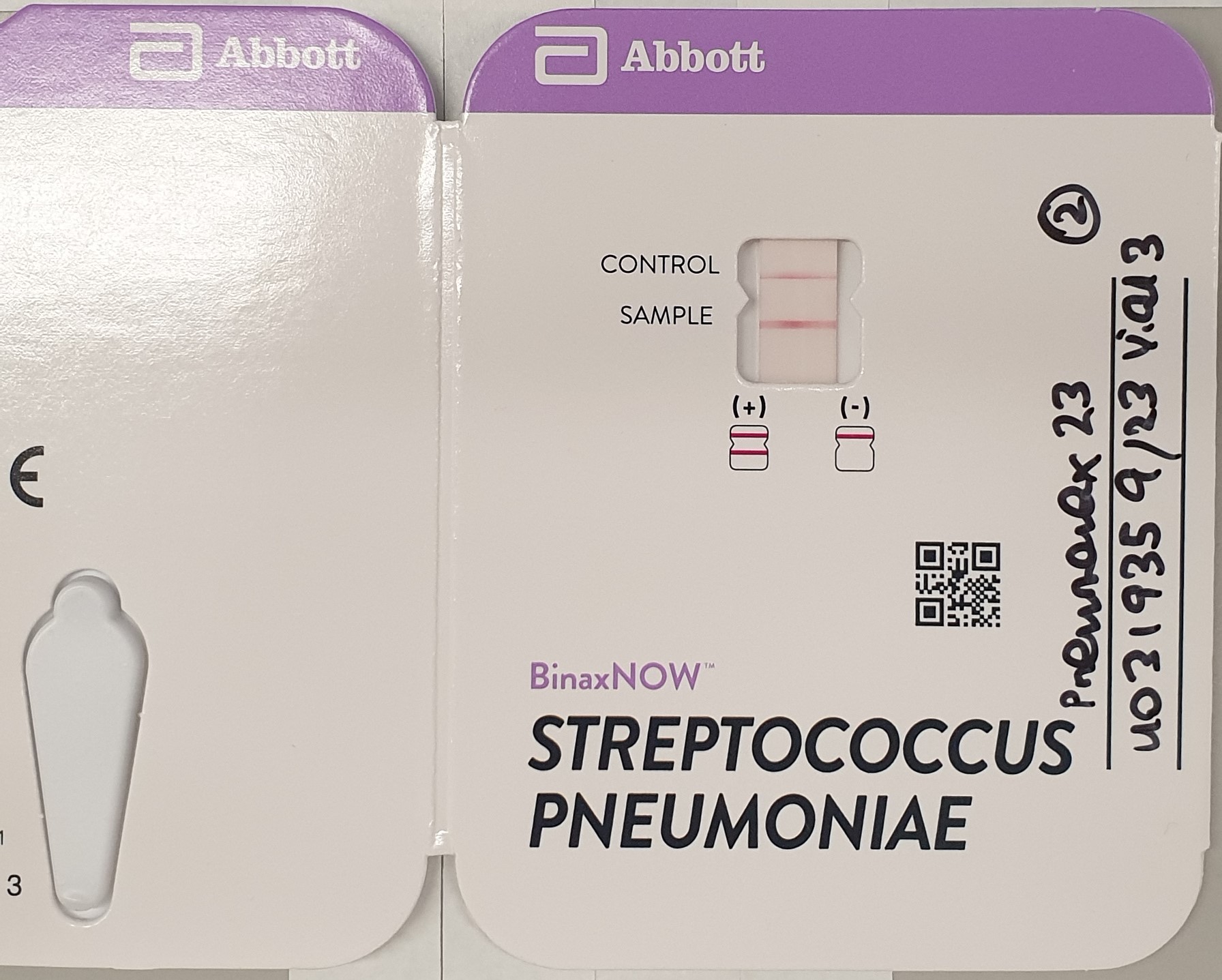

Supplement: Supplementary data [file EMS207833-supplement-Supplementary_data.zip › Initial assessment/Pneumovax-23/Pneumovax23_Batch1_Vial3_2.jpg]

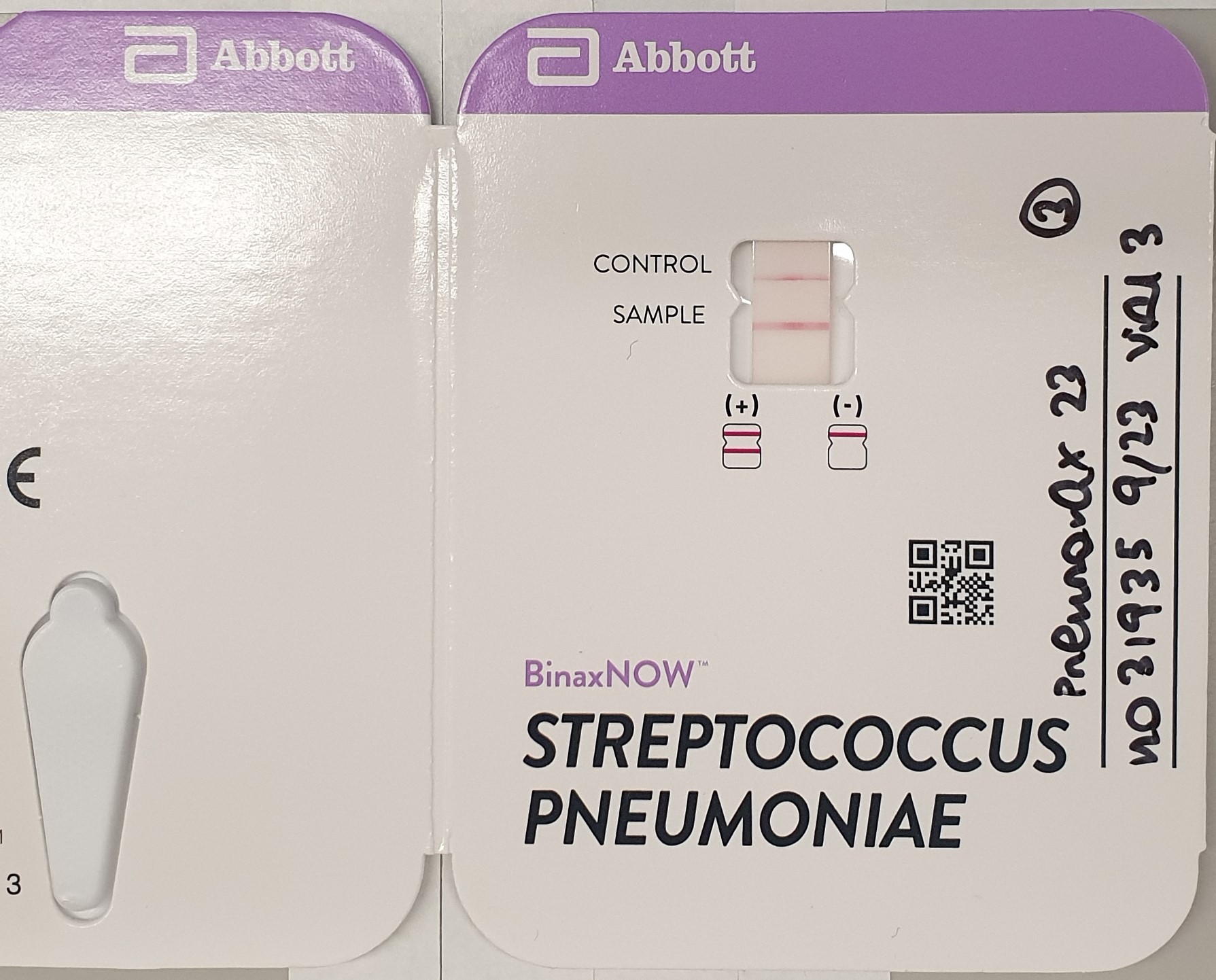

Supplement: Supplementary data [file EMS207833-supplement-Supplementary_data.zip › Initial assessment/Pneumovax-23/Pneumovax23_Batch1_Vial3_3.jpg]

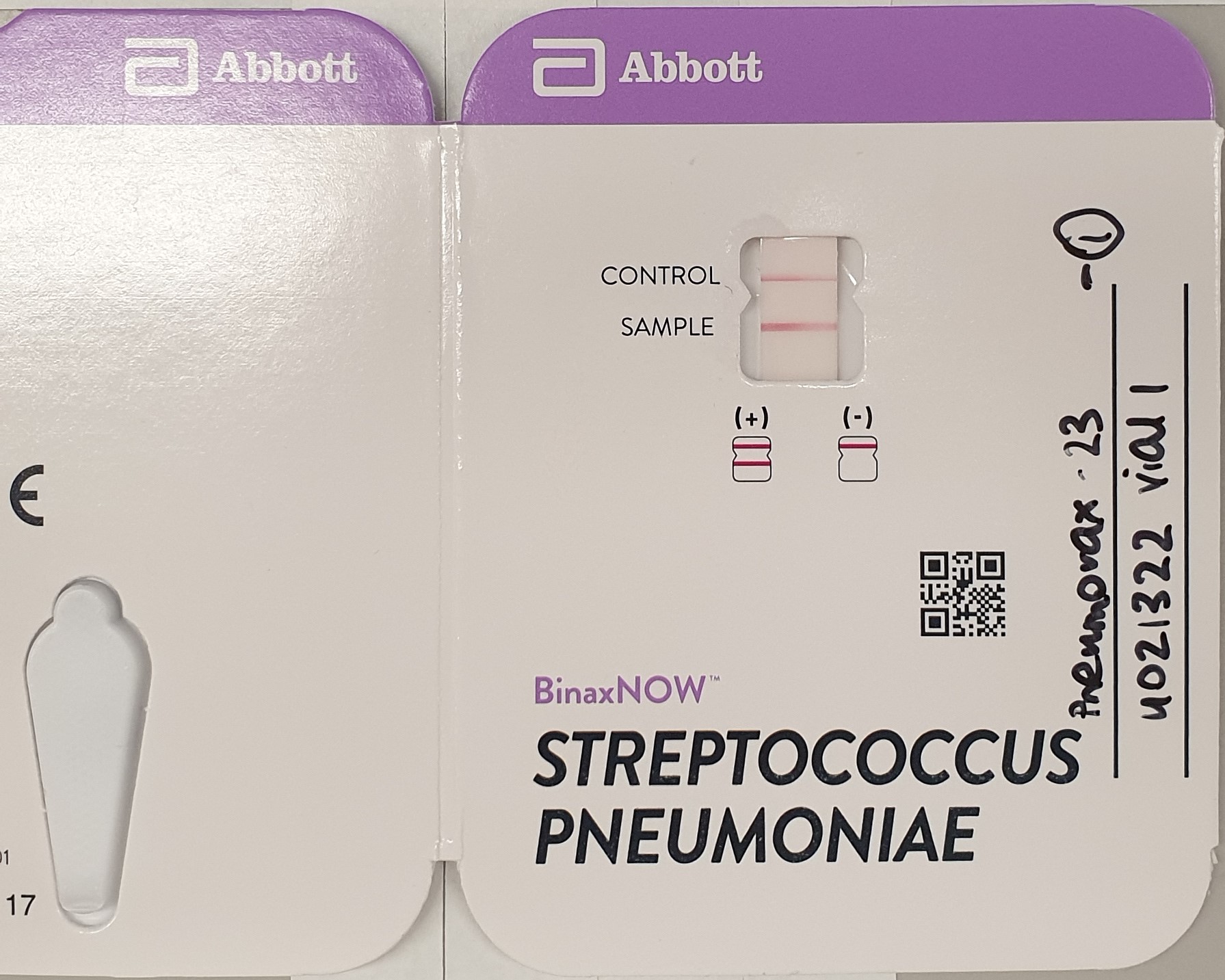

Supplement: Supplementary data [file EMS207833-supplement-Supplementary_data.zip › Initial assessment/Pneumovax-23/Pneumovax23_Batch1_Vial4_1.jpg]

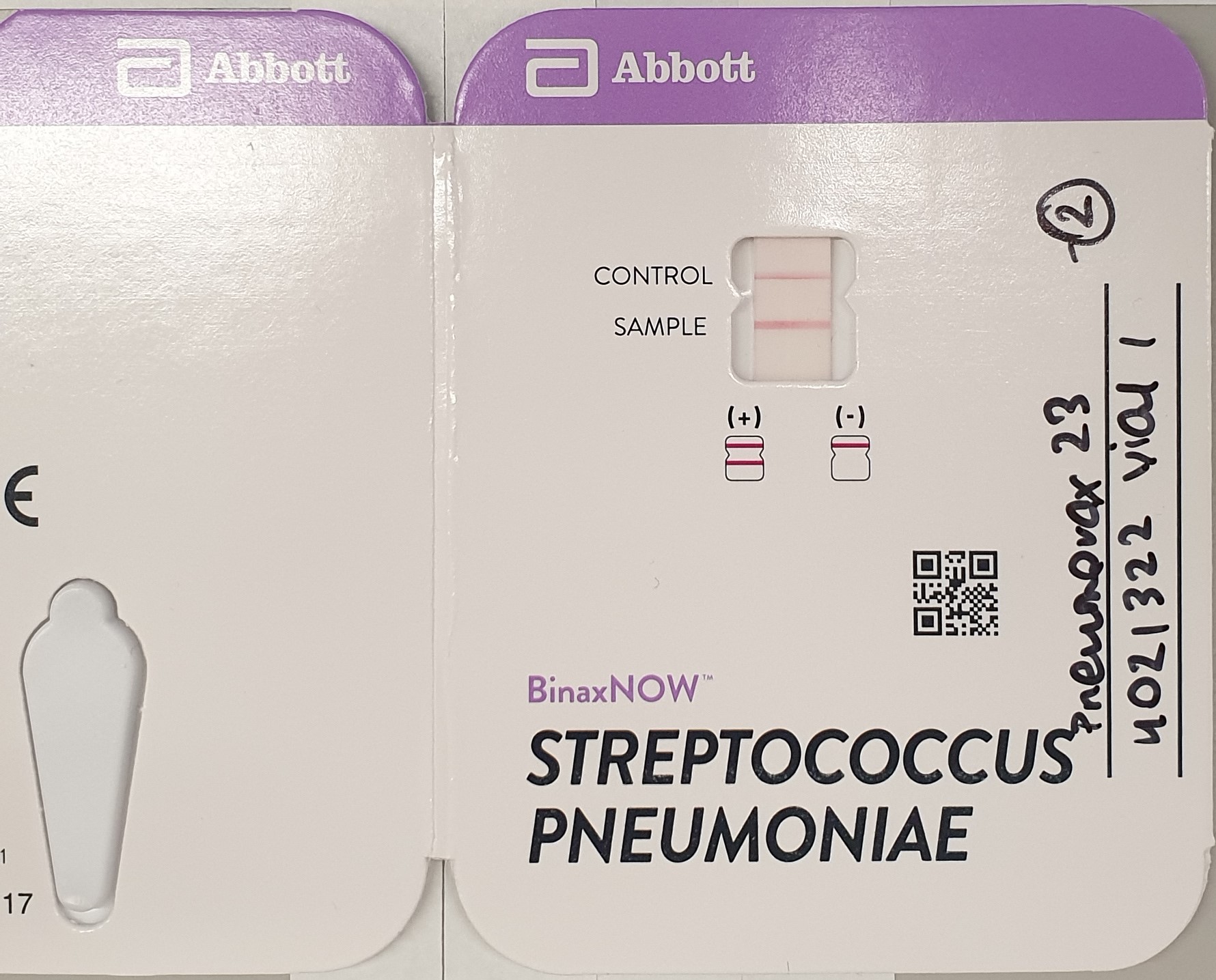

Supplement: Supplementary data [file EMS207833-supplement-Supplementary_data.zip › Initial assessment/Pneumovax-23/Pneumovax23_Batch1_Vial4_2.jpg]

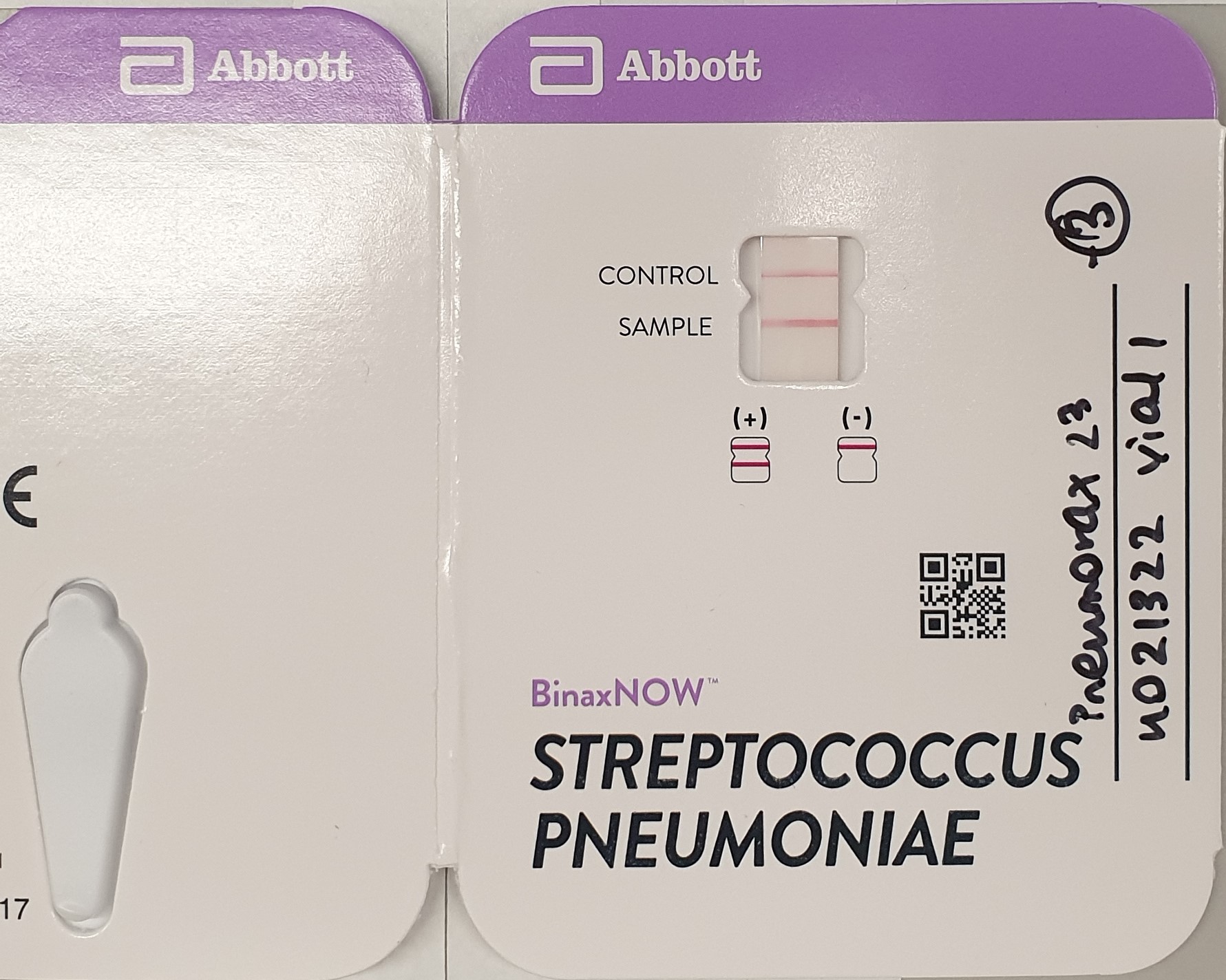

Supplement: Supplementary data [file EMS207833-supplement-Supplementary_data.zip › Initial assessment/Pneumovax-23/Pneumovax23_Batch1_Vial4_3.jpg]

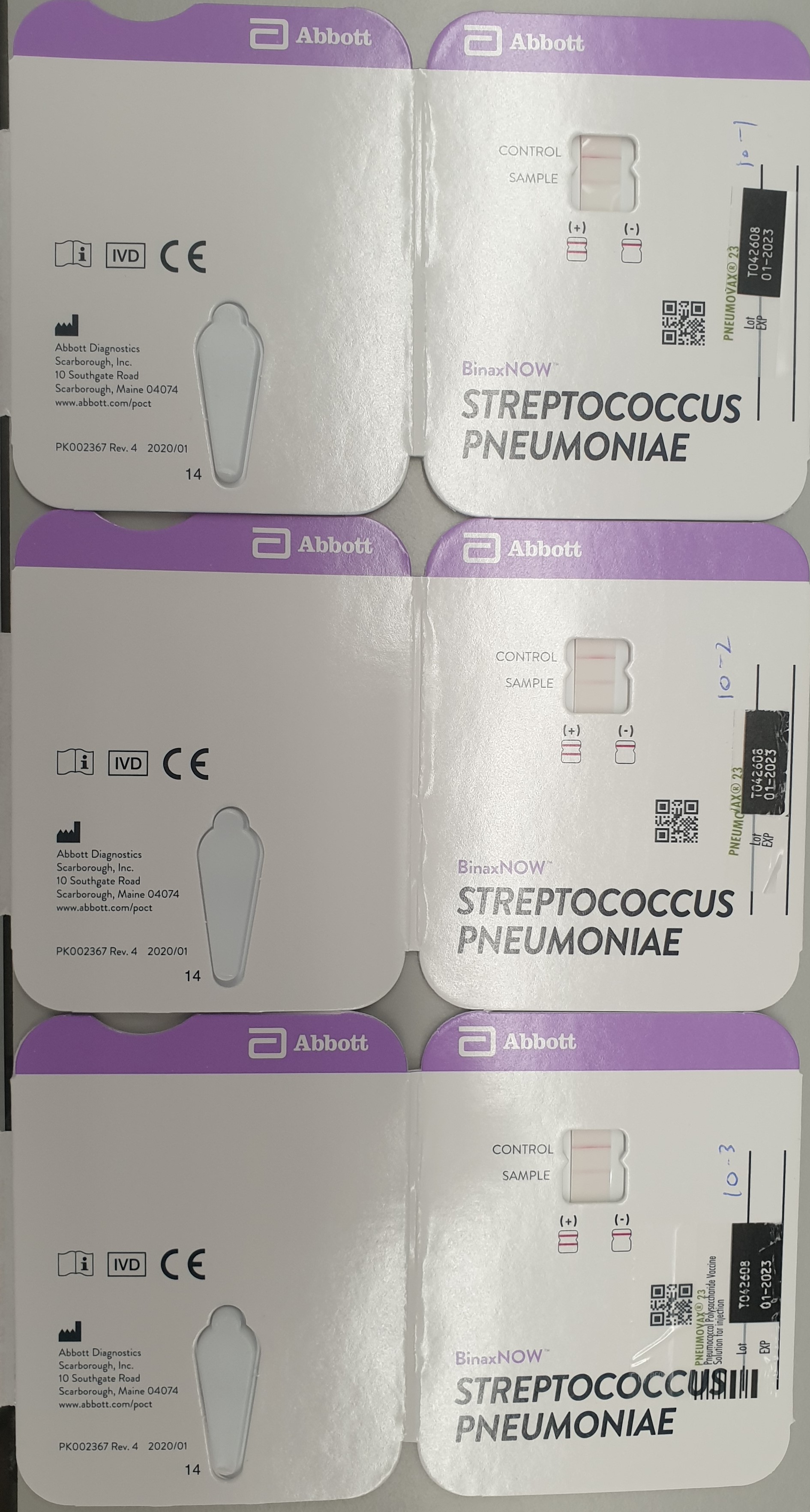

Supplement: Supplementary data [file EMS207833-supplement-Supplementary_data.zip › Initial assessment/Pneumovax-23/Pneumovax23_Batch2_Vial10_1-3.jpg]

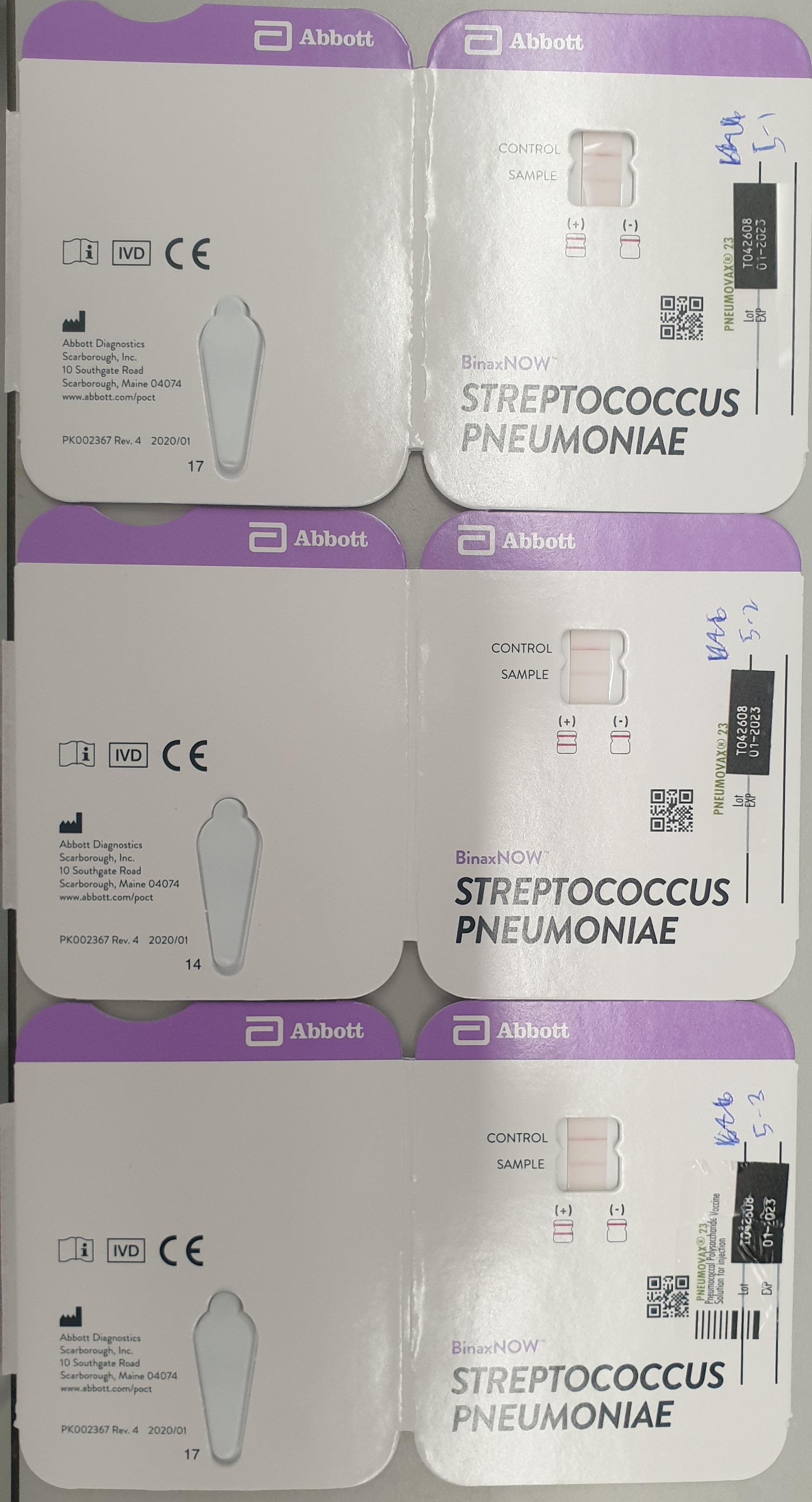

Supplement: Supplementary data [file EMS207833-supplement-Supplementary_data.zip › Initial assessment/Pneumovax-23/Pneumovax23_Batch2_Vial5_1-3.jpg]

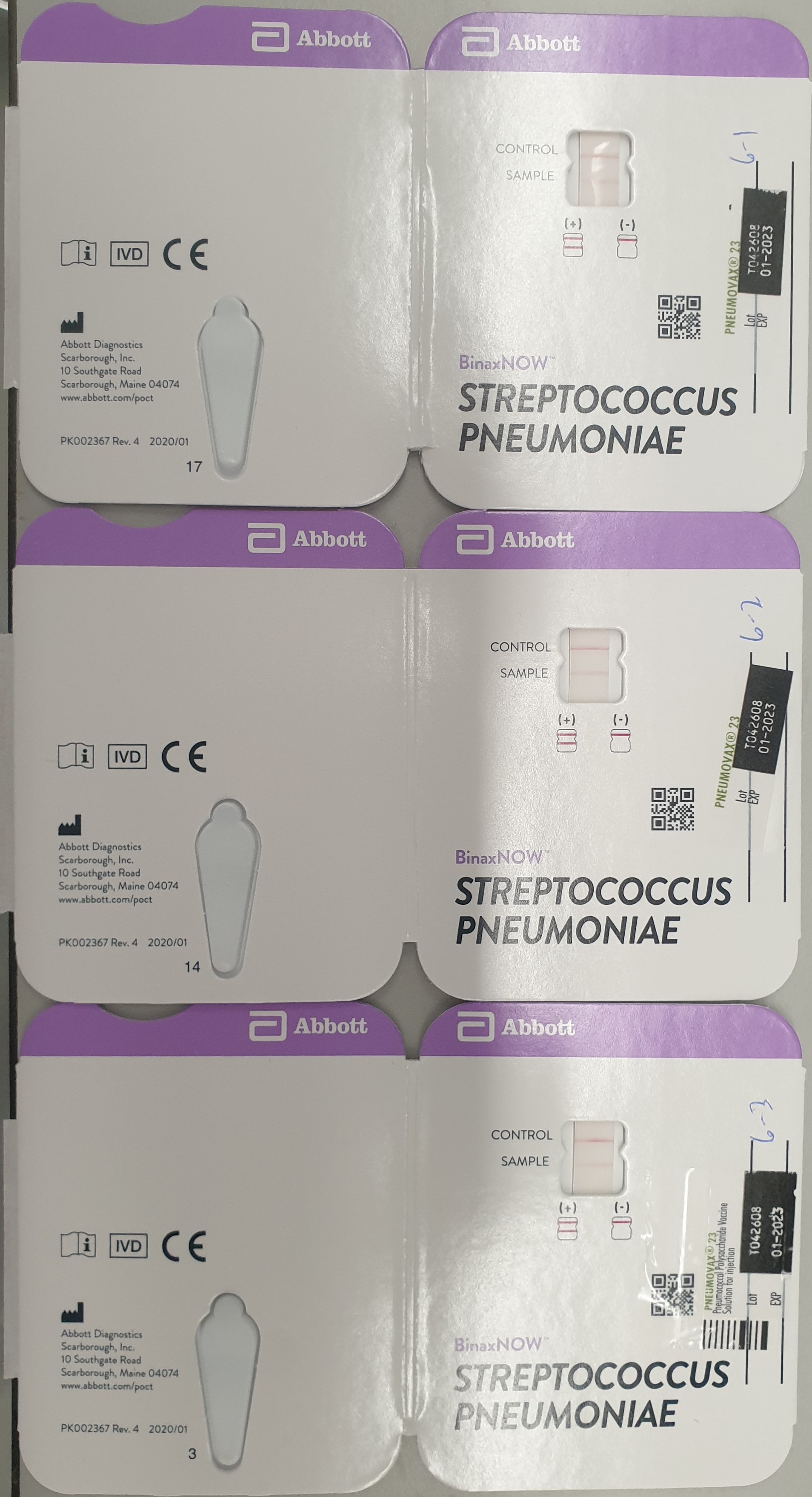

Supplement: Supplementary data [file EMS207833-supplement-Supplementary_data.zip › Initial assessment/Pneumovax-23/Pneumovax23_Batch2_Vial6_1-3.jpg]

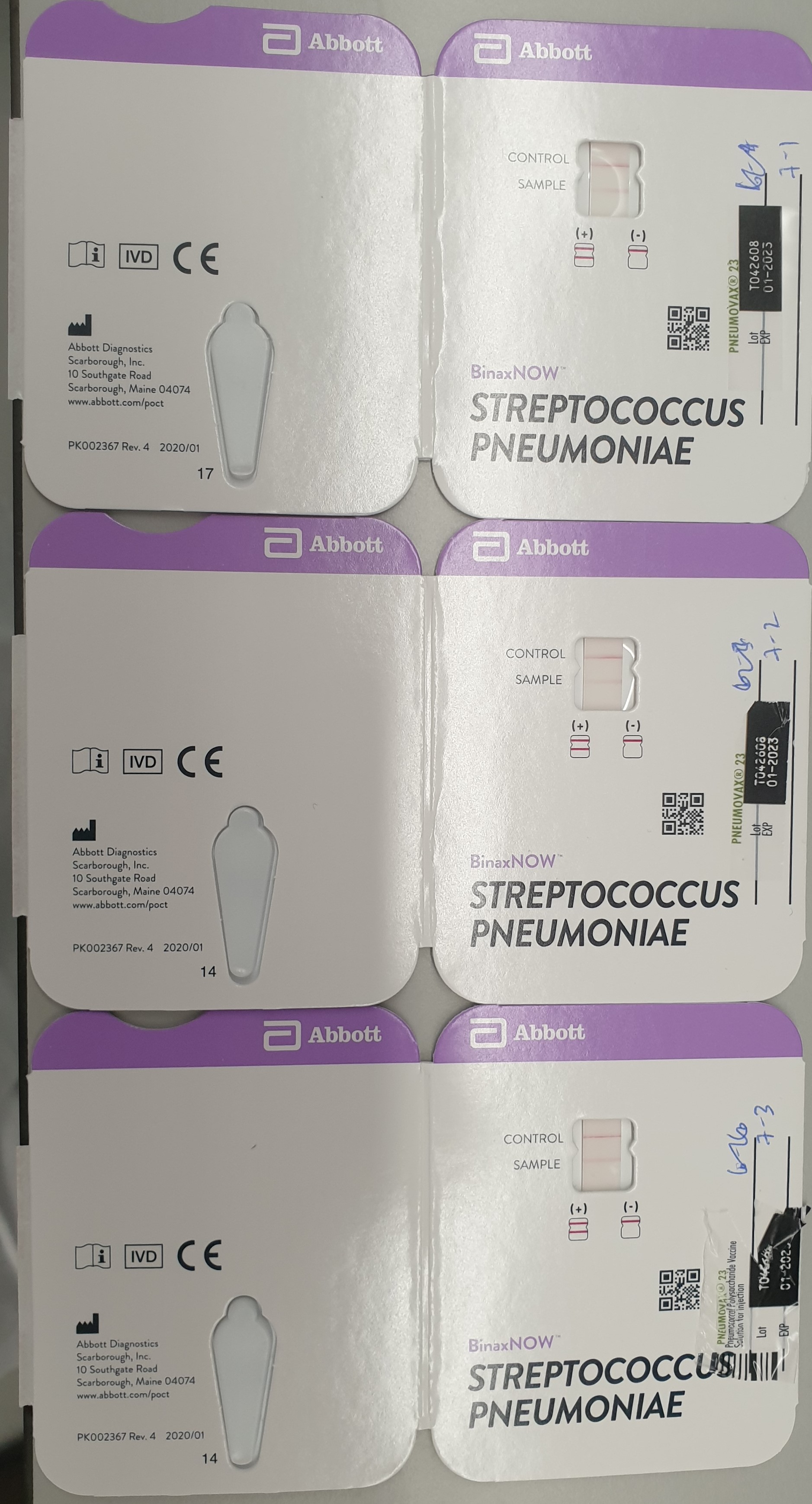

Supplement: Supplementary data [file EMS207833-supplement-Supplementary_data.zip › Initial assessment/Pneumovax-23/Pneumovax23_Batch2_Vial7_1-3.jpg]

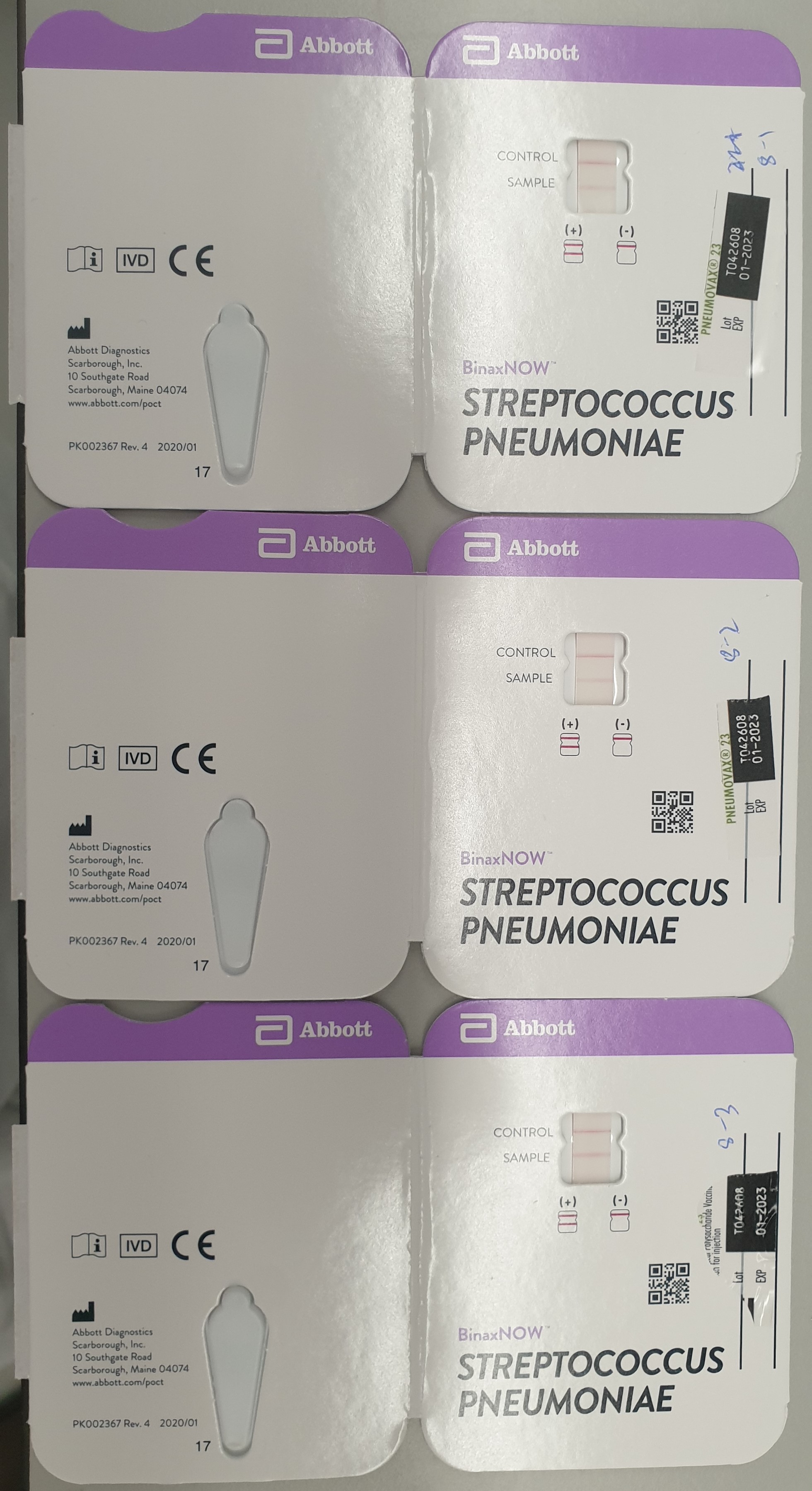

Supplement: Supplementary data [file EMS207833-supplement-Supplementary_data.zip › Initial assessment/Pneumovax-23/Pneumovax23_Batch2_Vial8_1-3.jpg]

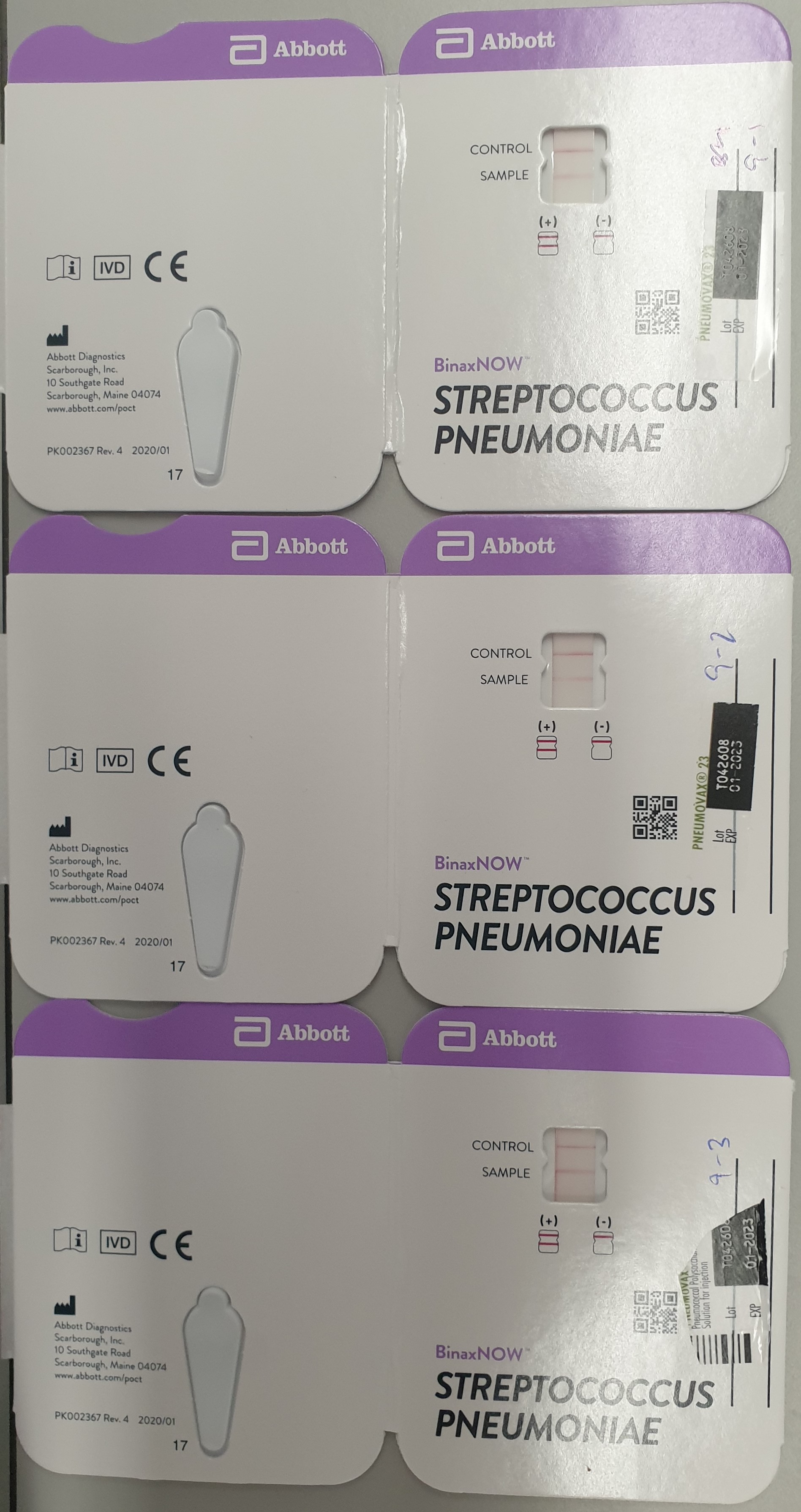

Supplement: Supplementary data [file EMS207833-supplement-Supplementary_data.zip › Initial assessment/Pneumovax-23/Pneumovax23_Batch2_Vial9_1-3.jpg]

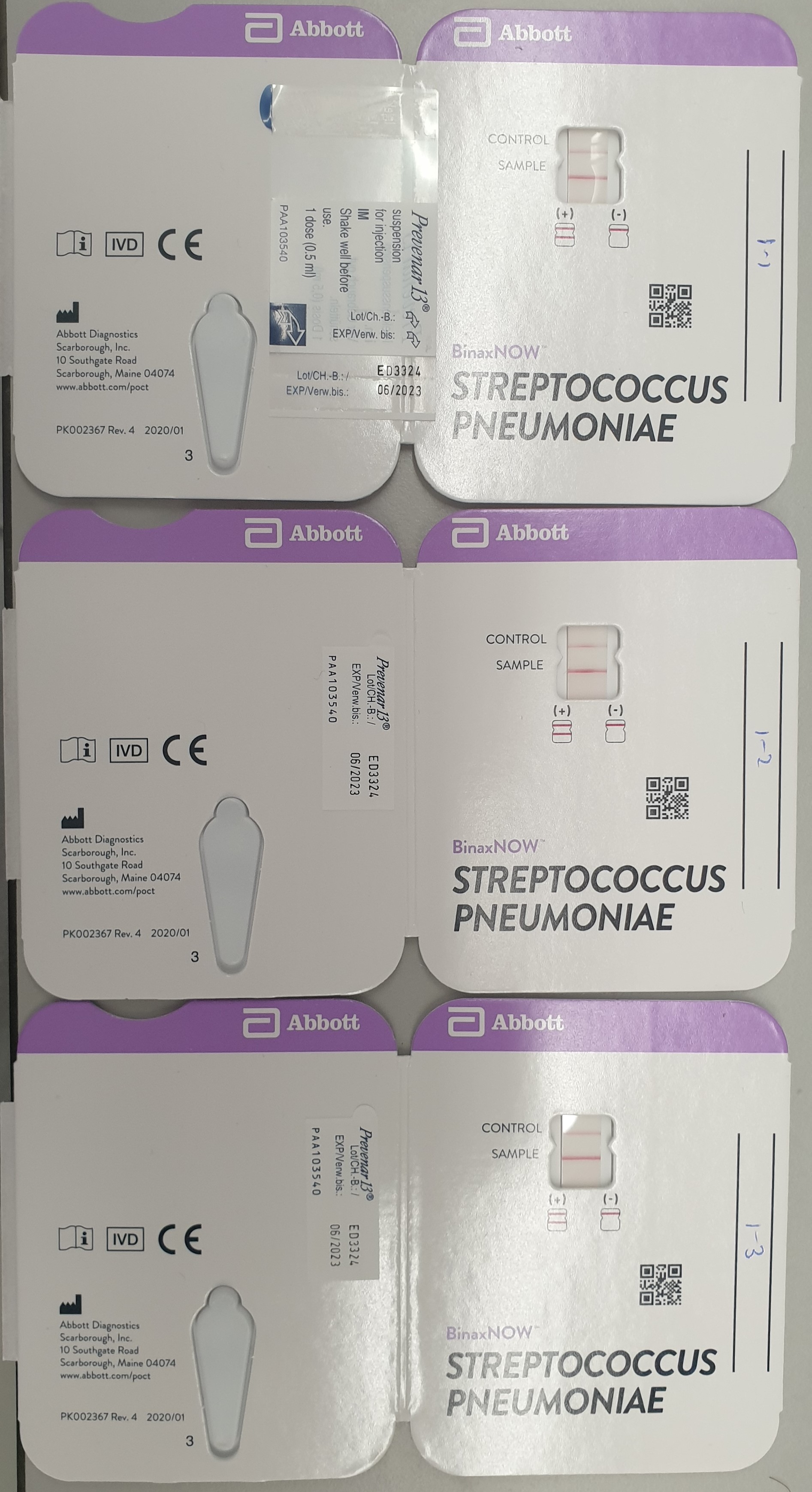

Supplement: Supplementary data [file EMS207833-supplement-Supplementary_data.zip › Initial assessment/Prevenar-13/Prevenar13_Batch1_Vial1_1-3.jpg]

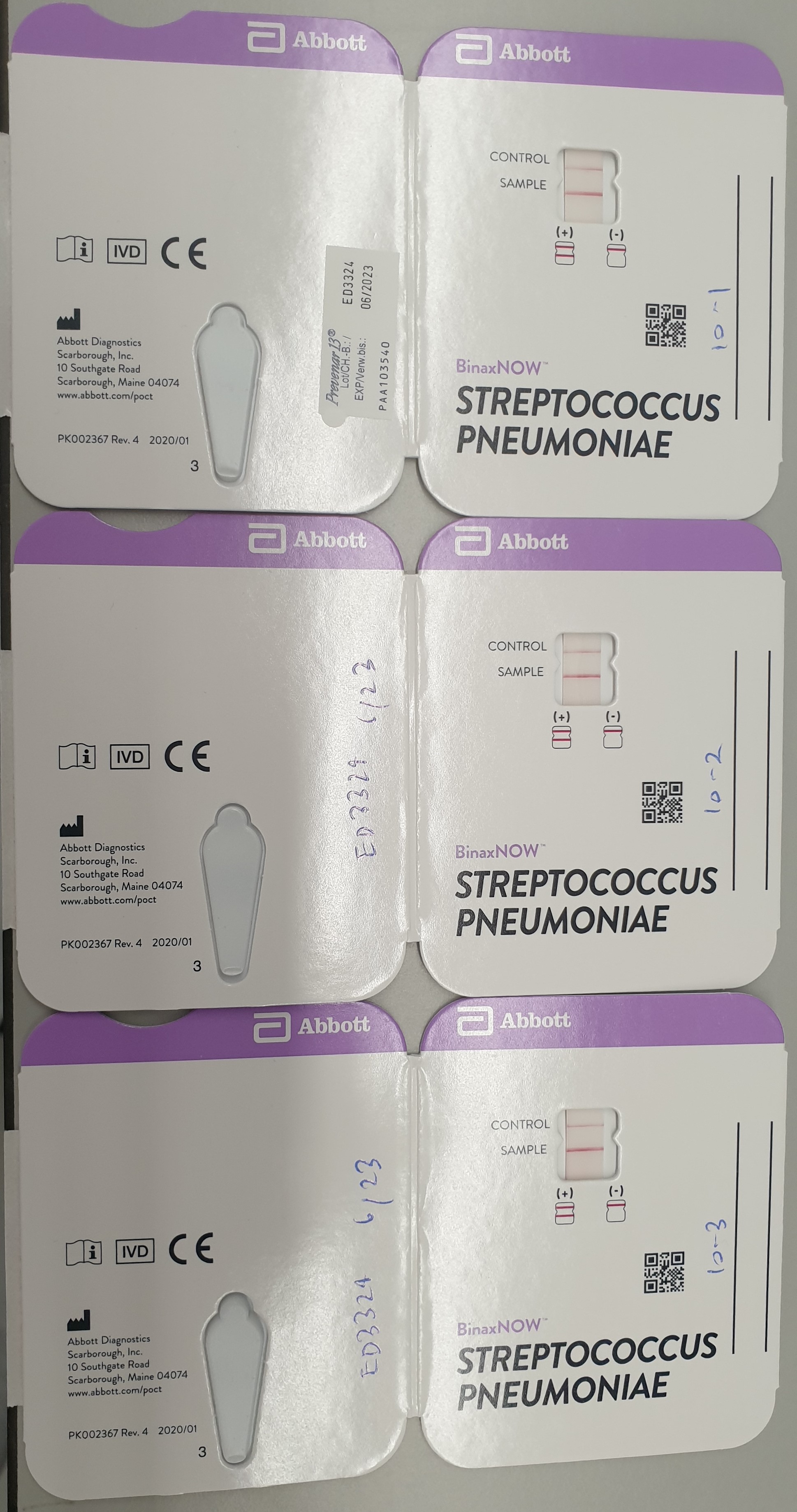

Supplement: Supplementary data [file EMS207833-supplement-Supplementary_data.zip › Initial assessment/Prevenar-13/Prevenar13_Batch1_Vial10_1-3.jpg]

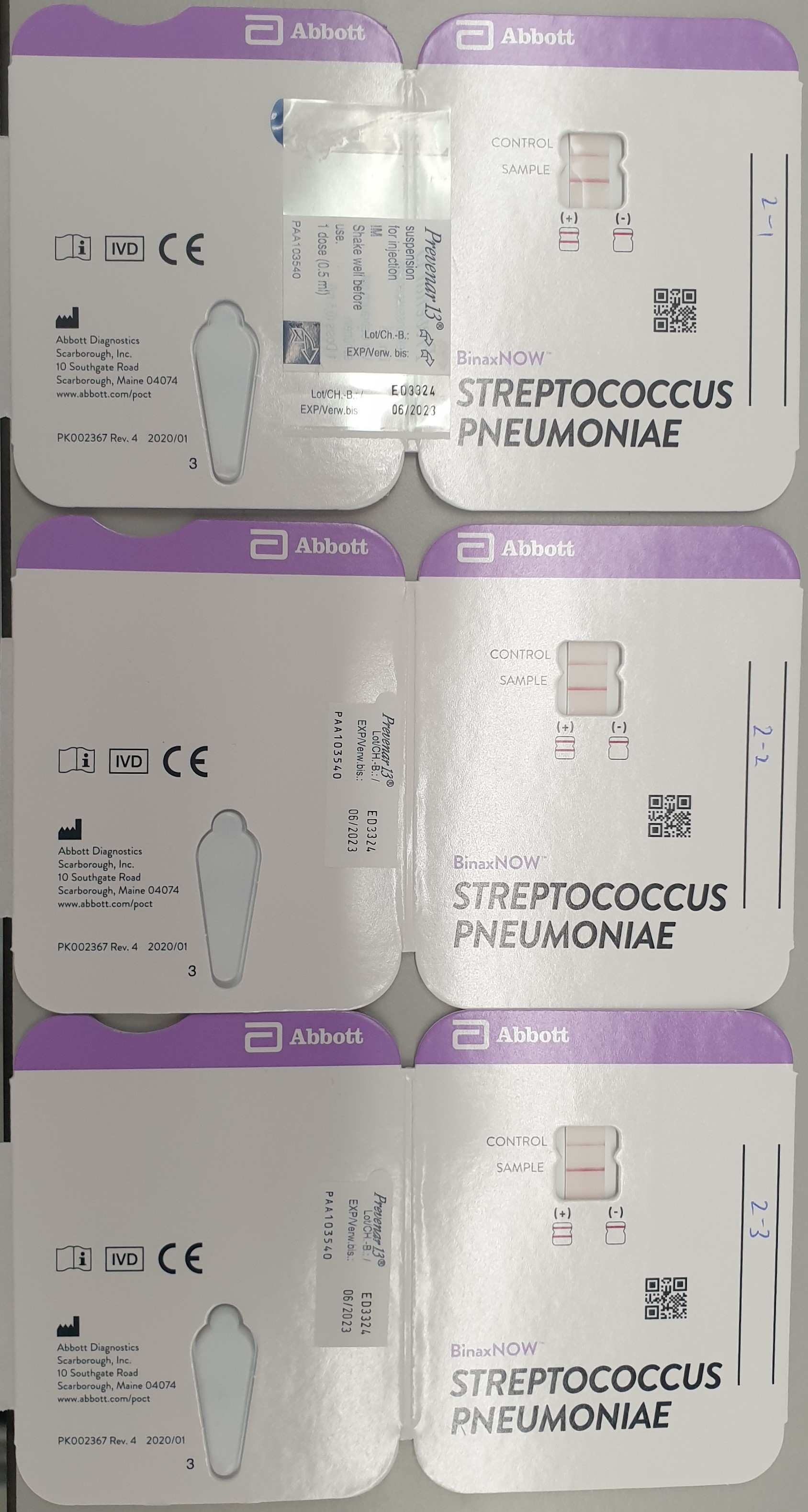

Supplement: Supplementary data [file EMS207833-supplement-Supplementary_data.zip › Initial assessment/Prevenar-13/Prevenar13_Batch1_Vial2_1-3.jpg]

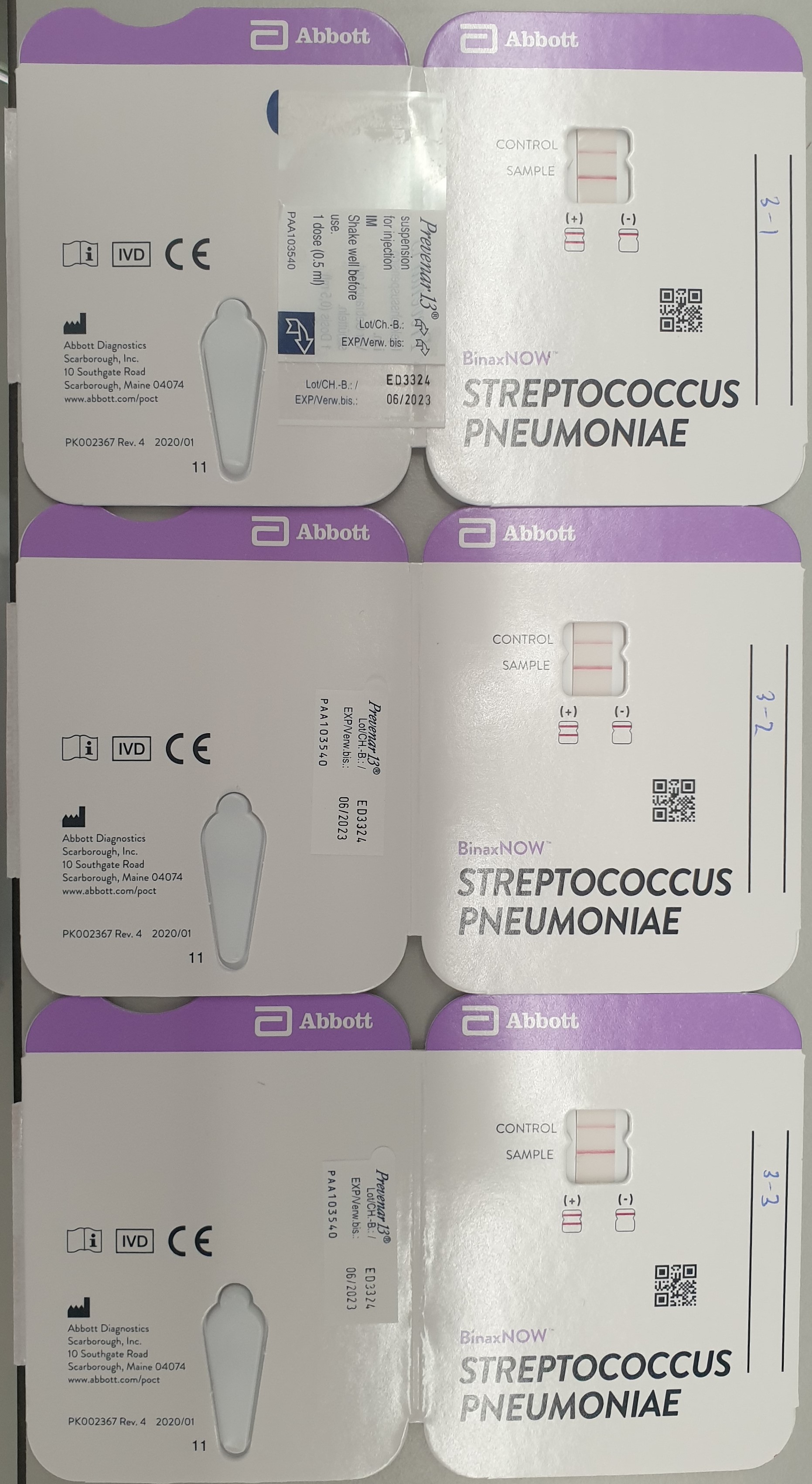

Supplement: Supplementary data [file EMS207833-supplement-Supplementary_data.zip › Initial assessment/Prevenar-13/Prevenar13_Batch1_Vial3_1-3.jpg]

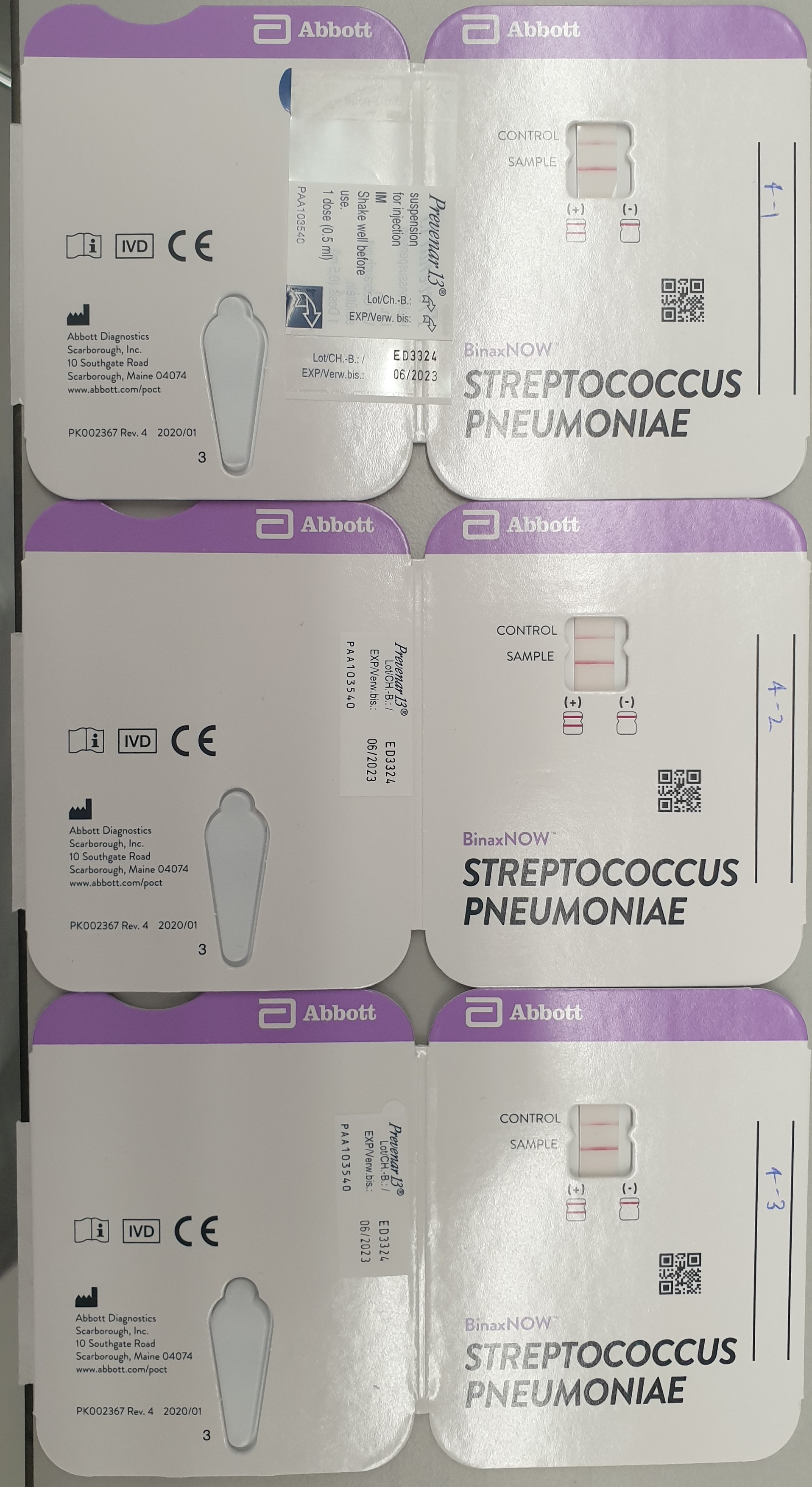

Supplement: Supplementary data [file EMS207833-supplement-Supplementary_data.zip › Initial assessment/Prevenar-13/Prevenar13_Batch1_Vial4_1-3.jpg]

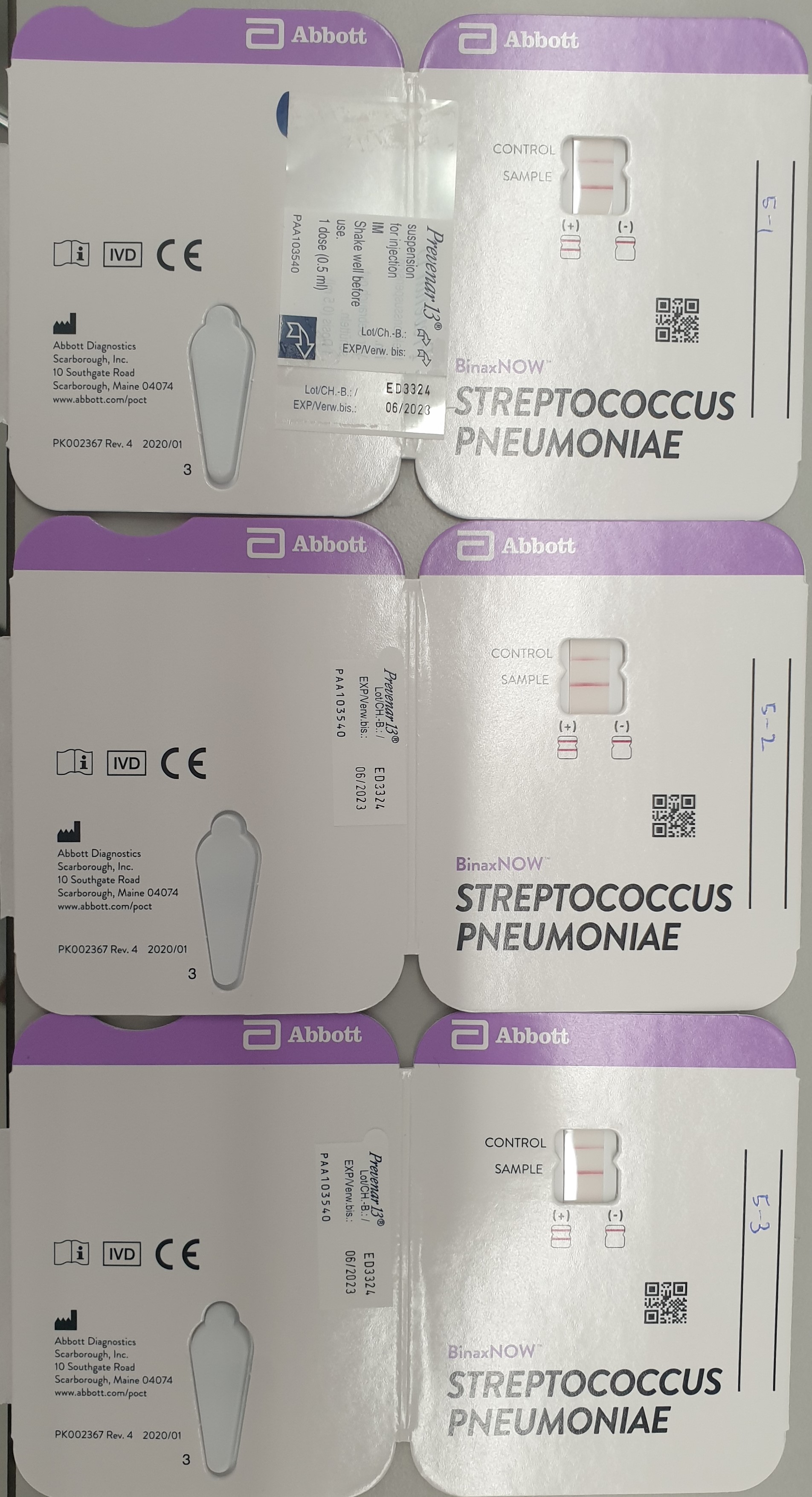

Supplement: Supplementary data [file EMS207833-supplement-Supplementary_data.zip › Initial assessment/Prevenar-13/Prevenar13_Batch1_Vial5_1-3.jpg]

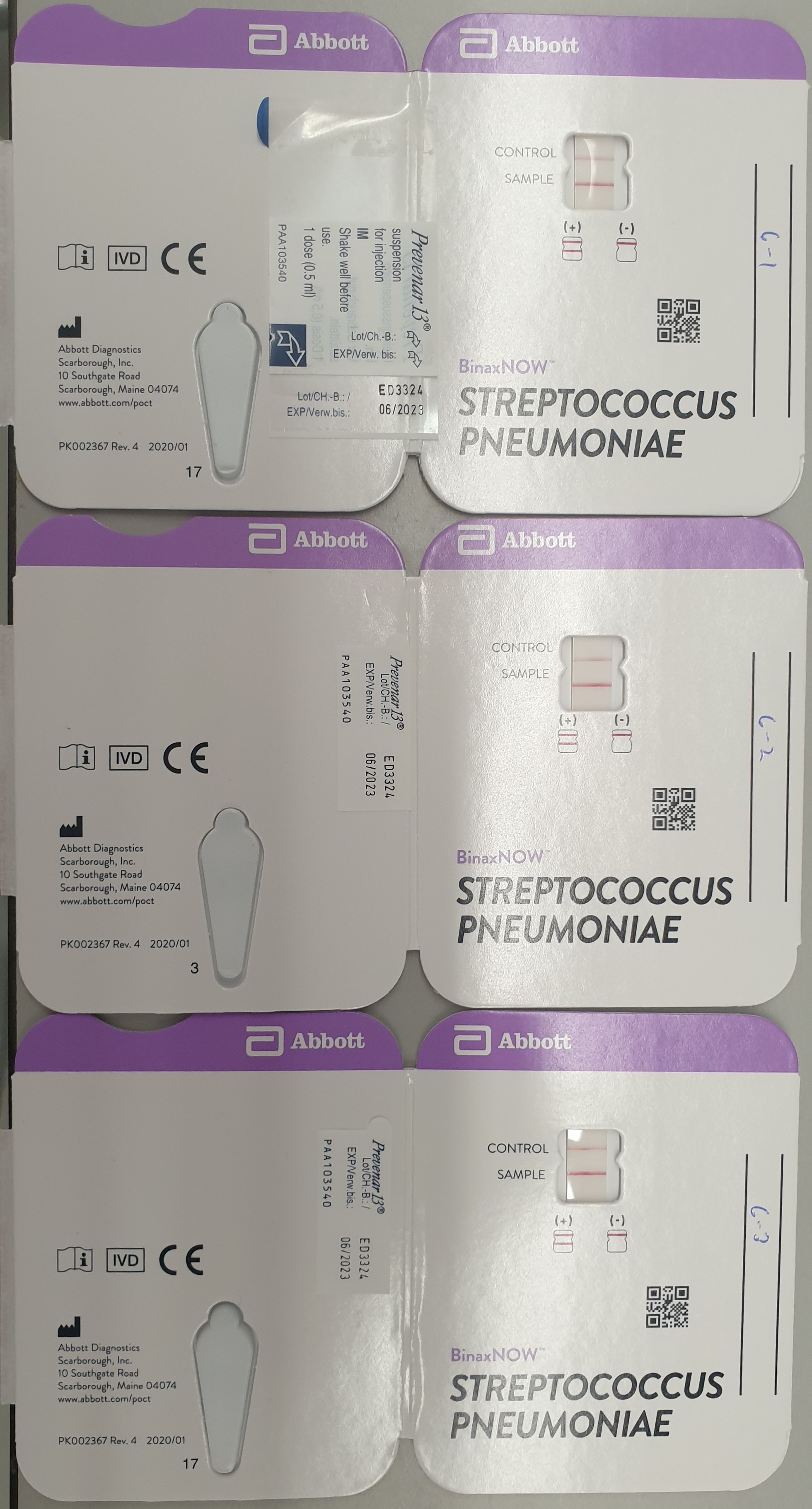

Supplement: Supplementary data [file EMS207833-supplement-Supplementary_data.zip › Initial assessment/Prevenar-13/Prevenar13_Batch1_Vial6_1-3.jpg]

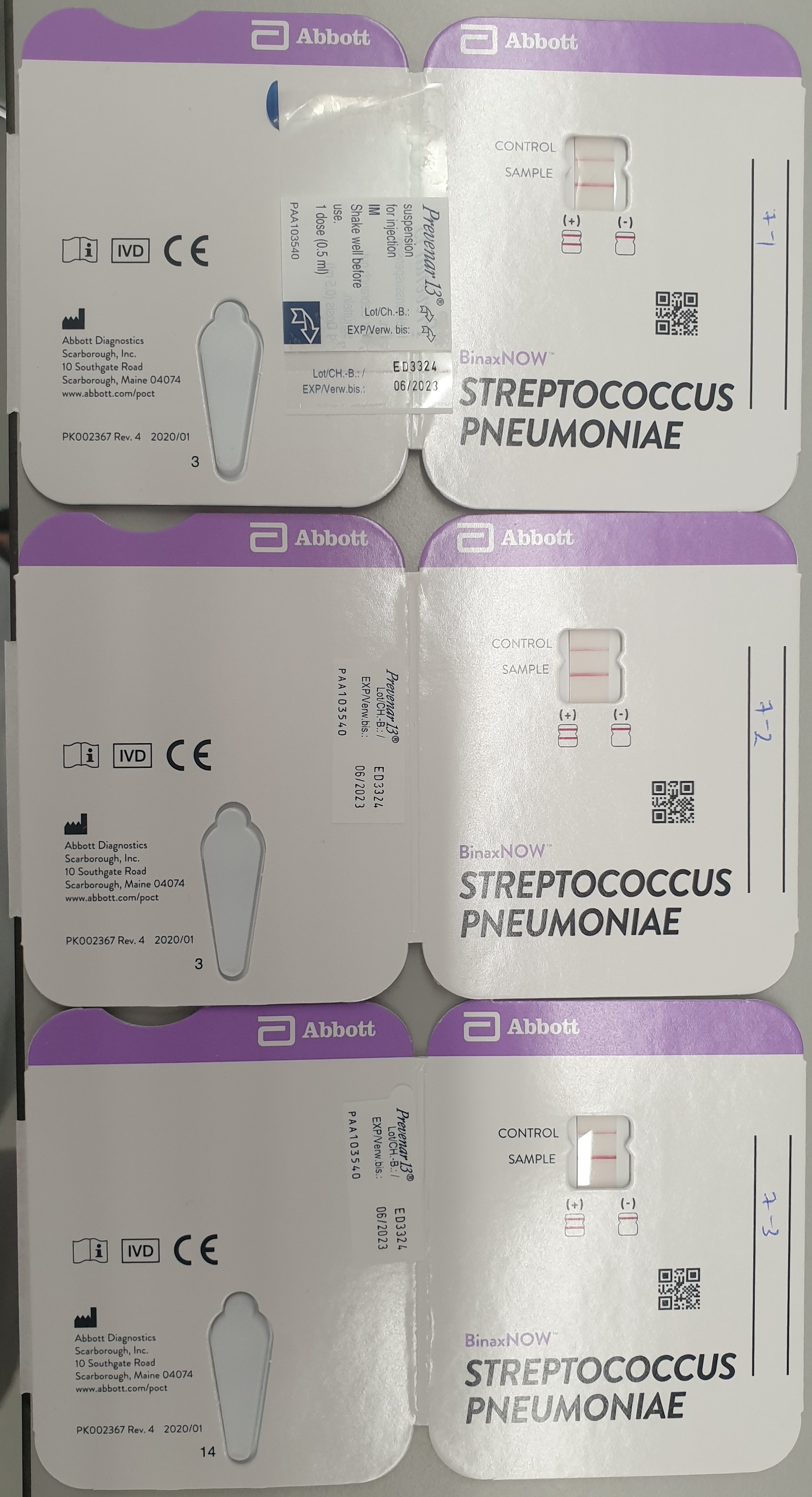

Supplement: Supplementary data [file EMS207833-supplement-Supplementary_data.zip › Initial assessment/Prevenar-13/Prevenar13_Batch1_Vial7_1-3.jpg]

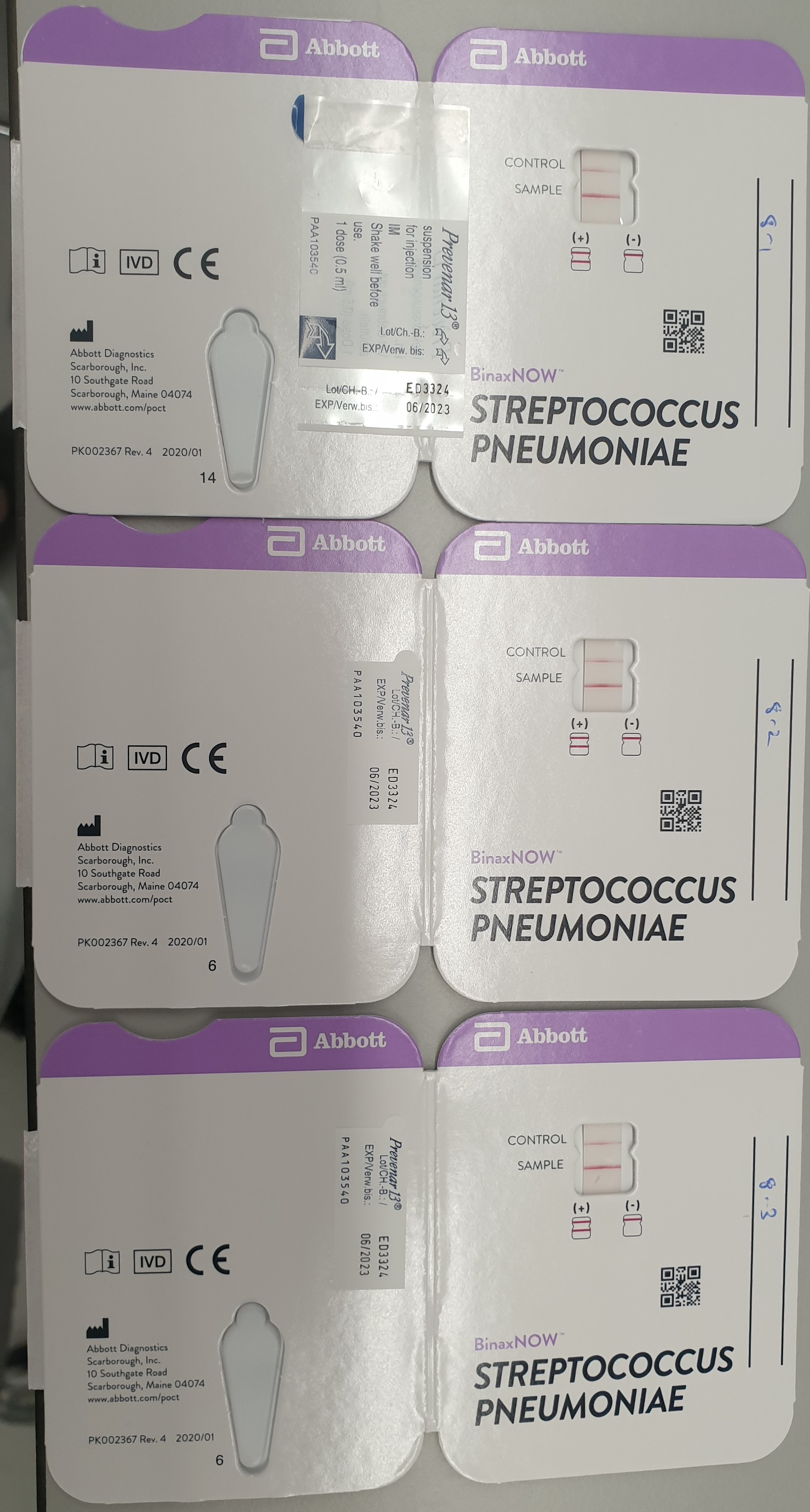

Supplement: Supplementary data [file EMS207833-supplement-Supplementary_data.zip › Initial assessment/Prevenar-13/Prevenar13_Batch1_Vial8_1-3.jpg]

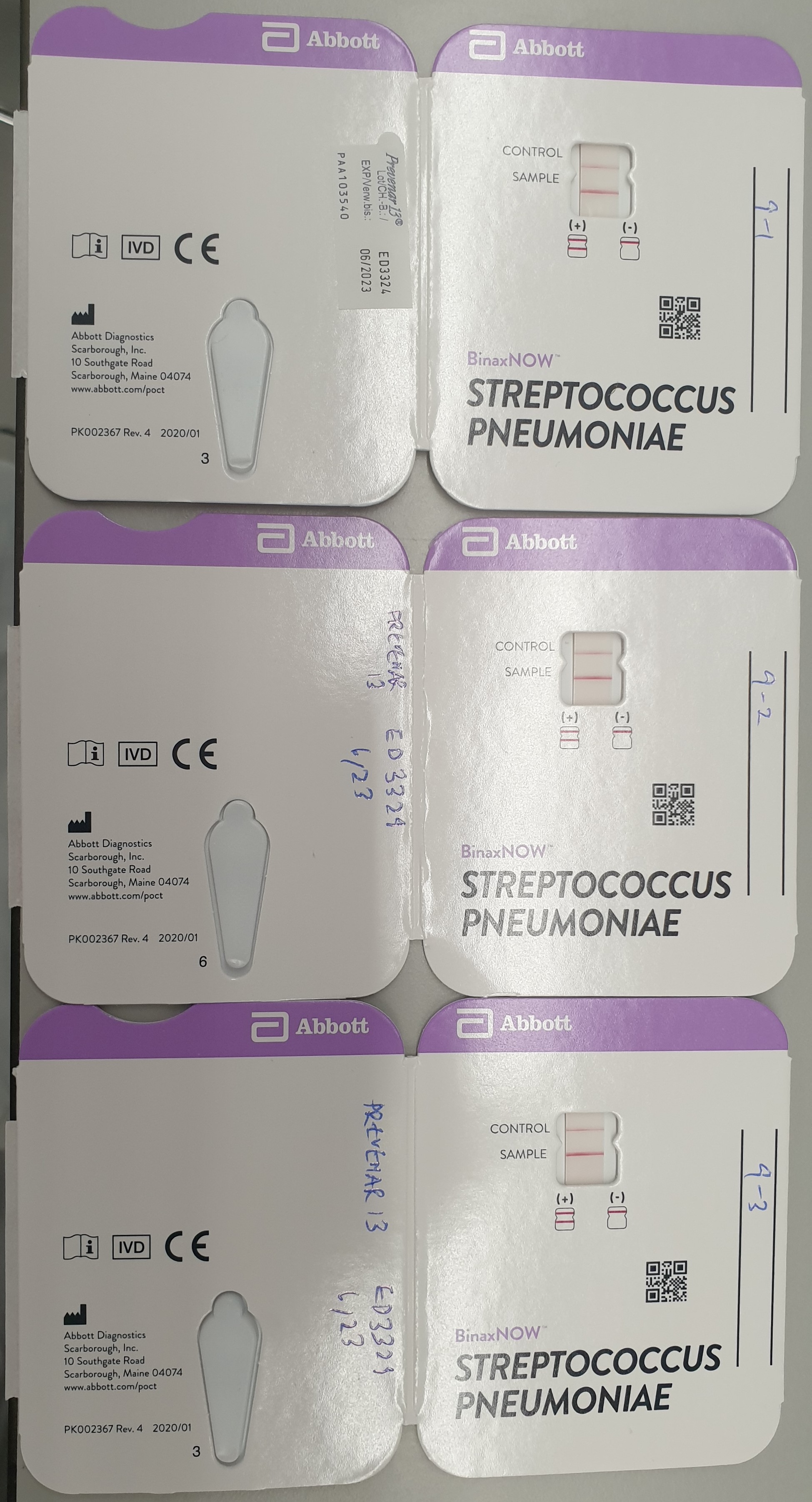

Supplement: Supplementary data [file EMS207833-supplement-Supplementary_data.zip › Initial assessment/Prevenar-13/Prevenar13_Batch1_Vial9_1-3.jpg]
